# Nursing regulation in Canada: Insights from a scoping review

**DOI:** 10.1371/journal.pone.0323716

**Published:** 2025-05-16

**Authors:** Patrick Chiu, Natalie J. Thiessen, Sobia Idrees, Kathleen Leslie, Janice Y. Kung

**Affiliations:** 1 Faculty of Nursing, College of Health Sciences, University of Alberta, Edmonton, Alberta, Canada; 2 Faculty of Health Disciplines, Athabasca University, Athabasca, Alberta, Canada; 3 Geoffrey and Robyn Sperber Health Sciences Library, University of Alberta, Edmonton, Alberta, Canada; Health Researcher, SPAIN

## Abstract

Canadian nursing regulators have implemented various innovations to reform regulatory approaches to address workforce challenges, system demands, governance reforms, and a drive for efficiency. Given the significant role that regulators play in influencing patient safety, workforce, and health system outcomes, decision-making must be evidence-informed. This review examined the nature, extent, and range of literature related to nursing regulation in Canada and how the existing scholarship aligns with emerging regulatory and health system trends to inform the development of a research agenda. The review was conducted following the JBI methodology for scoping reviews and with the support of a medical research librarian. A total of 195 sources, including scholarly (n = 156, 80%) and grey literature sources (n = 39, 20%), were included and reviewed. Just over half of the included scholarly sources were empirical studies (n = 80, 51.3%). The number of publications has steadily increased over the last few decades, and the majority of sources were situated in a provincial/territorial context (n = 104, 53.3%), followed by the national (n = 67, 34.4%) and global context (n = 24, 12.3%). The majority of the literature was led by scholars or professional groups unaffiliated with nursing regulators (n = 129, 66.2%). In contrast, nursing regulators led, commissioned, or were involved in a smaller portion of sources (n = 66, 33.8%). The largest category of literature focused on regulatory models, governance structures, and reforms (n = 59, 30.3%); followed by registration and licensure (n = 57, 29.2%); nursing roles and standards (n = 53, 27.2%); conduct, complaints, and discipline (n = 13, 6.7%); continuing competence programs (n = 11, 5.7%); and education program approval/accreditation (n = 2, 1%). The current literature base related to nursing regulation in Canada is largely descriptive and non-empirical and may not provide sufficient evidence to inform regulatory decision-making. This highlights opportunities for regulators and researchers to enhance collaboration to co-create research agendas that can maximize knowledge development and mobilization efforts.

## Introduction

### Rationale

In Canada, the regulation of healthcare professionals is governed by provincial and territorial legislation. Health professions are largely self-regulated, where governments delegate professional regulatory responsibility to a body governed mostly by members of a profession [1]. Self-regulatory bodies act on behalf of the government and are mandated through legislation to act in the public interest by ensuring the delivery of safe, competent, ethical, and high-quality healthcare [2,3]. Under Canada’s federated system, the provinces and territories have unique legislative frameworks for regulating healthcare professionals. Legislative frameworks define which professions are regulated, establish their outer scope of practice, and specify the structures and processes for regulation. This includes governance structures, licensure requirements, continuing competence programs, education program approvals, the development and enforcement of standards of practice and codes of ethics/conduct, and procedures for addressing complaints and professional misconduct [2]. Provincial and territorial legislation – in combination with regulators’ bylaws, governance policies, and regulatory processes – form the regulatory frameworks designed to regulate professionals in the public’s interest.

There are four nursing designations recognized in Canada: licensed practical nurses (LPN) (alternatively designated as registered practical nurses (RPN) in the province of Ontario), registered psychiatric nurses (RPN), registered nurses (RN), and nurse practitioners (NP). In some provincial and territorial jurisdictions, these designations are regulated by a single nursing regulatory body, while in others, each designation is regulated separately. RPNs have historically only existed in western provinces and territories, including the Northwest Territories and Nunavut, British Columbia, Alberta, Saskatchewan, and Manitoba [4]. However, this nursing designation has recently been introduced in the Atlantic provinces. At the time of writing, there are 22 separate nursing regulatory bodies across Canada. Consequently, despite sharing a common mandate, Canadian nursing regulatory bodies vary in their regulatory approaches, governance models, and structures.

Canadian nursing regulators operate within a much broader field of health practitioner regulation provincially, nationally, and internationally and are influenced by shifting sociopolitical and economic contexts [3,5]. In the nineteenth century, self-regulation was predominantly focused on negotiations between two parties - governments and professionals [3]. Many Canadian regulators represented both public and professional interest mandates. Starting in the twentieth century, a growing number of state actors and public members have become increasingly involved in matters of professional regulation as concerns about abuses of professional power and tensions between professional groups grew [3]. Since that time, health practitioner regulatory systems in Canada have continued to navigate significant tensions with governments, the public, professional associations, unions, and employers, all calling for greater transparency, accountability, efficiency, and effectiveness [3]. Growing attention to regulatory reform and modernization has been influenced by many factors, such as high-profile professional conduct cases covered in the media [6–8], inquiries into regulatory failure [9,10], and workforce challenges [11].

Significant changes in provinces such as British Columbia are likely to influence the pace and scope of changes across the country. British Columbia’s *Health Professions and Occupations Act*, enacted in 2022, is one of the most significant regulatory reforms in Canada’s history [12]. This legislative change has already impacted other provinces, such as Nova Scotia, where Bill 323 aims to establish a common legislative foundation for regulated health professions [13]. In both these cases, legislative change includes the amalgamation of regulatory bodies, changes to governance structures, increased oversight measures, and significant changes to the framework guiding regulatory processes. These changes show increasing political interest and investment in health practitioner regulation reform, which are driving continued change in the Canadian regulatory landscape [14,15].

Canadian nursing regulators have been under the spotlight given their role in regulating the largest portion of the health workforce and their direct role in influencing the supply and quality of nurses across health systems. Since the COVID-19 pandemic, nursing regulators have faced additional pressure to modernize, harmonize, and rapidly adapt regulatory approaches to meet current health system needs [16]. While nursing regulators have implemented a variety of policy and practice changes to address these concerns, it is unclear what evidence exists to support decision-making and the extent to which reforms have been evaluated.

Previous reviews have examined health practitioner regulation literature in various contexts, including national, international, and interprofessional settings, such as those published by Browne et al. [17] and Leslie et al. [18]. These authors report the literature base to be diffuse, primarily descriptive, and insufficient for drawing strong conclusions about the effects of various regulatory approaches, policies, and practices on patient safety, the health workforce, and health system outcomes [17,18]. Additionally, the applicability of existing evidence is limited by its lack of generalizability across different contexts, as regulatory schemes vary significantly due to social, political, economic, and historical factors. Consequently, more targeted knowledge syntheses on health practitioner regulation at the profession or country level are particularly useful for guiding decision-making and shaping policy and research agendas. Further, there is evidence that regulatory systems in other comparable countries, such as the United Kingdom (UK) [19], the United States (US) [20,21], and Australia [22] have developed strong research agendas to guide evidence-based regulatory research and evaluation. As a result, gaining an understanding of the scope of the existing literature base relevant to Canadian nursing regulation can help identify gaps and inform the development of a targeted research agenda to support the advancement of a strong nursing regulatory system.

### Research questions

The overarching objective of this review was to explore the breadth and depth of the literature related to nursing regulation in Canada. The research questions that guided our review were:

a) What is the nature, extent, and range of available scholarship informing the regulation of nurses within the Canadian context?
b) How does the extant scholarship align with emerging health practitioner regulation trends, and what are the knowledge gaps?

## Methods

### Overview

We conducted a scoping review following the JBI methodology for scoping reviews [23]. We chose this methodology because it is appropriate for addressing broad research questions and summarizing the volume and nature of available literature in an emerging field of research [24]. Unlike a systematic review, which focuses on synthesizing high-quality research evidence from empirical studies to answer a narrow and well-defined research question, a scoping review is more exploratory and inclusive of diverse sources. Similarly, while an integrative review also includes diverse sources, a scoping review is better suited for establishing the landscape of a topic, clarifying key concepts and definitions, and identifying knowledge gaps to inform areas requiring additional research [24].

This review was registered with the Open Science Framework on January 8, 2024, and a protocol was published in JMIR Research Protocols [25]. Our review was organized into six stages including the following: 1) identifying the research questions and aligning it with the review objective; 2) identifying relevant studies using an inclusion and exclusion criteria that aligned with the research objective and questions; 3) selecting relevant studies using a planned approach to evidence searching, selection, data extraction, and the presentation of evidence; 4) using both descriptive statistics and qualitative content analysis to chart the data; 5) consulting subject matter experts in regulation through professional networks; and 6) collating, summarizing, and reporting the evidence.

### Eligibility

#### Population.

Our population of interest included nurses of any designation in Canada (LPNs, RPNs, RNs, and NPs). To capture a wide range of evidence and scholarship, we also included literature that discussed regulated nurses within the broader context of other regulated healthcare professionals.

#### Concept.

Our key concept was nursing regulation. We drew on the definition of regulation provided by Benton et al.: “all those legitimate, appropriate and sustained means whereby order, identity, consistency, control, and accountability are brought to practitioners through legally enforced, professional and/or voluntary action resulting in enhanced protection of the public, efficient and effective trans-jurisdictional movement, and the ongoing re-alignment of professional practice to patient and societal needs” [26]. We included literature that focused on the core functions of professional regulation, including education program accreditation or approval, registration and licensure, standards of practice and code of ethics/conduct development and enforcement, continuing competence programs, and discipline and conduct, as well as regulatory models, governance, and reform.

#### Context.

Regulatory schemes vary significantly across jurisdictions due to social, political, economic, and historical factors. Our review focused on nursing regulation in the Canadian context as the goal of this review was to inform the development of a research agenda to advance nursing regulation in Canada.

#### Types of sources.

All study methods, including quantitative, mixed-methods, and qualitative, were eligible to be included in the review. Peer-reviewed discussion papers, commentaries, and opinion papers were included if they provided a substantive exploration, examination, or critique of nursing regulation in Canada. Non-empirical sources were included as they provide valuable context, theoretical insights, and critical perspectives that complement empirical findings [23].

Grey literature such as theses/dissertations, policy documents, and reports from the websites of Canadian organizations relevant to nursing regulation were also included. Theses/dissertations were included given the potential for in-depth exploration of nursing regulatory issues. Systematic or other reviews were excluded, but we screened the reference lists for relevant studies. Books and unavailable sources were excluded. Given the focus on identifying the nature, extent, and range of scholarship focused on nursing regulation in Canada, we did not place limitations on publication dates. We excluded non-English papers due to translation resource constraints. The inclusion and exclusion criteria are provided in S1 Appendix: Inclusion/Exclusion Screening Form.

### Search terms, strategy, and sources

A medical librarian (JYK) developed and executed comprehensive searches in Ovid MEDLINE, Ovid Embase, CINAHL, Scopus, Web of Science Core Collection, and ProQuest Dissertations and Theses Citation Index on March 15, 2024. Keywords and controlled vocabulary were carefully selected to capture relevant literature about nursing regulation in Canada. A geography filter was applied to ensure all Canada-related terms and its 100 largest centres [27] were included in the search (see S2 Appendix: Database Searches). In addition to our database search, we identified literature through chain searching by reviewing the reference lists of all included articles and excluded systematic reviews during screening. We also hand-searched the Journal of Nursing Regulation given its relevance to the research topic and screened the first 200 hits on Google Scholar using the keywords ‘nursing regulation’ and ‘Canada’.

We sought relevant grey literature by reviewing: a) the websites of each provincial and territorial nursing regulatory body; b) nursing regulatory federations (Canadian Council of Registered Nurse Regulators, the Canadian Council of Practical Nurse Regulators, and the Registered Psychiatric Nurse Regulators of Canada); c) national professional organizations (Canadian Nurses Association, Canadian Association of Schools of Nursing, and the Canadian Nurses Protective Society - the primary liability insurance provider for regulated nurses in Canada); d) regulatory organizations (Canadian Network of Agencies of Regulation and the UK's Professional Standards Authority [PSA]); and e) newsletters and legal updates from law firms focused on professional regulation in Canada.

While the PSA is not a Canadian organization, we included this organization given our knowledge that some nursing regulators have conducted regulatory reviews and reforms using guidance developed through the PSA. Following this comprehensive search, we consulted professional regulation experts within our network who identified four additional sources that met our inclusion criteria.

### Study/source of evidence selection

**Fig 1:**
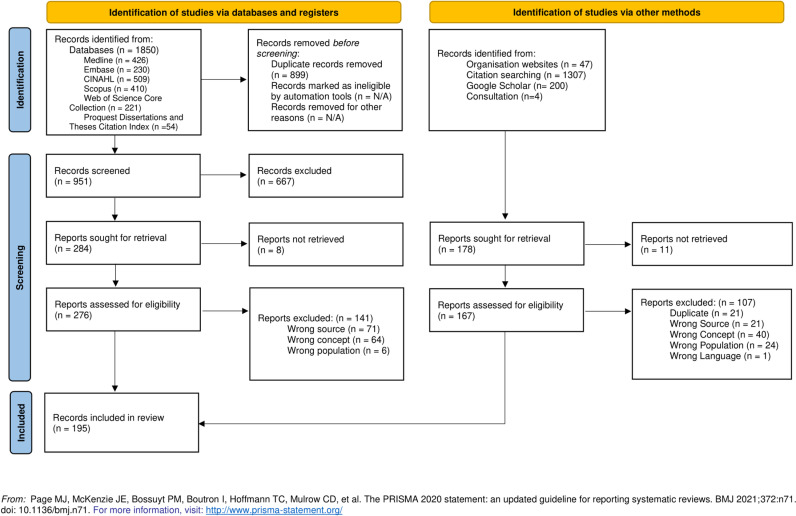
PRISMA Flow Diagram.

Retrieved records from databases were uploaded into a collaborative review management software system, Covidence [28]. We piloted the inclusion and exclusion criteria using 10% of the records and clarified terminology as needed. Study selection followed a two-level screening process. First, two independent reviewers (NT, SI) screened the titles and abstracts against our inclusion and exclusion criteria. Second, the same independent reviewers (NT, SI) screened the full-text articles against the inclusion criteria. Disagreements during primary and secondary screening were resolved with a third reviewer (PC) and through consensus meetings. Records retrieved through websites, citation searching, Google Scholar, and expert consultation were screened in the same manner. To ensure the quality of reporting in this scoping review, we followed the Preferred Reporting Items for Systematic Reviews and Meta-Analyses Extension for Scoping Reviews (PRISMA-ScR) guidelines (S3 Appendix: Preferred Reporting Items for Systematic Reviews and Meta-Analyses Extension for Scoping Reviews Checklist). All records retrieved through the search strategy, including those included and excluded during primary and secondary screening, and records from other sources, are documented using an adapted PRISMA flow diagram to enhance the review’s transparency and reproducibility (Fig 1).

### Data extraction and analysis

Our data extraction was guided by an extraction tool. After piloting the extraction tool using 10% of the included articles and reports, we revised the tool to create additional data fields. Based on our review of the included articles and reports, we further refined the extraction form through an iterative process to include additional units of analysis. For example, we created additional fields to facilitate the categorization of articles and reports based on whether the research questions or discussion focused on regulatory structures, processes, or outcomes - an approach used in a previous large-scale global review on health practitioner regulation [18]. We added more fields in the extraction form to record and analyze the areas of focus to provide insight into where there may be gaps in the literature base. Given regulators’ growing interest in research, we also assessed the extent to which the literature was regulator-led versus academic-led. During the screening process, it became clear that the scholarship on nursing regulation was heavily focused on certain provinces and territories. We were interested in identifying where the scholarship was most concentrated, and added a field to capture this observation.

Two independent reviewers (NT, SI) completed data extraction using a Google spreadsheet, and all extracted data were reviewed and verified by an additional reviewer (PC). To help contextualize our findings, we further identified whether nursing regulation was the main focus of the article or report, or whether it was explored within the context of a broader subject matter. Quality appraisals were not performed, given the goals of this review. A summary of extracted data is provided (S4 Appendix: Data Extraction from Scholarly and Grey Literature Sources).

We employed a mix of directed and conventional content analysis [29] to identify and map the key purposes, aims, findings, and concepts within the included articles and reports. We first used a deductive process to categorize sources into six key focus areas. These categories were selected based on the common functions of health practitioner regulators within statutory regulatory schemes [30]. We also completed an inductive analysis, identifying subtopics under each of the six key focus areas. The deductive and inductive analyses were completed by two reviewers (NT, PC) and verified through consensus meetings. Below, we present the findings using descriptive statistics and a narrative summary.

## Results

We retrieved a total of 3408 records: 1850 through our database search and 1558 through other sources. After removing duplicates and screening all records for eligibility, we included a total of 195 sources: 135 from our database search and 60 from other methods. A list of excluded sources at full text can be found in S5 Appendix: Sources Excluded Following Full-Text Review.

### Characteristics of sources

Sources were categorized by publication type, year of publication, regulator involvement, geographical focus, and provincial and territorial focus (Table 1).

**Table 1:** Characteristics of Sources.

Characteristics	N (%)
**Publication Type** **Scholarly literature**	**156 (80%)**
Empirical study	80 (51.3%)
Discussion papers	63 (40.4%)
Commentary	9 (5.8%)
Editorial	3 (1.9%)
Letter to the editor	1 (0.6%)
**Grey literature**	**39 (20%)**
Thesis/Dissertation	11 (28.2%)
Regulatory review/audit	10 (25.6%)
Research study report	9 (23.1%)
Consultation report	4 (10.3%)
Historical review	3 (7.7%)
Legal commentary	2 (5.1%)
**Type of Methods for Empirical Studies**	
Qualitative	45 (56.3%)
Mixed-methods	17 (21.3%)
Quantitative	7 (8.8%)
Multi-methods	7 (8.8%)
Surveys (Unspecified qualitative or quantitative)	1 (1.25%)
Environmental scan	1 (1.3%)
Comparative analysis	1 (1.3%)
Historical analysis	1 (1.3%)
**Year of Publication**	
1976-1979	1 (0.5%)
1980-1989	0
1990-1999	8 (4.1%)
2000-2000	28 (14.4%)
2010-2019	112 (57.4%)
2020-2024	46 (23.6%)
**Regulator Involvement**	
Yes	66 (33.8%)
No	129 (66.2%)
**Geographical Focus**	
Provincial/Territorial	104 (53.3%)
National	67 (34.4%)
Global	24 (12.3%)
**Provincial/Territorial Focus**	
Ontario	33 (31.7%)
British Columbia	27 (26%)
Alberta	12 (11.5%)
Nova Scotia	7 (6.7%)
Manitoba	6 (5.7%)
Saskatchewan	4 (3.8%)
Unspecified	2 (1.8%)
Newfoundland and Labrador	1 (1%)
New Brunswick	1 (1%)
Quebec	1 (1%)
Multiple territories/provinces	10 (9.6%)

#### Publication type.

We categorized sources based on two key publication types - a) scholarly literature (n = 156, 80%), which included peer-reviewed empirical studies, discussion papers, commentaries, and editorials, and b) grey literature (n = 39, 20%), which included non-peer-reviewed literature produced outside of traditional publishing channels.

Of the 156 (80%) sources identified as scholarly literature, the most common type were empirical studies (n = 80, 51.3%) followed by discussion papers (n = 63, 40.4%) [12,31–91], commentaries (n = 9, 5.8%) [15,92–99], editorials (n = 3, 1.9%) [100–102], and a letter to the editor (n = 1, 0.6%) [103].

Of the 80 sources categorized as a peer-reviewed empirical study, 45 used qualitative methods (56.3%) [3,5,16,104–145], 17 (21.3%) used mixed-methods [21,146–161], seven (8.8%) used quantitative methods [162–168], and seven (8.8%) used multi-methods [169–175]. Other methods that did not fit into the categories identified above given the level of detail provided or the language used in the source included an environmental scan (1.3%) [176], survey (1.3%) without specifying either qualitative or quantitative method used [177], comparative analysis (1.3%) [178], and historical analysis (1.3%) [179].

A total of 39 (20%) grey literature sources were included. To highlight the variation in grey literature, we organized these sources into categories defined through an iterative process. Regulatory reviews/audits included internal or external assessments of regulatory bodies’ policies, practices, and functions. Study reports included those that discussed primary research findings or included an analysis or review of existing research. Consultation reports included documents that reported on regulatory bodies’ public outreach to their registrants, the public, and system partners. Histories of nursing regulatory bodies were categorized as historical reviews, and blogs or short articles produced by legal experts were categorized as legal commentaries. The most common grey literature sources were theses/dissertations (n = 11, 28.2%) [180–190], regulatory reviews/audits (n = 10, 25.6%) [191–200], and research study reports (n = 9, 23.1%) [2,4,201–207]. This was followed by consultation reports (n = 4, 10.3%) [208–211], historical reviews (n = 3, 7.7%) [212–214], and legal commentaries (n = 2, 5.1%) [215,216].

#### Year of publication.

**Fig 2:**
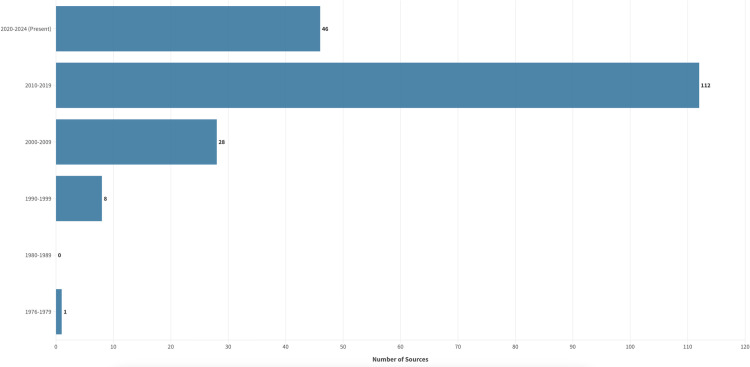
Year of Publication.

Sources included in the review were published between 1976–2024 (see a graphical representation of frequency in Fig 2). One source (0.5%) [54] was published between 1976–1979, eight (4.1%) sources between 1990–1999 [43,47,59,93,102,147,189,190], and 28 (14.4%) sources between 2000–2009 [31,33,35,37–39,46,57,60,69,74,76,83,87,90,105,106,110,129,130,138,151,156,162,185–187,204]. We identified a notable increase in literature over the last few decades with a highest number (n = 112, 57.4%) of sources published in the 2010s (2010–2019) [2–5,12,15,16,21,32,34,36,40–42,44,45,48–53,55,56,58,61–68,70–73,75,77–82,84–86,88,89,91,92,94–101,103,104,107–109,111–128,131–137,139–146,148–150,152–155,157–161,163–184,188,191–203,205–217] and 46 (23.6%) between 2020–2024 [2,3,5,12,15,16,21,40,42,48,51,52,56,64,78,80,81,99,103,108,111,112,114,131,134,135,137,140,141,143,150,155,173,182,188,192,194,196,198–200,206,212,213,215,216].

#### Involvement of regulators.

**Fig 3:**
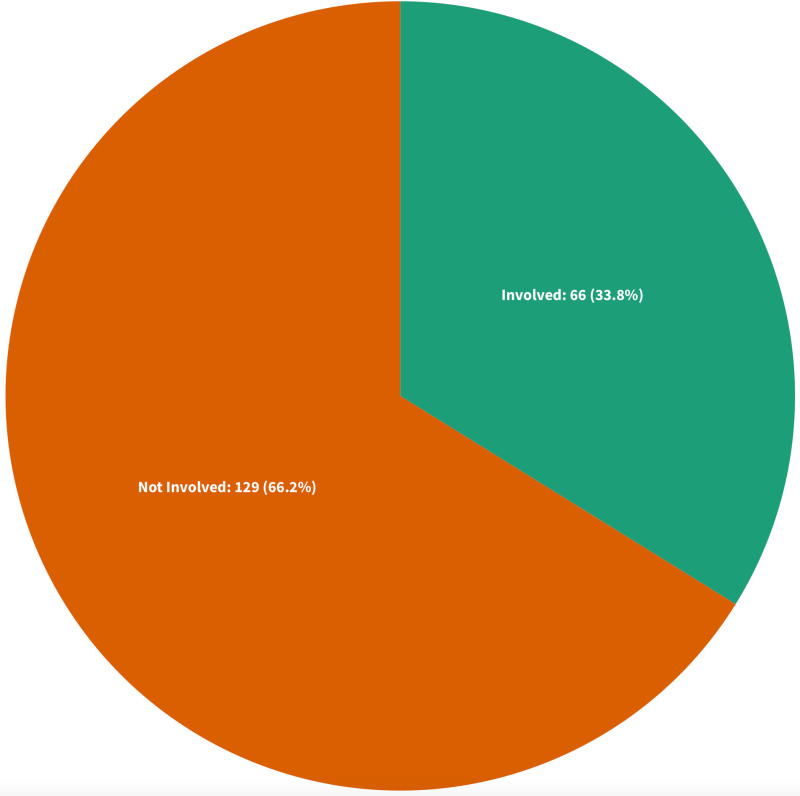
Regulator Involvement.

Sources with at least one author affiliated with a Canadian nursing regulatory organization, those explicitly stating they were commissioned or authored by such bodies, or those involving nursing regulators as research participants or task force members were categorized as publications involving nurse regulators. A total of 66 (33.8%) scholarly and grey literature sources were sorted into this category [4,34,36–38,40,44–46,49,51,53,55,56,61,64,65,67,70,75,76,79,86–90,96,105,139,142,153,157,160,163,164,168,174–177,180,183–185,191–197,199–201,204–206,208–214,217]. The remaining sources (n = 129, 66.2%) were led by scholars or professional groups unaffiliated with nursing regulators [2,3,5,12,15,16,21,31–33,35,39,41–43,47,48,50,52,54,57–60,62,63,68,69,71–74,77,78,80–85,91,93–95,97–104,106–138,140,141,143–152,154–156,158,159,161,162,165–167,169–173,178,179,181,182,186–190,198,202,203,207,215,216] Fig 3.

#### Geographical focus.

**Fig 4:**
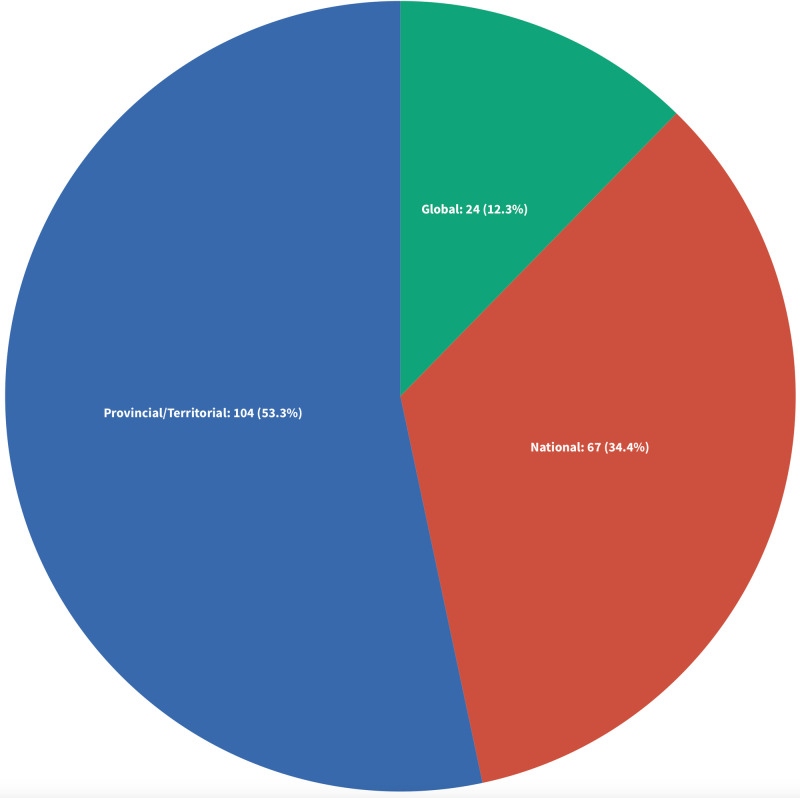
Geographical Focus.

**Fig 5:**
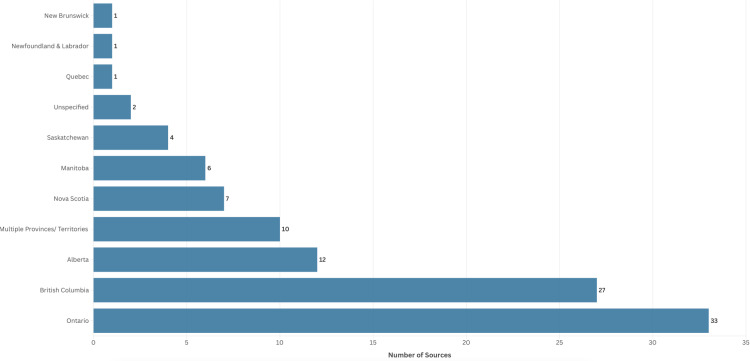
Provincial/Territorial Focus.

We categorized the included sources as having a provincial or territorial focus if they were situated in a specific province or territory or if they explored more than one province or territory (Figs 4 and 5). Sources that adopted a pan-Canadian lens were categorized as having a national focus. Those that included international comparisons were categorized as having a global geographical focus. More than half of the sources focused on nursing regulation at a provincial or territorial level (n = 104; 53.3%), with the province of Ontario being the most represented (n = 33, 31.7%) [31,37,38,43,47,56,57,69,78,84,89,90,95,105,107,118,122,128–131,139,144,153,160,162,164,180,185,187,188,190,195], followed by British Columbia (n = 27, 26%) [12,34,40,46,49,51–53,65,67,75,85,86,94,104,133,166,168,183,184,192,197,198,204,205,214,216], Alberta (n = 12, 11.5%) [44,70,76,157,159,163,165,174,189,196,212,213], Nova Scotia (n = 7, 6.7%) [33,45,142,208–211], Manitoba (n = 6, 5.7%) [36,116,191,194,199,200], and Saskatchewan (n = 4, 3.8%) [41,109,151,193]. The least number of sources were reported from Newfoundland and Labrador (n = 1, 1%) [119], New Brunswick (n = 1, 1%) [66], and Québec (n = 1, 1%) [102]. Ten sources (9.6%) focused on multiple provinces/territories with at least three or more provinces [5,64,110,112,120,125,137,181,206,207]. Two (1.8%) sources were at a provincial level but anonymized [136,145]. We categorized 67 (34.4%) sources as having a national focus [2,4,15,32,35,42,48,50,54,59,60,62,68,71–74,77,79–81,83,87,88,91,93,96–100,103,108,113,115,121,123,124,126,127,134,135,138,140,141,143,147,148,152,154,156,158,161,167,169–171,175–177,179,182,186,201,202,215,217] and 24 (12.3%) sources as having a global focus, which included international comparisons between Canada or specific provinces with other countries [3,16,21,39,55,58,61,63,82,92,101,106,111,114,117,132,146,149,150,155,172,173,178,203].

#### Regulatory structures, processes and outcomes.

In a previous integrative review by Leslie et al. [18], the authors used a modified Donabedian framework to categorize evidence on the design, delivery, and effectiveness of health practitioner regulation. This framework separates content into structures, processes, and outcomes. *Structures* refer to research examining contextual forces and characteristics shaping regulatory functions. *Processes* refer to research examining the activities of the regulatory system, including education and competency standards, registration/licensure and re-licensure, compliance monitoring, and workforce planning. *Outcomes* refer to research that measures the impact and effectiveness of regulatory systems and processes in improving the safety, quality, and efficiency of health systems and the workforce. Given the diversity of sources included, this framework offered a meaningful way to categorize the broad areas of inquiry, research questions, and discussions found within these sources.

**Fig 6:**
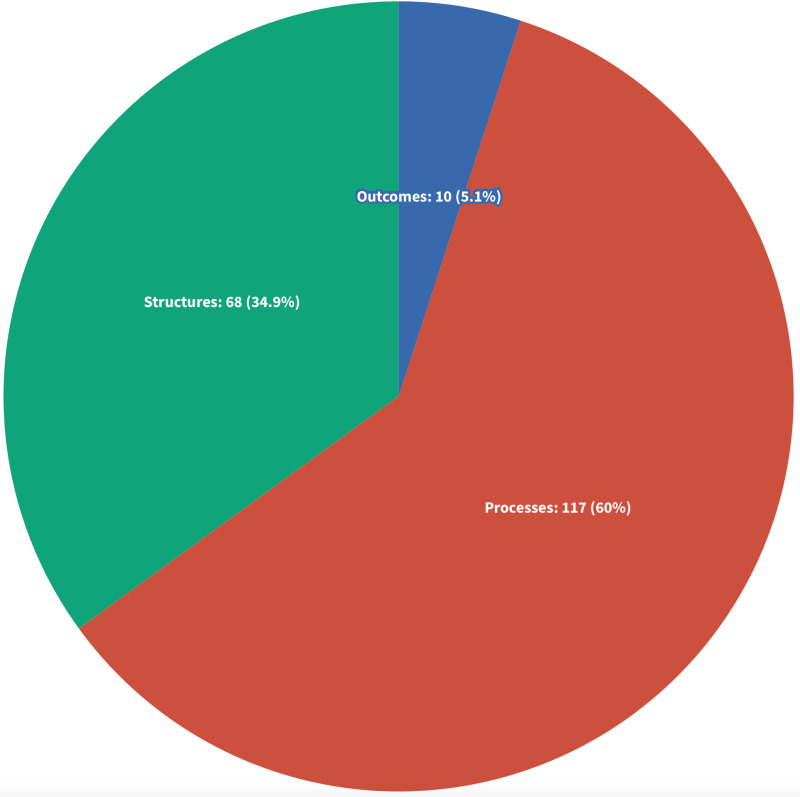
Literature Focus Areas According to Regulatory Structures, Processes, and Outcomes.

Of the 195 included sources, we identified 68 sources (34.9%) that discussed or examined regulatory *structures* [2–5,12,15,16,21,31,33,35,43,46–48,50–54,56,58–60,62,63,94,95,101,102,104,106,108–118,134,148–150,155,172,173,178,179,189,196,198,202–206,208–214,216]. The number of sources focused on regulatory structures have steadily increased since 2010, reflecting a growing emphasis on efficiency, accountability, and transparency in this field [5,12,51,94,198,208]. The majority of sources discussed or examined regulatory *processes* (n = 117, 60%) [32,34,36–42,44,45,49,55,57,61,64–68,70,72–93,96–100,103,107,119–122,124–133,135–147,151,152,154,156,158–166,168–171,174–177,180,181,184,186–188,190–195,197,199–201,207,215,217]. We categorized 10 sources (5.1%) under *outcomes* [69,71,105,123,153,157,167,182,183,185], with only one source using methods capable of examining causal relationships [157] Fig 6.

### Areas of focus

**Fig 7:**
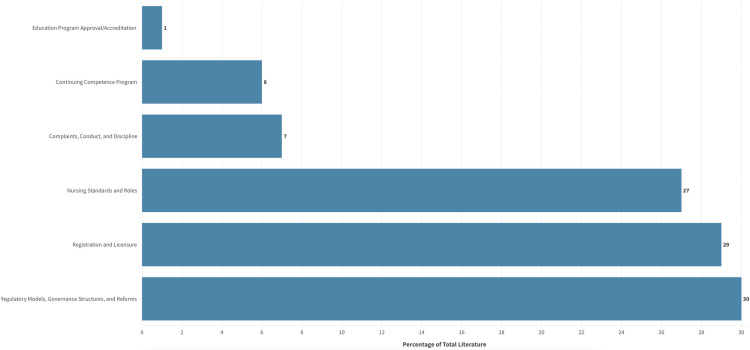
Publication per Focus Area.

Sources were additionally categorized into six focus areas, each representative of core regulatory functions common to professional regulators operating within statutory regulatory schemes [30]. We categorized the largest number of sources under the focus area of regulatory models, governance structures, and reforms (n = 59, 30.3%); followed by registration and licensure (n = 57, 29.2%); nursing roles and standards (n = 53, 27.2%); conduct, complaints, and discipline (n = 13, 6.7%); continuing competence programs (n = 11, 5.7%); and education program approval/accreditation (n = 2, 1%). Through an inductive process, we created subcategories under each of the six key focus areas for a deeper analysis. Fig 7 represents the number of publications in each focus area, inclusive of key subcategories. The detailed characteristics of these focus areas and corresponding subcategories are presented in the following section.

#### Regulatory models, governance structures, and reforms.

We identified 59 sources (30.3%) that discussed, examined, critiqued, or evaluated regulatory models and governance structures, and their reform. We refer to regulatory models as legislative frameworks that define which professional designations are regulated and specify the required structures and processes that facilitate regulation. We define governance structures as the mechanisms by which the nursing regulatory body is controlled and operated, encompassing topics such as the size and composition of councils and committees, the relationship between the board and the organization, and the policies guiding decision-making and accountability. We use the term regulatory governance to refer to the institutions, policies, tools, and processes focused on developing, administering, implementing, enforcing, and reviewing regulation.

The sources in this focus area reveal several overlapping subcategories. Twelve sources (20.3%) identified or discussed factors that influence regulatory governance [3,5,16,46,110,111,116–118,212–214], with two (3.4%) focusing specifically on the influence of the COVID-19 pandemic on nursing regulation [16,111]. Eleven (18.6%) sources included a focus on the mediation of power relationships between nursing regulators and governments, or within and between professions, and their impact on nursing regulation [43,46,47,56,95,102,109,113,115,118,198]. Six (10.2%) sources explored regulatory model reform through legislative changes [55,195–198,216], with four (6.8%) focusing specifically on the regulation of new nursing designations, a process mediated through legislative change [67,85,86,206]. Six (10.2%) sources examined the implementation or evolution of regulatory models over time [3,110,116,118,213,214], and nine (15.3%) compared regulatory models internationally [3,21,58,101,111,114,117,172,178].

The most represented subcategory in this focus area is that of governance structure reform, with 19 (32.2%) sources discussing or examining reforms related to board composition and size, board appointment processes, regulatory philosophies, or decision-making processes [5,12,44,45,51–53,56,94,112,115,195,196,198,205,208,210,211,216]. Six (31.6%) of the sources in this subcategory included a focus on the amalgamation of nursing regulatory bodies [5,52,94,112,198,208]. An additional six (31.6%) sources evaluated current regulatory governance structures and processes against specific criteria [55,195–198,216].

#### Registration and licensure.

We categorized 57 (29.2%) sources asfocused on describing or examining the development, implementation, and impact of registration and licensure requirements and processes. Just over half (n = 29, 50.8%) of the sources included in this focus area were related to the registration and licensure of internationally educated nurses (IENs) and their integration into the workforce. Of these sources, 15 (51.7%) described the experiences of IENs as they navigate the registration and licensure process and integrate into the workforce [119–122,125,127,129,130,156,158,180,181,186,187,207], two (6.9%) of which specifically addressed IENs’ experiences with English language testing [122,180]. One source (3.4%) critiqued the issue of IEN underemployment in Canada, also known as “deskilling” [69]. Four sources (13.8%) discussed the development and implementation of methods to assess IEN competence or educational equivalence [65,79,131,159], five (17.2%) identified or discussed reforms to regulatory processes affecting IEN registration and licensure [4,70,76,78,128], and one (3.4%) evaluated the impact of such reforms [157]. Another source (3.4%) compared IEN registration and licensure requirements internationally [82]. Two sources (6.9%) described Canadian IEN demographics and employment data, as well as factors that influence mobility and retention [57,74].

Of the 57 (29.2%) sources categorized in the registration and licensure focus area, 18 (31.6%) focused on entry-to-practice requirements, such as standardized exams and language testing. Seventeen (94.4%) of these sources discussed or critiqued the implementation of the NCLEX-RN entry-to-practice exam in Canada [66,68,71–73,77,80,96–98,100,103,123,124,126,167,217], two (11.1%) of which were published responses to included sources critiquing Canadian nursing regulators’ implementation of the NCLEX-RN exam [96,103]. One (5.5%) source in this subcategory described the development, implementation, and maintenance of English-language testing [81].

Of the total sources in the registration/licensure focus area, three (5.3%) described the general development and implementation of regulatory registration process reforms [75,182,183], two (3.5%) of which provided an impact evaluation of these reforms [182,183]. Two (3.5%) sources provided external evaluations of nursing regulators’ registration and licensure standards and processes against regulatory principles or best practices [199,200]. A further five sources (8.8%) addressed licensure and labour mobility - two (3.5%) described the mobility patterns of nurses and influencing factors [60,154], one (1.8%) outlined the development of an inter-jurisdictional mobility agreement [64], and two (3.5%) described the development of international mobility agreements [61,155].

#### Nursing roles and standards.

We identified 53 sources (27.2%) that described or examined the regulation of diverse nursing roles and activities including the development, implementation, and/or evaluation of standards, policies, and guidelines. Out of the 53 sources in this focus area, 23 (43.4%) addressed the regulation of advanced practice nursing (APN) [31–35,63,92,104,106,146–150,162,169–171,179,201–204]. These sources compared APN roles and regulation standards internationally [63,92,106,146,149,150,203], examined the characteristics of the APN role as well as the barriers and facilitators influencing its integration [31–33,35,104,147,148,162,169–171,179,201,203,204], and discussed the implementation of specific regulatory processes supporting APN practice [34,202]. A recurring topic in the APN-focused literature was the need for regulatory harmonization across jurisdictions to facilitate APN roles both nationally and internationally [32,35,92,147,170,201–203].

Within this focus area, 20 sources (37.7%) focused on the regulation of nursing activities [83,89,99,107,132–135,137,138,140–142,144,160,161,168,175,177,188]. These sources discussed the development, implementation, and examination of regulations guiding specific nursing activities such as nurse prescribing [83,132] including the prescribing of controlled substances [160,168], the administration of Medical Assistance in Dying (MAiD) [99] and medication abortion [134], principle-based medication administration [89], sexual health care delivery [133], compassionate care provision [142], self-employed nursing [137], professional use of social media [177], genomics-informed nursing practice [135], and the right to refuse unsafe work assignments [107].

Of the total in this focus area, seven sources (13.2%) compared the content of regulatory standards, policies, and guidelines across nursing and interprofessional contexts and jurisdictions in Canada [138,140,141,144,161,175,188]. These comparisons covered the topics of medical cannabis [161], community health nursing [138], environmental health [140], professional collaboration [141], and MAiD [175]. One source (1.9%) examined financial conflicts of interest across four regulated professions in one province [144], and another (1.9%) compared practice expectations between different nursing designations [188].

Seven sources (13.2%) in this overarching focus area described the process of developing regulatory documents, including standards of practice, codes of ethics, and entry-level competencies [87,88,90,91,139,143,174]. Two of these sources (3.8%) focused on the evolution of practice standards or codes of ethics over time [90,143], one (1.9%) discussed the challenges associated with the dynamic scope of nursing practice [84], and two (3.8%) sources explored how regulators can support safe nursing practice [49,184].

#### Conduct, complaints and discipline.

We identified 13 sources (6.7%) that described topics related to professional conduct and/or the development, implementation, and evaluation of complaints, investigations, and disciplinary programs [41,105,108,145,153,164–166,185,192–194,215]. Five sources (38.5%) in this focus area discussed specific topics related to professional conduct, including how addiction discourses shape disciplinary approaches [145], mandatory reporting of colleagues [108], social media use [41,215], and the impact of excessive work hours [166]. One source (8%) analyzed investigation and disciplinary data over time [165]. Three sources (23.1%) described the development of complaints, investigations, and disciplinary processes [105,164,185], one (7.7%) of which also evaluated the impact of implemented reforms [105]. Four sources (30.8%) evaluated complaints, investigations, and disciplinary programs against specific criteria [153,192–194].

#### Continuing competence programs.

We identified 11 (5.6%) sources that related to the development, implementation, and evaluation of continuing competence programs (CCP) (also commonly referred to as quality assurance programs in some jurisdictions) [36–40,93,151,152,163,190,191]. Among the sources in this focus area, five (45.5%) described the process of developing or revising a CCP and its components [36–38,40,163], two (18.2%) examined registrants’ experiences or perspectives on CCPs [151,190], and one (9.1%) evaluated a CCP against regulatory principles or best practice guidelines [191]. Additionally, three sources (27.3%) critiqued specific components of CCPs, including the mandatory practice hour requirement [93], the use of reflective practice exercises to evaluate competence [39], and the exclusion of cardiopulmonary resuscitation (CPR) certification from the CCP requirements [152].

#### Education program approval/accreditation.

We identified two (1%) sources that focused on the regulatory process of approving or accrediting nursing education programs [42,176]. Both sources discussed key recommendations for harmonizing education program approval standards across jurisdictions, one specific to LPNs [176] and the other to NPs [42].

## Discussion

The nature, extent, and range of available scholarship related to nursing regulation in Canada is diverse, covering topics related to regulatory models, governance structures, and core regulatory functions. However, the amount of empirical literature with nursing regulatory issues as the main area of focus is limited, indicating a lack of evidence to support decision-making. There is some evidence that existing scholarship aligns with health practitioner regulation trends; however, several knowledge gaps exist and require further research. In the discussion below, we examine the implications of our findings with a particular focus on a) the current gaps in empirical evidence, b) the need for collaborative approaches to research, and c) future areas for regulatory research.

### Current gaps in empirical evidence

The findings of this review highlight a significant gap in empirical evidence available to inform regulatory decision-making. Despite identifying 195 relevant sources, fewer than half (n = 80, 41%) were peer-reviewed empirical studies. Over half (n = 50, 62.5%) of these empirical studies examined nursing regulation within a broader regulatory, professional, or health context, providing limited in-depth analysis or discussion about the implications for nursing regulation. Only 30 (37.5%) of the included empirical studies situated Canadian nursing regulation as the main area of inquiry [16,105,108,115,116,123,128,133,136–139,141–143,145,151–153,155,157,160,161,163–165,168,174–176]. Although some Canadian nursing regulators actively commission reviews, evaluations, and audits to guide decision-making [191–200,216], the lack of empirical evidence illustrates a significant evidence gap within this context.

Where empirical studies exist, most employed qualitative (n = 45) or mixed methods approaches (n = 17), while quantitative research (n = 7) was notably underrepresented. It is not surprising that most of the peer-reviewed empirical studies utilized qualitative methods since the research questions were mainly exploratory. While qualitative research can also be used for explanatory purposes, the exploratory focus in the extant literature may be a result of nursing regulation being a relatively understudied area of study in the Canadian context. Qualitative research has been identified by scholars as being critical to generating rich data on the development and implementation of regulatory policies and practices, and for understanding the perspectives of diverse system partners [218].

Amongst the included empirical studies in our review, authors of qualitative studies where Canadian nursing regulation was the main area of inquiry used methods such as qualitative description, interpretive description, biographical narrative approaches, comparative case studies, and participatory action research to generate rich, nuanced, and in-depth insights into the knowledge, beliefs, experiences, discourses, and behaviours of regulators, regulated professionals, and system partners (see S4 Appendix: Data Extraction from Scholarly and Grey Literature Sources). Qualitative studies using methods such as policy analysis, document analysis, historical reviews, and critical discourse analysis were also evident and explored the meaning of regulatory policies and approaches in shifting political, social, and economic contexts. The prevalence of qualitative research highlights researchers’ recognition of the essential role it plays in enabling the examination of diverse regulatory contexts and perspectives and providing in-depth insights into regulatory structures, processes, and outcomes.

Five published empirical studies used quantitative approaches where nursing regulation was the main area of inquiry. One study used a retrospective design to explore discipline decision trends over time. Another used a comparative analytic method to examine pass rates before and after the introduction of the NCLEX-RN exam in Canada, comparing results with U.S. nurses. A third study used quantitative measures to develop competencies. A fourth conducted a cross-sectional survey to assess employer perspectives on collaborating with regulators to monitor nurses practicing with conditions or restrictions. Lastly, one study measured the impact of a jurisprudence module using a pre-and post-test design. While quantitative approaches are used to answer a range of exploratory and explanatory questions, there is a notable absence of correlational research that examines the relationships between regulatory policies and practices and patient safety, workforce, and health system outcomes. Additional research might explore, for example, the correlations between discipline approaches and rates of recidivism, the relationships between practice hour requirements and regulated professionals’ competency, or the impact of licensure reforms on the integration and mobility of nurses, to name a few. Explanatory research questions are needed to identify the impact and outcomes of regulation to inform evidence-based regulatory decision-making.

Mixed-methods research draws on the strengths of qualitative and quantitative methodologies and is well-suited to address complex research questions within a dynamic professional regulatory context. Of the published empirical studies that employed mixed-methods approaches where nursing regulation was the main area of inquiry, mixed-methods surveys and surveys combined with interviews or document reviews were most widely used. One study employed a multiple case study to explore the feasibility of inter-jurisdictional mobility. Of particular note is that almost all but one of the published mixed-methods studies were led by regulators evaluating their regulatory policies and programs, demonstrating the value of diverse methods in generating knowledge to inform regulatory decision-making.

Our review reveals a significant increase in the body of literature related to Canadian nursing regulation since 2010, suggesting that this is a growing field of study. The COVID-19 pandemic and the workforce pressures across Canada have further heightened interest in regulatory reform. As regulatory processes continue to evolve in response to emerging risks and challenges, greater methodological diversity can support inquiry into complex regulatory questions and issues and inform improvements to regulatory structures, processes, and outcomes.

### The need for collaborative approaches to research

While the literature has grown steadily since 1995, the limited scholarly literature exploring Canadian nursing regulation as the main area of focus is not surprising, as the number of scholars in this field is relatively small. Our review identified that 33.8% of the sources were produced, written, or commissioned by individuals affiliated with a regulatory body. This also presents opportunities for researchers and regulatory leaders to work collaboratively to identify and co-create shared research goals. This is particularly important as evidence illustrates a disconnect between what is researched and what regulators identify as priorities [17].  For example, the NCLEX-RN entry-to-practice exam sparked controversy within the profession, generating significant attention from non-regulator scholars [66,68,71–73,77,80,97,98,100,123,124,126,167,217], while advanced practice nursing regulation has been driven by advocacy efforts led by non-regulator researchers aiming to reduce barriers to practice [31–35,63,92,104,106,146–150,162,169–171,179,202–204]. In contrast, publications led by, commissioned by, or involving regulators typically focus specifically on regulatory standards, processes, and reforms [34,37,49,51,56,61,64,65,75,76,87–90,142,157,163,164,168,174,176,177], reflecting the topics that nursing regulators likely find more relevant in guiding their work. Given these findings, our team is actively working with system partners to co-create pan-Canadian nursing regulation research priorities and to identify the infrastructure needed to sustain long-term collaborative knowledge development and mobilization [219].

Nearly half of the scholarly sources (n = 87, 44.6%) discuss nursing regulation within a broader regulatory or professional context. While potentially impactful, these sources often address regulatory themes alongside other findings, creating barriers to the identification or accessibility of findings that may be used to inform regulatory decision-making. For example, despite significant changes to registration processes from 2007 to 2018, there is little reported change in the experiences of IENs during this period [119–122,125,127,129,156,158,181,186,187,207]. This may be due to ineffective dissemination of relevant findings to nursing regulators, poor keyword selection, or results lacking specificity and correlation to current registration and licensure processes. This underscores the need for authors to use regulation-specific keywords, develop more effective knowledge translation strategies, and conduct more focused, detailed evaluations of regulatory processes and reforms. The lack of a common taxonomy used in regulatory research has been identified as a key issue in the World Health Organization’s (WHO) recent guidance on the design, reform, and implementation of health practitioner regulation [30]. One recommendation to generate a more robust evidence base is to standardize the taxonomy and terminology of health practitioner regulation [30].

#### Pan-Canadian approach to nursing regulation research.

The geographic distribution of the literature identifying a specific Canadian province or territory aligns with the population size of the jurisdiction and the number of regulated nurses. The provinces of Ontario (31.7%), British Columbia (26%), and Alberta (11.5%) were the most well-represented in the literature. These provinces have the highest populations [220] and the largest number of regulated nurses [221] respectively, excluding Québec which was not well represented in the English language literature. This correlation may be the result of more regulatory developments, higher representation of scholars and professional groups in these regions, or better-resourced nursing regulatory bodies that have the capacity to engage in research more frequently. A significant portion of the sources (n = 104, 53.3%) focused on specific provincial or territorial jurisdictions, while 34.4% (n = 67) took a broader national perspective. Given Canada’s federated system, this distribution is expected. There are instances where it is appropriate to confine the inquiry to provincial or territorial contexts due to differences in governance systems, while in other instances, cross-jurisdiction comparisons can yield valuable insights, particularly when aiming for greater harmonization.

Nursing regulation in Canada is complex with four different nursing designations regulated across 22 nursing regulators in 13 different jurisdictions. The federated system has been cited as a significant barrier to regulatory harmonization [35,50,59,147] and labour mobility [4,15,59,60,62,154]. To address these challenges, initiatives such as an inter-jurisdictional mutual recognition project [64] and investments into a national nurse database to enable more consistent data collection and improve licensure processes [222] have been explored. There is evidence of pan-Canadian collaboration, particularly in the development of pan-Canadian entry-level competencies [87,88] and nurse practitioner regulatory frameworks [91,201]. While provincial and territorial regulators will continue to address research questions specific to their jurisdictions, the broader push for harmonization presents a unique opportunity for regulatory leaders and scholars to pursue research initiatives collaboratively. Greater efforts must be made to ensure Québec, a predominantly Francophone province, is included in these collaborations.

#### Interprofessional regulatory research.

Nursing regulation not only operates across geographic boundaries but also intersects with other professions. Despite trends toward umbrella legislation in some Canadian jurisdictions and the amalgamation of regulatory bodies of different professions [5,198,208], there is little literature comparing regulation across professions. Only one source in our review directly compared the regulation of different nursing designations [188] and another compared nursing regulation with other regulated professionals [144]. In some cases, nursing regulation was included in a more general analysis of health practitioner regulation [3,110,198,207]. There are many similarities in the practice of professional regulation that can be explored irrespective of professions within and beyond the health sector. Additionally, regulators are aligning with international standards by commissioning external reviews and adopting best practices from leading regulatory organizations such as the UK’s PSA [193,197] and other regulatory experts [191,194,196,198–200]. Growing awareness of, and alignment with, the broader regulatory context can help generate novel and relevant research questions to strengthen the practice of professional regulation as a whole.

### Future areas for regulatory research

The application of the structures, process, and outcomes framework reveals that outcomes research is significantly lacking. Of the total sources included, only ten (5.1%) explored the outcomes of nursing regulation. Although some nursing regulators have begun developing and publicly reporting on key performance indicators [223] or commissioned voluntary external reviews to evaluate their performance [191,193,194,196,197,199,200], this topic is notably underdeveloped within the literature base and requires further exploration. Given the growing emphasis on performance measurement and accountability [5,12,46,113,115,117,198,205,224] we argue that future research should go beyond comparing or describing structures and processes and prioritize evaluating the impact of regulation to inform improvements.

#### Regulatory models, governance structures, and reforms.

Regulatory models, governance structures, and reforms were the most widely explored in the literature, highlighting growing attention to the ongoing evolution of nursing regulation across the country. The literature reveals a strong focus on reforms aimed at enhancing transparency, accountability, and public protection. The influence of government oversight and legislative reforms on nursing regulation is also a recurring theme, emphasizing the complex and shifting power dynamics between regulatory bodies, governments, and professional organizations.

The emphasis on regulatory reform is not surprising, as good governance is the bedrock of effective regulation. The strong representation of this topic in the literature is likely driven by recent and upcoming legislative [5] and voluntary governance changes across Canadian regulatory jurisdictions, reflecting the broader trend of regulatory modernization in Canada [12,205]. Examples of key reforms include the divestment of activities such as professional advocacy which is now commonly understood to fall within the purview of professional associations, and the shift toward single-mandate nursing regulators focused squarely on regulatory functions. Discussions and critiques about these policy trends have shown up in our review, which signals some alignment between regulatory scholarship and practice [5,50,95,115,205]. Similarly, the shift away from self-regulation to co-regulation in some jurisdictions is reflected predominantly in the grey literature that describes changing governance structures such as greater government oversight, and the introduction of smaller, competency-based boards and committees [51,56,195,196,198,205,210] with equal representation of public and professional members [5,12,56].

Another recent and drastic shift in regulatory governance is the amalgamation of regulatory bodies within and across professions to increase efficiency which is represented in the literature within our review [5,12,16,52,112,198,208,209]. Concerns about transparency, accountability, and fairness are key reasons for the strong attention to regulatory modernization and reform across Canada. These concerns are reflected in the literature as evidenced by sources discussing regulatory accountability and oversight [5,12,46,113,115,117,198] and regulatory philosophies such as principle-based, right-touch, risk-based, or relational regulation [16,51,53,56,196,205,208]. While the heavy focus in this area aligns with the current regulatory landscape in Canada and internationally, the literature that exists is largely descriptive. Future studies should examine the outcomes and impact of regulatory reforms. For example, research questions might include: What are the impacts of amalgamating regulatory bodies on patient, workforce, and health system outcomes? How are regulators measuring and evaluating their performance? How can performance measurement frameworks be standardized nationally and globally?

#### Registration, licensure, and labour mobility.

Registration, licensure, and labour mobility are central to the growth and mobilization of the nursing workforce. The increasing globalization of the nursing workforce is reflected in a growing number of scholarly sources (n = 16, 8.2%) published since 2010 that compare nursing regulation internationally, accompanied by calls for global harmonization as an enabler of labour mobility [21,58,60,63,82,92,101,106,132,146,149,150,172,173,178,203]. Only two sources (1%) included in our review discussed the formation of international mutual recognition agreements [61,155], and further research is needed to assess the effectiveness of existing regulatory frameworks in facilitating international labour mobility.

Over half of the sources in this focus area discussed IENs’ experiences navigating licensure, highlighting issues related to language testing, competency assessments, and other regulatory barriers that have created significant challenges in entering the Canadian nursing workforce. The strong emphasis on licensure and labour mobility within the literature throughout the decades aligns with growing concerns about nursing workforce shortages, which have worsened since the COVID-19 pandemic [225–230]. Ensuring efficient, ethical, and equitable licensure processes is crucial for integrating qualified nurses into practice without unnecessary delays. However, despite decades of research on improving licensure processes, the persistence of regulatory barriers suggests that research findings are not always translating into effective policy improvements. IEN licensure reforms have been implemented rapidly since the pandemic to address the worsening workforce shortage, and future research should examine questions such as: What are the implications of IEN licensure reforms on the nursing workforce, system partners, and patient safety? What is the impact of licensure reforms on domestic labour mobility? The use of quantitative regulatory data can be particularly useful in understanding changes to the supply of regulated nurses across the country and the movement of nurses nationally and globally.

#### Nursing roles and standards.

The strong emphasis on the regulation of APNs and the development of regulatory standards or guidance for new practice areas reflects the need for responsive, forward-thinking regulation that empowers nurses to meet the growing demands of shifting population health needs. However, a key theme in this literature is the variability in regulatory standards across jurisdictions, which contributes to inconsistent practices and ultimately, variability in the public’s access to health services depending on geographical location. Greater efforts are required to explore strategies to increase consistency across jurisdictions as nursing roles continue to evolve. Future research projects might explore questions such as: How can the scope of practice of nursing designations be made more consistent across the country? What risks and opportunities exist in regulating new and emerging areas of practice? How can regulators mitigate these risks while enabling innovation amongst healthcare professionals?

#### Conduct, complaints, and discipline.

While addressing professional misconduct is a core function of professional regulators, our findings suggest that this has not been a strong area of inquiry. However, the extant literature does highlight critical issues such as the impact of social media use on professional conduct, mandatory reporting obligations, and the effectiveness of disciplinary procedures. This is an area that requires particular attention, as the approach used to address professional misconduct can have significant consequences on regulated professionals and the parties involved. For example, research from other international jurisdictions has found that regulated professionals involved in disciplinary processes have a higher risk of mental health issues and self-harm [231,232]. Some scholars have begun exploring the concept of compassionate regulation within the context of professional conduct processes [233,234], which may be worth further exploration and evaluation in the Canadian nursing regulatory context.

Another area of research that we have identified in the international literature that is not well represented in our review is related to bias, discrimination, and racism within the context of complaints, investigation, and disciplinary processes [192,235–246]. Opportunities exist to examine how these issues manifest in regulatory policies and processes, and what strategies can be implemented to address them. Greater research is also needed to examine the outcomes of disciplinary approaches. For example, future research might include quantitative comparative studies using regulatory data to explore the impacts of different disciplinary approaches (e.g., restorative justice, trauma-informed) on outcomes such as recidivism. Given the lack of regulatory research that explores the perspectives of patients and regulated professionals, another question that researchers can explore using qualitative approaches is: What are the experiences of complainants, witnesses, and regulated professionals who are subject to a complaint and how can complaints and professional conduct processes be improved to balance support, fairness, compassion, and safety?

#### Continuing competence programs.

Nursing regulators across Canada are required by legislation to implement and enforce CCPs as a proactive measure to support continuous learning. Significant time and resources are dedicated to this core regulatory function; however, inquiry into the development and outcomes of CCP has received comparatively little attention, comprising just 5.6% (n = 11) of the included sources in our review. Although mandatory continuing professional development is an approach that is used widely by professional regulators globally, existing evidence about the design and outcomes of CCPs is mixed [18,247–251]. Research that attends to provincial and territorial contexts may be particularly useful for informing regulators about the design and effectiveness of their CCPs. Examples of research questions include: What methods can best assess nurses’ competency throughout their career span? What are the impacts of mandatory continuing competence programs on nurses’ competency? How do the approaches employed by Canadian nursing regulators compare with other professional regulators and global jurisdictions?

#### Education program approval/accreditation.

Education program approval and accreditation represent the least-studied regulatory function, with only 1% (n = 2) of sources addressing this area. The limited available literature included in the review discussed the harmonization of approval standards for nursing programs, particularly for LPNs and NPs. Nursing education programs are continuously required to update curricula to ensure graduates are well-prepared to function within evolving healthcare landscapes. As a result, a key research question that should be explored is: What strategies enable regulators to remain agile and responsive to shifting population health needs, innovative technologies, and emerging risks to ensure regulatory standards support high-quality nursing education that prepares safe, competent, and ethical nurses entering the workforce?

#### Emerging regulatory research trends.

Diversity, equity, inclusion, anti-racism, and decolonization are growing regulatory priorities. In Canada, recent legislative changes in British Columbia require health practitioner regulators to implement specific anti-discrimination and support measures [5,12], and the included sources speaking to anti-discrimination, anti-racism, or diversity, equity, and inclusion topics focus on this province exclusively [5,12,192,216]. While this topic is not well represented in the extant literature base, it is indeed gaining attention within health practitioner regulation globally [236,239,241,252–257]. Future research should explore how health practitioner regulators perpetuate institutional and interpersonal racism through regulatory policies and practices, and the actions they can take to create anti-racist regulatory and healthcare systems. Solutions must be evidence-informed, and some members of our team are conducting a review exploring the nature, extent and range of scholarship on this topic [258].

The adoption of technology, specifically artificial intelligence, in regulation is another emerging trend [20,205,216], but this review revealed limited literature on how Canadian nursing regulators are integrating technology into regulatory processes or setting standards for its use by regulated nurses. Where sources discuss technology, they focus on the appropriate use of social media by regulated professionals [41,215] and regulators [205], the implementation of virtual care [64], and the use of technology to support regulatory functions [40,163]. There is, however, emerging scholarship exploring the implications of technology and health practitioner regulation globally [259] and in the Canadian context [260]. Given the significant impacts that technological advances in regulatory and professional practice can have on public safety and regulated professionals, research must keep pace with this evolving technological landscape to ensure the ongoing protection of the public. Future research can explore questions such as: What are the risks and opportunities for integrating technology into regulatory processes and how do these compare across professions and jurisdictions globally? What are the emerging risks of technology on patient safety and how can regulators balance safety without creating barriers to innovation?

## Implications

Canadian nursing regulation is a growing field of study and, as such, significant gaps remain in the existing literature. Much of the current literature base consists of discussion papers and commentaries. While this literature allows for the exploration and critique of regulatory issues and solutions, empirical research is required to develop knowledge through structured observation and experimentation to advance the science of professional regulation. Where empirical studies exist, the extant literature is mainly exploratory and descriptive. As this area of scholarship continues to expand, greater attention to explanatory research using diverse methodologies and methods can help to explore the impacts and outcomes of regulatory approaches, policies, and practices on patient safety, the health workforce, and health system outcomes.

A key challenge identified in this review is the lack of harmonization of nursing regulation given Canada’s federated system. A collaborative approach between regulators and researchers to co-create research agendas, set data collection priorities, and develop knowledge mobilization strategies can drive meaningful reforms. The gaps identified in this review provide both regulatory scholars and regulators with a foundation to create a shared research agenda that not only advances the science of regulation but can be applied in practice. Further, professional regulators are custodians of rich data that can be useful for regulatory decision-making and broader health workforce planning; however, data collection is often inconsistent across regulators, creating difficulties for comparative research across regulators, professions, and jurisdictions. Legislative barriers also exist, which impact what data regulators can collect and how it is shared. These important areas should be further explored and can be supported through existing pan-Canadian collaboratives such as the Canadian Council of Registered Nurse Regulators, the Canadian Council of Practical Nurse Regulators, the Registered Psychiatric Nurse Regulators of Canada, and the Canadian Nurse Regulator Collaborative. In addition, efforts are required to develop infrastructure to support continuous knowledge development and mobilization, and members of our team are working on developing a regulatory research hub to support these efforts [219].

Lastly, Canadian nursing regulation does not function in isolation. The increasing globalization of healthcare and the growing mobility of the health workforce necessitate a broader research perspective. Given these trends, greater emphasis should be placed on conducting interprofessional and inter-jurisdictional research to strengthen the professional regulation evidence base within broader national and global healthcare ecosystems.

## Limitations

While this scoping review provides valuable insights into the emerging field of Canadian nursing regulation, several limitations should be noted. First, the exclusion of French-language literature limits insights into nursing regulation in the province of Québec. Second, the rapid growth of the field means that new studies may have emerged since the completion of the review, potentially impacting the comprehensiveness of the findings. Given the consistent updates on regulators’ websites, there may be grey literature that was not captured in this review. Third, legal or social science literature that includes discussion on nursing regulation without the use of keywords related to nurses or nursing may not be captured. Fourth, although we attempted to categorize and analyze the literature by nursing designation, this was challenging due to the lack of clarity around the terms used and the variability of nursing designations across jurisdictions both nationally and internationally. For example, IENs were a key target population in the literature but this general term can refer to nurses of any designation. Likewise, the terms nurse practitioner and clinical nurse specialist (which is not a protected title in any Canadian jurisdiction) were sometimes used in isolation or grouped under the umbrella of APNs. Many sources referred to multiple nursing designations or used the term nurse more broadly. As a result, accurately describing the nature, extent, and range of the literature according to nursing designation was not possible. However, this represents an important finding, and we encourage scholars to be cognizant of defining the terms used to describe the categories of nurses to enable more meaningful disaggregated analysis. Despite these limitations, the existing body of work described in this review provides a meaningful foundation to advance efforts to strengthen evidence-informed nursing regulation.

## Conclusion

Our review provides a comprehensive overview of the nature, extent, and range of scholarship informing nursing regulation in Canada. The 195 sources identified in the review cover a broad spectrum of regulatory topics related to regulatory governance, structures and reforms; registration and licensure; nursing roles and standards; conduct, complaints, and discipline; continuing competence programs; and education program approval/accreditation. The review highlights how the existing scholarship aligns with emerging trends in health practitioner regulation, and the significant knowledge gaps that remain. Priority should be placed on addressing these gaps, including the need for more outcomes-focused explanatory research and strategic knowledge mobilization partnerships, as well as the development of more standardized regulatory performance measurement and evaluation frameworks. Addressing these gaps will require collaboration and co-creation between regulators, scholars, employers, governments, educators, and members of the public to build a more robust evidence base capable of informing regulatory decision-making.

## Supporting information

S1 Appendix Inclusion/Exclusion Screening Form. (PDF)

S2 Appendix Database Searches. (PDF)

S3 Appendix PRISMA-ScR Checklist. (PDF)

S4 Appendix Data Extraction from Scholarly and Grey Literature. (PDF)

S5 Appendix Sources Excluded Following Full-Text Review. (PDF)
