# Supplementary material for: Nursing regulation in Canada: Insights from a scoping review
Source: PLoS One. 2025 May 16;20(5):e0323716. doi: 10.1371/journal.pone.0323716 (PMC12084052; doi:10.1371/journal.pone.0323716)
Supplement: S4 Appendix — (PDF) [file pone.0323716.s004.pdf]

S4 Appendix: Data Extraction from Scholarly and Grey Literature

S. #	Reference	Year	Type of Publication	Empirical Method	Purpose/Aim	Focus	Jurisdiction	Provinces	Key Findings/Concepts Relevant to Nursing Regulation	Relevance to Nursing Regulation
1	Acorn M. The future of advanced practice nursing: What's next for Canada and the world? Nurs Leadersh. 2021;34(4):79–85.	2021	Commentary	N/A	Discusses the need for global clarity and consistency in advanced practice nurses' (APN) role definition, education, regulation, scope of practice, working conditions and integration to inform the design, delivery and leadership aspiring for health, education and socio-economic systems.	Nursing Roles/Standards	Global	N/A	Key strategic actions targeting the influence and impact of APNs include changing curriculum to emphasize role optimization, credentialing and title protection, and raising public and professional awareness of the role.	Canadian nursing regulation is the focus
2	Adams TL. Regulating professions in Canada: Interprovincial differences across five provinces. Journal of Canadian Studies. 2009;43(3):194–221.	2009	Empirical study	Qualitative (historical review)	Explores the regulation of professions in five Canadian provinces (British Columbia, Saskatchewan, Ontario, Quebec and Nova Scotia) from confederation to 1961 and examines how professional regulation has varied across locale.	Regulatory Models, Governance Structures, and Reforms	Provincial/ territorial	Multiple (BC, SK, ON, QC, NS)	There is significant variation in which professions are regulated and when and how they were regulated due to factors such as occupational growth, professional organization, interprofessional conflict, and population density.	Canadian nursing regulation is included in the broader context
3	Adams TL. Health professional regulation in historical context: Canada, the USA and the UK (19th century to present). Hum Resour Health. 2020;18(1):72.	2020	Empirical study	Qualitative (historical review)	Explores the regulation of healthcare professionals in Canada, the United States and the United Kingdom, from the mid-nineteenth century to present and considers how regulation has varied across time and locale. Major concerns, debates, and stakeholders shaping regulatory outcomes are discussed.	Regulatory Models, Governance Structures, and Reforms	Global	N/A	Self-regulated professions emerged in the nineteenth century. Various stakeholders influenced the regulation of professions based on their response to concerns about healthcare quality, access and professional training. Changing stakeholders and interests shaped regulatory practices over the twentieth and twenty-first centuries.	Canadian nursing regulation is included in the broader context
4	Adams TL. Amalgamation of professional regulators: Conflicting perceptions and beliefs among canadian regulatory leaders. J Nurse Regul. 2022;13(2):25–33.	2022	Empirical study	Qualitative (Qualitative description)	Explores Canadian regulatory leaders' views about professional regulator amalgamation.	Regulatory Models, Governance Structures, and Reforms	Provincial/ territorial	Multiple (BC, AB, ON, SK, NL, NS)	There is limited evidence and conflicting views on amalgamation of professional regulators (regulatory change) and caution is needed among those pursuing amalgamation of professional regulators. Collaboration is key to successful amalgamation and should not be forced or mandated.	Canadian nursing regulation is the focus
5	Adams TL. Drivers of regulatory reform in Canadian health professions: Institutional isomorphism in a shifting social context. Journal of Professions and Organization. 2022;9(3):318–32.	2022	Empirical study	Qualitative (interviews)	Explores the drivers of change in health professions regulation in Canada and the role of institutional isomorphism in shaping regulatory reform.	Regulatory Models, Governance Structures, and Reforms	Provincial/ territorial	Multiple (BC, AB, ON, SK, NL, NS)	Examples of recent regulatory reforms in health profession regulation in six provinces are highlighted (e.g. amalgamation of nursing regulators in several Canadian provinces). A wide range of stakeholders and social trends has driven regulatory change internationally. External and coercive drivers, influenced by the agendas of politicians and state actors in response to a scandals, media reports and public concerns have been particularly influential.	Canadian nursing regulation is included in the broader context
6	Adams TL, Wannamaker K. Professional regulation, profession-state relations and the pandemic response: Australia, Canada, and the UK compared. Social Science & Medicine. 2022;296:114808.	2022	Empirical study	Qualitative (policy analysis)	Explores how systems of healthcare professional regulation impacted regulatory responses during the first wave of the COVID 19 pandemic. Explores whether the presence of national-level health professions regulators in the United Kingdom and Australia lead to faster and more coordinated pandemic responses than in Canada.	Regulatory Models, Governance Structures, and Reforms	Global	N/A	Summarizes professional regulation changes by professional group (including nursing) based on region. There is little evidence to suggest that one regualtory system is better than another in facilitating responses during crises, however regulatory structure shape the nature of regulatory policy change.	Canadian nursing regulation is included in the broader context
7	Almost J. Regulated nursing in Canada: The landscape in 2021 [Internet]. Canadian Nurses Association; 2021 [cited 2024 May 7]. Available from: https://www.cna-aiic.ca/en/nursing/regulated-nursing-in-canada	2021	Grey literature	Research study report	Outlines the similarities, differences, and practices of the various designations of nurses in Canada including licensed practical nurses, registered psychiatric nurses, registered nurses, and nurse practitioners.	Regulatory Models, Governance Structures, and Reforms	National	N/A	Describes the history, structures, regulatory requirements, roles, education, and scopes of practice of the four regulated designations of nurses in Canada, as well as touching on advanced practice nursing, specialty nursing practice and nurse midwives.	Canadian nursing regulation is the focus
8	Anderson J, Puckrin K. Social network use: A test of self-regulation. J Nurse Regul. 2011;2(1):36–41.	2011	Empirical study	Surveys (*no details of quantitative or qualitative)	Explores the benefits and risks of using social networks and make recommendations for nursing regulators.	Nursing Roles/Standards	National	N/A	Recommends nurse regulators raise registrants' awareness of the power and permanence of social networks, promote the use of practice standards, and lead the development of appropriate social media tools for use by nursing professionals.	Canadian nursing regulation is included in the broader context
9	Baldwin-Bojarski SKR. Proving my competency one test at a time: Internationally educated nurses and the Canadian English Language Benchmark Assessment for Nurses [Internet] [M.Ed.]. [Ontario, CA]: Queen's University; 2016 [cited 2024 May 1]. Available from: https://www.proquest.com/docview/1928901796/abstract/3AEF664C830E40DEPQ/1	2016	Thesis/ Dissertation	Qualitative	Examines the experiences of Canadian English Language Benchmark Assessment for Nurses (CELBAN) test-takers who completed the exam in Ontario in the winter of 2015.	Registration/ Licensure	Provincial/ territorial	ON	Recommendations for regulators include: clear communication of licensure requirements, monitoring of testing locations and dates, provision of test preparatory materials, suggestions for minor changes to testing administration protocols, and call for evaluation of test validity through continued research.	Canadian nursing regulation is the focus
10	Balneaves LG, Alraja AA. "Guarding their practice": A descriptive study of Canadian nursing policies and education related to medical cannabis. BMC Nurs. 2019;18(1):66.	2019	Empirical study	Mixed methods (Multi-phase descriptive study including a review of documents, interviews and online surveys)	Summarizes nursing policies in Canada related to medical cannabis; explores the perspective of nursing regulatory bodies regarding practice and policy issues related to medical cannabis; and examines the inclusion of medical cannabis content within Canadian nurse practitioner (NP) curricula.	Nursing Roles/Standards	National	N/A	Of the 12 nursing regulatory bodies in Canada, only 7 had policies or statements related to cannabis, with only Ontario allowing NPs to authorize medical cannabis. Barriers include a inconsistent and inadequate provider education, and a lack harmonized regulations, policies, and clinical guidelines.	Canadian nursing regulation is the focus

11	Bassendowski S, Petrucka P. Perceptions of select registered nurses of the Continuing Competence Program of the Saskatchewan Registered Nurses' Association. J Contin Educ Nurs. 2009;40(12):553–9.	2009	Empirical study	Mixed methods (Survey)	Explores the perceptions that select registered nurses have about the Continuing Competency Program (CCP) in Saskatchewan and how their view of the degree of professional control (as assessed through locus of control) that they have affects their perspective about the outcomes of the program.	Continuing competence program	Provincial/ territorial	SK	Self-reflective tools enable registered nurses to rate their achievement of professional foundational competencies and the majority of participants indicated beliefs showing an internal locus of control and agreement regarding the role of the nursing profession in the advancement of the discipline through standards and professional development.	Canadian nursing regulation is the focus
12	Baumann A, Norman P, Blythe J, Kratina S, Deber R. Accountability: The challenge for medical and nursing regulators. hcpol. 2014;10(SP):121–31.	2014	Empirical study	Qualitative (exploratory descriptive - interviews)	Explores how leaders of regulatory authorities perceive and demonstrate accountability to their stakeholders.	Regulatory Models, Governance Structures, and Reforms	National	N/A	Key findings include variability in the perception, definition, and demonstration of accountability across regulators but all participants reported a responsibility to be accountable to government, public, and registrants.	Canadian nursing regulation is included in the broader context
13	Baxter CE. Philippine nurse migration to the Canadian prairies: Challenges and opportunities [Internet] [Ph.D.]. [Scotland]: Queen Margaret University; 2017 [cited 2024 May 1]. Available from: https://www.proquest.com/docview/2189046950/FAA44F97C528426EPQ/1?sourcetype=Dissertations%20&%20Theses	2017	Thesis/ Dissertation	Mixed methods (interviews and online surveys)	Explores and describes the integration of Registered Nurses (RNs) from the Philippines into the nursing workforce in the Canadian Prairies.	Registration/ Licensure	Provincial/ territorial	Multiple (Prairie Provinces)	Participants identified four main reasons for migrating to Canada and the Prairie Provinces. These included the presence of family in Canada, the demand for nurses in the Prairie Provinces, ease of migration, and the opportunity for a better life. Upon entry into Canada, IENs encountered challenges in both the pre-licensure and post-employment phases. Financial constraints, obtaining the necessary English language proficiency levels, and meeting the RN licensure requirements were the key pre-licensure challenges identified.	Canadian nursing regulation is included in the broader context
14	Bayne L. Underlying philosophies and trends affecting professional regulation [Internet]. College of Registered Nurses of British Columbia; 2012 [cited 2024 May 8]. Available from: https://www.nsrhpn.ca/wp-content/uploads/2014/08/philosophies-and-trends-affecting-regulation-2012.pdf	2012	Grey literature	Research study report	Presents an overview of perspectives collated from a literature review and informant interviews with the aim of facilitating the formation of a regulatory philosophy and framework for the nursing regulator in British Columbia (CRNBC).	Regulatory Models, Governance Structures, and Reforms	Provincial/ territorial	BC	Presents views on the major trends that will affect the context in which CRNBC works, factors that may influence what the organization will be required to attend to, and views on how CRNBC might conduct its work under these circumstances.	Canadian nursing regulation is the focus
15	Beardwood B. The loosening of professional boundaries and restructuring: The implications for nursing and medicine in Ontario, Canada. Law & Policy. 1999;21(3):315–43.	1999	Discussion paper	N/A	Examines the effects of the Regulated Health Professions Act and the related redistribution of power in the health care sector and its impact nurses' and physicians' political, clinical, and economic autonomy.	Regulatory Models, Governance Structures, and Reforms	Provincial/ territorial	ON	Suggests the combination of health care restructuring and expanded legislation redefining regulated health professions has reduced medical dominance and increased managerial dominance of healthcare professionals in Ontario, Canada.	Canadian nursing regulation is included in the broader context
16	Beardwood BA, French SE. Mediating complaints against nurses: A consumer-oriented educational approach. Can J Nurs Res. 2004;36(1):122–41.	2004	Empirical study	Qualitative (participatory evaluative method - interviews and focus groups)	Assesses the effectiveness and impact of the regulatory Participative Resolution Program (PRP) by reporting on interviews with complainants, registrants, the College of Nurses of Ontario (CNO) investigators and practice consultants, and key informants.	Conduct/Complaints /Discipline	Provincial/ territorial	ON	Outlines the impact of the PRP on the registrant, complainant, and the workplace. Results demonstrate the PCP meets many of the goals of the CNO but the process is complex and its effectiveness is limited by a lack of power to enforce system change in the interest of safe professional practice.	Canadian nursing regulation is the focus
17	Beardwood BA, Kainer JM. Exploring risk in professional nursing practice: An analysis of work refusal and professional risk. Nursing Inquiry. 2015;22(1):50–63.	2015	Empirical study	Qualitative (interviews)	Explores perspectives of professional risk by analyzing three professional nursing bodies' (CNO, RNAO, ONA) views of professional codes governing the right of nurses to refuse unsafe work assignments.	Nursing Roles/Standards	Provincial/ territorial	ON	All three organizations acknowledged that patient safety must be protected and that employers have an obligation to mitigate risk by ensuring a safe work environment for nurses but there was a discrepancy over the degree of perceived professional accountability of the individual nurse to manage risk in the context of their responsibility to client care.	Canadian nursing regulation is included in the broader context
18	Beaton M, Walsh J. Overseas recruitment: Experiences of nurses immigrating to Newfoundland and Labrador, 1949-2004. Nursing Inquiry. 2010;17(2):173–83.	2010	Empirical study	Qualitative (Oral history method)	Documents the experiences and challenges of nurses who were recruited from overseas to immigrate to Newfoundland and Labrador between 1949-2004.	Registration/ Licensure	Provincial/ territorial	NL	One of the thematic findings relates to the process of immigration and nursing licensure. The historical account notes that the licensure process varied significantly between participants, reflective of the variance in individual international education programs and the changing standards for professional licensure throughout the studied period of time.	Canadian nursing regulation is included in the broader context
19	Bellaguarda MLDR, Nelson S, Padilha MI, Caravaca-Morera JA. Prescriptive authority and nursing: A comparative analysis of Brazil and Canada. Rev Latino-Am Enfermagem. 2015;23(6):1065–73.	2015	Empirical study	Qualitative (Comparative study)	Compares the practice of nurse prescribing in Brazil and Ontario, Canada.	Nursing Roles/Standards	Global	N/A	Nurse prescribing practices in Brazil and Ontario, Canada are described and both jurisdictions face barriers related to professional education and competence, credibility, and acceptance by the public and other health care professionals.	Canadian nursing regulation is included in the broader context
20	Black J, Allen D, Prendergast P, Belcourt T, Brown S, Boudreau P, et al. An evaluation framework for RN competencies: A jurisdictional collaborative process. J Nurse Regul. 2014;5(1):52–6.	2014	Discussion paper	N/A	Describes the work of a jurisdictional collaborative process work group through the Canadian Council of Registered Nurse Regulators (CCRNR) in reviewing and revising entry-level registered nurse competencies in 2011 and 2012, including the development of an evaluation framework.	Nursing Roles/Standards	National	N/A	The working group found the structured collaboration framework was successful and may be transferrable to other regulatory organization collaborations.	Canadian nursing regulation is the focus
21	Black J, Allen D, Redfern L, Muzio L, Rushowick B, Balaski B, et al. Competencies in the context of entry-level registered nurse practice: A collaborative project in Canada. Int Nurs Rev. 2008;55(2):171–8.	2008	Discussion paper	N/A	Describes the process used by professional staff from 10 Canadian jurisdictional regulatory bodies between 2004-2006 to develop harmonized entry-level competencies for registered nurse practice.	Nursing Roles/Standards	National	N/A	The process resulted in a document stating the core competencies for entry-level registered nurses in the 10 participating jurisdictions and includes several components that establish the context in which entry-level competencies are developed and applied.	Canadian nursing regulation is the focus
22	Blumer L, Giblin C, Lemermeyer G, Kwan JA. Wisdom within: Unlocking the potential of big data for nursing regulators. International Nursing Review. 2017;64(1):77–82.	2017	Discussion paper	N/A	Explores the potential for incorporating big data in nursing regulators' decision-making and policy development, with a focus on research conducted through the College and Association of Registered Nurses of Alberta.	Regulatory Models, Governance Structures, and Reforms	Provincial/ territorial	AB	Nursing regulators have access to extensive data which can be utilized in developing relevant, sound, and evidence-based nursing and health policy.	Canadian nursing regulation is the focus

23	Blythe J, Baumann A. Internationally educated nurses: Profiling workforce diversity. International Nursing Review. 2009;56(2):191–7.	2009	Discussion paper	N/A	Provides a profile of nurses educated in different countries who are employed in a major settlement jurisdiction in Ontario, Canada.	Registration/ Licensure	Provincial/ territorial	ON	Findings demonstrate internationally educated nurses (IEN) in Ontario settle mainly in urban areas but otherwise population demographics are diverse in the categories of age, gender, work status, type, and place of employment which limit generalization of this group. Education and regulatory conditions impact the IEN population in Ontario and although the number of IENs entering workforce has increased, attrition of IENs is significant and there are noted variances in retention based on country of origin. International educational and regulatory differences illustrate the need to create global nursing standards and further exploration about differences in workforce profiles should provide insights leading to improved utilization of IENs.	Canadian nursing regulation is included in the broader context
24	Bolongaita LJAC. Modelling the impact of changes in education requirements on nurses' labour market outcomes [Internet] [Ph.D.]. [Ontario, CA]: University of Toronto; 2021 [cited 2024 May 1]. Available from: https://www.proquest.com/docview/2605676477/abstract/C9343DA31109465BPQ/1	2021	Thesis/ Dissertation	Quantitative (multiple using data from the Statistics Canada survey of Labour and Income Dynamics (SLID) administered the SLID from 1993 to 2011)	Investigates the impact of the updated entry to practice requirements for both Registered Nurses (RN) and Licensed Practical Nurses (LPN) in most jurisdictions in Canada on probability of participation in the nursing labour force, wages, and choice of care sector of employment.	Registration/ Licensure	National	N/A	The updated entry-to-practice (ETP) requirements did not significantly impact either designation's probability of participation in the nursing labour workforce, but there was an impact on the probability of non-nursing employment. There was no impact on RN wage, but there was a decrease in LPN wage related to the changes in RN ETP requirements and there was an impact on choice of care sector of employment in both groups. Jurisdictions should consider the labour market impact of changes in ETP requirement on targeted as well as non-targeted nursing categories, and the potential impact on a range of nurses' labour market outcomes.	Canadian nursing regulation is the focus
25	Bourgeault IL, Neiterman E, LeBrun J, Viers K, Winkup J. Brain, gain, drain & waste: The experiences of internationally educated health professionals in Canada [Internet]. University of Ottawa; 2010 [cited 2024 Jun 25]. Available from: /https://edmontonsocialplanning.ca/wp-content/uploads/2016/07/edmontonsocialplanning.ca_joomlatools-files_docman-files_F.-SOCIAL-ISSUES_F06-IMMIGRATION_2010-brain_drain.pdf	2010	Grey literature	Research study report	Reports the findings of qualitative comparative research exploring the experiences of internationally educated physicians, nurses, and midwives and the barriers and facilitators related to professional integration in Canada.	Registration/ Licensure	Provincial/ territorial	Multiple (ON, QC, BC, MB)	Key recommendations related to the integration of internationally educated nurses specific to regulation include: language training, financial costs, addressing bureacratic processes, cultural competence, licensure examination, and recognition of prior learning.	Canadian nursing regulation is included in the broader context
26	Brennan M. Managing risk and ensuring quality: Nova Scotia's framework for regulatory excellence. J Nurse Regul. 2013;4(2):39–42.	2013	Discussion paper	N/A	Describes the development of an integrated regulatory excellence framework for the College of Registered Nurses of Nova Scotia (CRNNS) that addresses the concepts of risk management, quality assurance, and quality improvement as well as the relationships among them.	Regulatory Models, Governance Structures, and Reforms	Provincial/ territorial	NS	The regulatory excellence framework encompasses all the CRNNS' innovative regulatory and quality initiatives and provides a framework for risk management. Customizing the definitions of quality assurance and quality improvement and linking them to risk management allowed the CRNNS to clarify and further strengthen its self-regulation mandate in the public interest.	Canadian nursing regulation is the focus
27	British Columbia College of Nurses and Midwives. Findings and recommendations report: Looking back to look forward [Internet]. Novatone; 2022 Dec [cited 2024 May 1]. Available from: https://www.bccnm.ca/Pages/Default.aspx	2022	Grey literature	Regulatory review/audit	Reviews the current state of the British Columbia College of Nurses and Midwives' (BCCNM) complaint process with a cultural safety and humility lens to make process structures, policies, practices, norms, and values safer for Indigenous Peoples.	Conduct/Complaints /Discipline	Provincial/ territorial	BC	Provides specific process guidelines to improve the cultural safety of the regulatory complaints process.	Canadian nursing regulation is the focus
28	Brown S, Elias D. Creating a comprehensive, robust continuing competence program in Manitoba. J Nurse Regul. 2016;7(2):43–52.	2016	Discussion paper	N/A	Reviews the College of Registered Nurses of Manitoba's (CRNM) process in developing its new, robust continuing competence program (CCP).	Continuing competence program	Provincial/ territorial	MB	The revised CCP incorporates a self-development plan, educational tools, practice hours requirements, a review process to assess compliance, and plans for evaluation of the effectiveness of the program. Using the tools together strengthens the program's ability to assure the public that RNs engage in professional lifelong learning and that the regulatory body promotes high standards of knowledge and skill in its CCP.	Canadian nursing regulation is the focus
29	Bryant-Lukosius D, Carter N, Kilpatrick K, Martin-Misener R, Donald F, Kaasalainen S, et al. The clinical nurse specialist role in Canada. Nurs Leadersh. 2010;23(Special Issue):140–66.	2010	Empirical study	Multi-methods (Decision-support synthesis (scoping review and interviews)	Draws on the results of a decision-support synthesis (scoping review and interviews with key informants) to examine advanced practice nursing roles in Canada.	Nursing Roles/Standards	National	N/A	Key challenges to the full integration of CNSs in the Canadian healthcare system include a lack of Canadian research to inform role implementation, absence of a common vision for the role in Canada, lack of a credentialing mechanism, and limited access to CNS-specific graduate education. Recommendations for maximizing the potential and long-term sustainability of the CNS role to achieve important patient, provider and health system outcomes in Canada are provided.	Canadian nursing regulation is included in the broader context
30	Bungay V, Stevenson J. Nurse leaders' experiences of implementing regulatory changes in sexual health nursing practice in British Columbia, Canada. Policy Polit Nurs Pract. 2013;14(2):69–78.	2013	Empirical study	Qualitative (interpretive descriptive - interviews)	Examines the experience of nurse leaders implementing provincial regulatory policy changes affecting expanded nursing practice that facilitates the delivery of sexual health care in British Columbia, Canada.	Nursing Roles/Standards	Provincial/ territorial	BC	Illustrates how competing and contrasting demands between health care and regulatory organizations created substantial barriers to policy change using the examples of preparing for expanded nursing practice certification and the certification process. Results demonstrate that nurse leader engagement is paramount to effective policy implementation.	Canadian nursing regulation is the focus
31	Burgess J, Purkis ME. The power and politics of collaboration in nurse practitioner role development. Nurs Inq. 2010;17(4):297–308.	2010	Empirical study	Qualitative (Participatory action research)	Examines how collaboration advances nurse practitioner (NP) role integration within primary health care from the perspectives of NPs from two health authorities in British Columbia, Canada.	Nursing Roles/Standards	Provincial/ territorial	BC	Discusses structural factors (e.g. legislation and regulation) and the political nature of the NP role. Identifies that interprofessional collaboration positively influences NP role integration, autonomy for role enactment, role clarity, holistic client-centred care, team capacity, and strategic alliances.	Canadian nursing regulation is included in the broader context

32	Byres D, Prodan-Bhalla N, Stevenson RL. Re: CJNL 32.4 - Special focus on the NCLEX-RN Experience in Canada. Nurs Leadersh. 2020;33(3):7–8.	2020	Letter to the editor	N/A	Responds to several published articles that critiqued Canadian nurse regulators in the process of adopting and implementing the NCLEX-RN entry-to-practice exam in 2015.	Registration/ Licensure	National	N/A	Reinforces the quality of processes that were undertaken in the selection of the exam and the national pass rates that improve each year. Suggests that researchers stop focusing on events of the past and instead on developing and implementing a pan-Canadian regulatory framework for nurses in the future.	Canadian nursing regulation is the focus
33	Cairns L, Johansen C. Competency-based assessment of internationally educated nurses. J Nurse Regul. 2013;4(1):45–8.	2013	Discussion paper	N/A	Describes the College of Registered Nurses of British Columbia's (CRNBC) competency-based assessment (CBA) process used to assess the level of educational equivalency of applicants educated outside of Canada. Discusses the alignment of the CBA process with right-touch regulation principles as well as challenges, considerations, and benefits.	Registration/ Licensure	Provincial/ territorial	BC	The CBA process ensures the right applicants are entering practice at the right time after the right educational upgrading.	Canadian nursing regulation is the focus
34	Campbell B, Mackay G. Continuing competence: An Ontario nursing regulatory program that supports nurses and employers. NURS ADM Q. 2001;25(2):22–30.	2001	Discussion paper	N/A	Describes the College of Nurses of Ontario's development of a continuing competence program (CCP) including a self-assessment tool and professional profile.	Continuing competence program	Provincial/ territorial	ON	A CCP that supports competent practitioners through strong practice environment attributes requires stakeholder collaboration and facilitates quality outcomes for the public.	Canadian nursing regulation is the focus
35	Canadian Council of Registered Nurse Regulators. Practice analysis study of nurse practitioners [Internet]. Professional Examination Service; 2015 Dec [cited 2024 May 7]. Available from: /https://www.ccrnr.ca/assets/ccrnr-practice-analysis-study-of-nurse-practitioners-report---final.pdf	2015	Grey literature	Research study report	Outlines the results of a practice analysis, describing the competencies and work of nurse practitioners (NP) across Canada, and explores the evolving nature of their practice. Aims to support a pan-Canadian regulatory framework for NPs with respect to entry-to-practice examination and licensure requirements.	Nursing Roles/Standards	National	N/A	Provides recommendations for national examination development initiatives.	Canadian nursing regulation is the focus
36	Canadian Nurses Association. Canadian nurse practitioner initiative: A 10 year retrospective [Internet]. 2016 [cited 2024 May 8]. Available from: https://www.documentcloud.org/documents/5762376-Canadian-Nurse-Practitioner-Initiative-a-10-Year	2016	Grey literature	Research study report	Evaluates to what extent and in what manner previously recommended actions to improve the integration of nurse practitioners (NP) into the Canadian healthcare system have been enacted. The recommended and evaluated actions are categorized into strategic areas including legislation and regulation, practice, health human resource planning, education, strategic communications, change management and social marketing, evaluation, and governance.	Nursing Roles/Standards	National	N/A	Findings related to regulation include: all jurisdictions have legislation governing NP practice in place as well as title protection. There remain additional designations in various jurisdictions that may create role confusion. Describes the variation in scope of practice, entry-to-practice and licensure validation requirements across jurisdictions.	Canadian nursing regulation is the focus
37	Carson A, Stirling-Cameron E, Paynter M, Munro S, Norman WV, Kilpatrick K, et al. Barriers and enablers to nurse practitioner implementation of medication abortion in Canada: A qualitative study. Cook EJ, editor. PLoS ONE. 2023;18(1):e0280757.	2023	Empirical study	Qualitative (Interpretive description)	Explores the barriers and enablers to nurse practitioner (NP) provision of medication abortion (MA) based on the experiences of nurse practitioners (NPs) and key stakeholders working in government, health administration, nursing regulation, and law.	Nursing Roles/Standards	National	N/A	NPs are well-suited to provide MA but would benefit from MA-specific training integrated into education curricula, widespread communication to raise awareness that NPs can provide this service, and the provision of mentorship from an experience MA provider.	Canadian nursing regulation is included in the broader context
38	Cassiani SHDB, Lecorps K, Rojas Cañaveral LK, da Silva FAM, Fitzgerald J. Regulation of nursing practice in the Region of the Americas. Rev Panam Salud Publica. 2020;44:e93.	2020	Empirical study	Qualitative (Descriptive exploratory comparative analysis)	Compares the current nursing regulations across countries in the Region of the Americas including Canada with a focus on the type of regulatory bodies, requirements for initial professional registration, and registration renewal.	Regulatory Models, Governance Structures, and Reforms	Global	N/A	All countries regulate the nursing profession through a regulatory body. Competency exams for initial registration are required in the United States, Canada, and most Caribbean countries. Registration renewal is required in 54.3% of the countries. Continuing education is required for professional registration renewal in the United States, Canada, and 53% of Caribbean countries. Labor hours are required in the United States and Canada. Regulations promote and protect professional integrity and countries should make efforts to evaluate competency and training, consider the use of competency exams for initial registration, and add continuing education as requirements for registration renewal.	Canadian nursing regulation is included in the broader context
39	Clarke HF, Wearing J. Regulation of registered nursing: The Canadian perspective. Reflect Nurs Leadersh. 2001;27(4):26–35.	2001	Discussion paper	N/A	Describes the regulatory framework for nurses and other health professionals in British Columbia (BC) and discusses the rationale for proposed legislative changes. Provides a list of emerging issues impacting the nursing regulators in Canada including public participation in regulation, evolving scopes of practice, continuing competence, technology, and globalization.	Regulatory Models, Governance Structures, and Reforms	Provincial/ territorial	BC	The challenges in regulating the nursing profession are many and ever-changing and regulators must find the balance in providing flexibility, accountability, and responsibility to the profession and the government while creating appropriate safeguards to protect the public.	Canadian nursing regulation is the focus
40	Coburn D. Professional autonomy and the problematic nature of self-regulation: medicine, nursing and the state. Health & Canadian Society. 1999;5(1):25–53.	1999	Discussion paper	N/A	Examines the changing nature of professional autonomy through self-regulation in medical and nursing professions in Ontario.	Regulatory Models, Governance Structures, and Reforms	Provincial/ territorial	ON	Explores the balance of power between the various stakeholders including the state, regulators, professional associations, and the employers and their influence on self-regulation in Ontario.	Canadian nursing regulation is included in the broader context
41	College of Licensed Practical Nurses of Alberta. An ongoing history of Alberta's licensed practical nurses [Internet]. 2020 [cited 2024 May 1]. Available from: https://www.clpna.com/	2020	Grey literature	Historical review	A historical account of the evolution of the role and regulation of licensed practical nurses in Alberta beginning in 1947.	Regulatory Models, Governance Structures, and Reforms	Provincial/ territorial	AB	Situates the initiation and evolution of LPN regulation in Alberta within the social and political context.	Canadian nursing regulation is the focus
42	College of Licensed Practical Nurses of Alberta. Beginning of self-regulation. Care. 2020;24–30.	2020	Grey literature	Historical review	Outlines key historical events that shaped the evolution of the licensed practical nursing profession in Alberta.	Regulatory Models, Governance Structures, and Reforms	Provincial/ territorial	AB	Outlines key legislative and regulatory milestones in the development of LPN regulation in Alberta.	Canadian nursing regulation is the focus

43	College of Nurses of Ontario, Governance Task Force. Final Report: A vision for the future - Leading in Regulatory Governance [Internet]. 2017 [cited 2024 May 1]. Available from: http://www.cno.org/globalassets/1-whatiscno/governance/final-report---leading-in-regulatory-governance-task-force.pdf	2017	Grey literature	Regulatory review/audit	Provides a best-practice and evidence-based review of all aspects of the College of Nurses of Ontario's (CNO) regulatory governance. Includes recommendations based on current literature, trends, and best-practices in governance internationally.	Regulatory Models, Governance Structures, and Reforms	Provincial/ territorial	ON	Provides recommendations related to council size, diversity, appointment process, and committee roles.	Canadian nursing regulation is the focus
44	College of Registered Nurses of British Columbia. 100 years of nursing regulation 1912-2012 [Internet]. 2012 [cited 2024 May 8]. Available from: https://www.bccnm.ca/Documents/z_centennial/download/CRNBC-Centennial.pdf	2012	Grey literature	Historical review	A historical account of the evolution of nursing regulation in British Columbia beginning in 1912.	Regulatory Models, Governance Structures, and Reforms	Provincial/ territorial	BC	Situates the evolution of nursing regulation in British Columbia within the greater social and political context.	Canadian nursing regulation is the focus
45	Collins EM. Career mobility among immigrant registered nurses in Canada: Experiences of Caribbean women [Internet] [Thesis]. 2004 [cited 2024 Apr 12]. Available from: https://tspace.library.utoronto.ca/handle/1807/118635	2004	Thesis/ Dissertation	Qualitative (interviews)	Explores factors that create barriers or facilitate career mobility among immigrant women of colour from the Caribbean who are registered nurses (RN) in Canada and how their lived experiences as RNs are mediated through race, gender and class.	Registration/ Licensure	National	N/A	Participants developed individual strategies of resilience in response to experiencing significant barriers in navigating their careers as RNs related to systemic practices that influenced the regulation of nursing as well as relationships in the work environment. Proposes antiracism strategies to create equitable status and rewards for immigrant and minority groups in nursing, as well as for the profession as a whole.	Canadian nursing regulation is included in the broader context
46	Covell CL, Neiterman E, Bourgeault IL. Forms of capital as facilitators of internationally educated nurses' integration into the registered nursing workforce in Canada. Canadian Public Policy. 2015;41(Supplement 1):S150–61.	2015	Empirical study	Qualitative (qualitative descriptive)	Explores internationally educated nurses (IEN) experiences of professional integration to provide an understanding of the factors that facilitate their registration and employment as registered nurses (RN) in Canada.	Registration/ Licensure	Provincial/ territorial	Multiple (BC, AB, MB, ON, QC)	The provision of financial resources and interventions to develop their language skills may assist IENs integrate more rapidly into the country's nursing workforce.	Canadian nursing regulation is included in the broader context
47	Creamer AM, Austin W. Canadian nurse practitioner core competencies identified: An opportunity to build mental health and illness skills and knowledge. The Journal for Nurse Practitioners. 2017;13(5):e231–6.	2017	Discussion paper	N/A	Describes the Canadian Council of Registered Nurse Regulators (CCRNR) development of entry-to-practice core competencies for adult, family/all ages and pediatric nurse practitioners (NP) across Canada including the implications for licensure requirements, a national approach to nurse practitioner education, and the need for greater focus on mental health education and skill development.	Nursing Roles/Standards	National	N/A	NPs are well-suited to address mental illness but face barriers related to inadequate and inconsistent mental health education, a lack of relevant clinical practicum experience, as well as financial and time constraints.	Canadian nursing regulation is included in the broader context
48	Curnew D, Deeb A, Isaacs S, Puddester R, Vaughan C. Hindsight Is 2020: A graduate student perspective. Nurs Leadersh. 2022;35(3):32–47.	2022	Discussion paper	N/A	Presents a critique of the Canadian Nurses Association's (CNA) Toward 2020: Visions for Nursing report from a graduate student perspective and examines the extent to which the predictions presented in the report held true in the following areas: health systems as well as nursing practice, workforce, education, and regulation.	Regulatory Models, Governance Structures, and Reforms	National	N/A	Suggests most of the preferred outcomes presented in the original report were unmet or partially met and must further engage with stakeholders, utilize available resources, and conduct research to enact the vision set out in the report. Unmet goals pertaining to nursing regulation related to pan-Canadian license, no additional licensing exam after graduation, a national licensing body regulating all nurses in Canada, IENs being assessed in a national assessment centre using prior learning assessment and recognition to grant licensure as appropriate.	Canadian nursing regulation is included in the broader context
49	Cutcliffe JR, Bajkay R, Forster S, Small R, Travale R. Nurse migration in an increasingly interconnected world: The case for internationalization of regulation of nurses and nursing regulatory bodies. Archives of Psychiatric Nursing. 2011;25(5):320–8.	2011	Discussion paper	N/A	Explores the issue of global variability in nursing regulation in the context of increasing public scrutiny and growing mobilization and globalization of psychiatric/mental health nursing.	Regulatory Models, Governance Structures, and Reforms	Global	N/A	Suggests workforce mobilization and protection of the public would be best served by more standardized disciplinary decision-making across regulatory bodies as well as an international oversight body.	Canadian nursing regulation is the focus
50	Cutcliffe J, Forster S. Professional regulatory nursing bodies: International variation in the protection of the public. International Journal of Nursing Studies. 2010;47(11):1343–5.	2010	Editorial	N/A	Compares and critiques the methods with which nurse regulatory bodies in the United Kingdom and British Columbia, Canada utilize to protect the public.	Regulatory Models, Governance Structures, and Reforms	Global	N/A	Methods used by nurse regulatory bodies to protect the public have limited legitimacy and credibility and suggest that both clinical hours as well as some form of formal continuing education are required to ensure safe practice. Suggests that public safety would be best served by an oversight body.	Canadian nursing regulation is the focus
51	de Witt L, Ploeg J. Critical analysis of the evolution of a Canadian nurse practitioner Role. Can J Nurs Res. 2005;37(4):116–37.	2005	Discussion paper	N/A	Analyzes the evolution of a nurse practitioner (NP) role in Canada using the province of Ontario as an example.	Nursing Roles/Standards	Provincial/ territorial	ON	NPs can engage in advocacy, lobbying, and public education to counter barriers inhibiting the full integration of NPs within primary health care in Ontario including the lack of an effective funding plan, restrictions on scope of practice, work-related tensions between physicians and NPs, and lack of public and professional awareness of the role.	Canadian nursing regulation is included in the broader context
52	Delamaire M, Lafortune G. Nurses in advanced roles: A description and evaluation of experiences in 12 developed countries [Internet]. 2010 [cited 2024 Jun 3]. (OECD Health Working Papers; vol. 54). Report No.: 54. Available from: https://www.oecd-ilibrary.org/social-issues-migration-health/nurses-in-advanced-roles_5kmbrcfms5g7-en	2010	Grey literature	Research study report	Reviews the development of advanced practice nurses in 12 countries (Australia, Belgium, Canada, Cyprus, Czech Republic, Finland, France, Ireland, Japan, Poland, the United Kingdom and the United States), with a particular focus on their roles in primary care. Outlines reasons motivating the development of the role, its current state of development, an evaluation of care quality and cost, as well as barriers and facilitators to practice which includes legislation and regulation, among other topics.	Nursing Roles/Standards	Global	N/A	Describe the role and scope of nurse practitioners (NP) and clinical nurse specialists in Canada. Government legislation and regulation are identified as a key facilitator to the integration of the NP role. Cites a Canadian study describing recommendations for future directions including the standardization of regulatory and education standards and requirements.	Canadian nursing regulation is included in the broader context
53	Desrosiers G. Is Quebec moving towards professional deregulation? Infirm Que. 1997;4(4):4–7.	1997	Editorial	N/A	Critiques the provincial professional regulatory system and suggests reform will be difficult due to resistance from regulators.	Regulatory Models, Governance Structures, and Reforms	Provincial/ territorial	QC	Suggests reform is unlikely as regulators are focused on maintaining protection of their title and distinct scope of practice rather than reducing barriers to professional practice.	Canadian nursing regulation is included in the broader context

54	DiCenso A, Martin-Misener R, Bryant-Lukosius D, Bourgeault I, Kilpatrick K, Donald F, et al. Advanced practice nursing in Canada: Overview of a decision support synthesis. Nurs Leadersh. 2010;23(SP):15–34.	2010	Empirical study	Multi-methods (decision support synthesis including literature review and interviews)	Describes the distinguishing characteristics of clinical nurse specialist (CNS) and nurse practitioner (NP) roles, and expands upon the barriers, facilitators, and recommendations for the development and utilization of these roles in Canada.	Nursing Roles/Standards	National	N/A	Findings demonstrate unrealized contributions of advanced practice nurses (APN) could have a significant impact on access to high-quality health care. Competing interests of stakeholders including government and regulators create barriers to effective deployment of APNs and APNs remain vulnerable to changes in health policy and economic conditions. Regulation-specific recommendations include standardizing APN standards, requirements, and processes nationally.	Canadian nursing regulation is included in the broader context
55	Duncan S, Whyte N. British Columbia's one nursing regulator: A critical commentary on the amalgamation process. Nurs Leadersh. 2018;31(3):24–33.	2018	commentary	N/A	Provides a critique of British Columbia's transition to one nursing regulator in 2018, discusses amalgamation processes including professional regulation and governance, the preservation of nursing history, nurses' fees and resources, and role distinctions and identities in registrant groups.	Regulatory Models, Governance Structures, and Reforms	Provincial/ territorial	BC	Questions the efficacy of the amalgamated nurse regulatory body and its governance structure. Suggests that the nursing profession's collective engagement and nursing leadership in self-regulation and decision-making related to professional practice is key to public safety and integral to the identity of Canadian nursing.	Canadian nursing regulation is the focus
56	Duncan S, Thorne S, Rodney P. Evolving trends in nurse regulation: what are the policy impacts for nursing's social mandate? Nursing Inquiry. 2015;22(1):27–38.	2015	Empirical study	Qualitative (Critical policy analysis)	Presents a critical policy analysis of the impact of recent regulatory trends on what the International Council of Nurses considers nursing's three 'pillars' – the profession of nursing, socioeconomic welfare of nurses, and nurse regulation.	Regulatory Models, Governance Structures, and Reforms	National	N/A	Themes arising from the analysis include regulatory discontinuity, tightening of regulatory control, and an increasingly managerial governance culture. The strength and synergy of the three professional pillars including the association, union, and regulator, are essential for the enactment of organizational and professional values.	Canadian nursing regulation is the focus
57	Durcan R, Richler E, Steinecke R. Major regulatory reform comes to Canada. J Nurse Regul. 2023;14(2):43–8.	2023	Discussion paper	N/A	Discusses the regulatory implications of British Columbia's new Health Professions and Occupations Act, particularly those consistent with regulatory reform initiatives elsewhere in Canada and the United Kingdom.	Regulatory Models, Governance Structures, and Reforms	Provincial/ territorial	BC	Describes the new Act as comprehensive and innovative with immediate implications including the end of self-regulation, smaller and competency-based boards, separation of the board from regulatory committees, amalgamation of regulators, and establishment of an oversight Superintendent, along with other initiatives.	Canadian nursing regulation is included in the broader context
58	Forchuk C, Kohr R. Prescriptive authority for nurses: The Canadian perspective. Perspectives in Psychiatric Care. 2009;45(1):3–8.	2009	Discussion paper	N/A	Describes the role, barriers, and facilitators of prescriptive authority for nurses within Canada.	Nursing Roles/Standards	National	N/A	Prescriptive authority is linked to the development of advanced practice nursing in Canada and role clarity, strength in nursing focus, and support from administrators and legislation are required for prescriptive authority to be fully enacted.	Canadian nursing regulation is included in the broader context
59	Foster S. Practice support initiatives that contribute to the regulation of registered nurses and nurse practitioners. J Nurse Regul. 2012;2(4):19–25.	2012	Discussion paper	NA	Describes mechanisms through which practice support is provided by the College of Registered Nurses of British Columbia (CRNBC) to help registrants apply standards and meet expectations for professional practice.	Nursing Roles/Standards	Provincial/ territorial	BC	Describe the CRNBC's framework guiding the practice support program as well as the related initiatives, practice consultation services, learning events, professional support program, and application tools.	Canadian nursing regulation is the focus
60	Fuller E, Kneeshaw C, Baumann A, Deber R. Identifying standards, meeting expectations: Comparing regulatory standards and community health nursing practice standards. CAN NURSE. 2008;104(7):30–4.	2008	Empirical study	Qualitative (content analysis)	Presents a comparison of the national standards of practice developed for community health nurses by the Community Health Nurses Association of Canada (CHNC) with the standards of practice developed by provincial/territorial regulatory bodies to assess the similarity and dissimilarity of frameworks in the context of community care to further understanding of the expectations of practice with the shift from hospital to community settings.	Nursing Roles/Standards	National	N/A	There was noted diversity across the studied documents which suggests a need for associations and specialty groups, notably national ones, to integrate regulatory standards into their own documents and build upon them to reflect the dimensions of specialty practice.	Canadian nursing regulation is the focus
61	Garrett BM, MacPhee M. The slippery slope of nursing regulation: Challenging issues for contemporary nursing practice in Canada. Nurs Leadersh [Internet]. 2014 [cited 2024 Apr 15];27(3).	2014	Discussion paper	NA	Explores and critiques the current organizational structure of nursing regulation and its impact on nursing advocacy in Canada using contemporary issues associated with nursing regulation and scope of practice as examples.	Regulatory Models, Governance Structures, and Reforms	National	N/A	Present examples to demonstrate fragmentation in nursing regulation and disciplinary processes and provide recommendations for future policy and practice to enhance regulatory clarity and consistency.	Canadian nursing regulation is the focus
62	Governance Solutions. CARNA Governance Executive Summary and Recommendations [Internet]. 2020 Aug [cited 2024 May 7]. Available from: https://www.nanb.nb.ca/trends-in-nursing-regulation/	2020	Grey literature	Regulatory review/audit	Provides a summary of recommendations to enhance the governance structure within the College of Registered Nurses of Alberta (CARNA).	Regulatory Models, Governance Structures, and Reforms	Provincial/ territorial	AB	Provides recommendations related to representing a single mandate as a regulatory organization, transitioning to a new governance model, reforming council committees, aligning with regulatory philosophy, and reforming council size, composition, and appointment.	Canadian nursing regulation is the focus
63	Guerrette-Daigle L, Landry V, Harrison S, Durocher-Hendriks S, Marquis FL, Auffrey LM, et al. The NCLEX-RN as an entry-to-practice exam in New Brunswick: The rocky road story of Francophone candidates to the nursing profession. Nurs Leadersh. 2019;32(4):74–80.	2019	Discussion paper	NA	Describes the experience of adopting the NCLEX-RN as an entry-to-practice exam among francophone baccalaureate nursing students in New Brunswick, Canada.	Registration/ Licensure	Provincial/ territorial	NB	Describes the advocacy efforts related to combatting inequities faced by Francophone nursing students created by the introduction of the NCLEX-RN as an entry-to-practice exam due to inadequate translation of the exam and its related resources.	Canadian nursing regulation is included in the broader context
64	Hadley M. Nursing practice in Canada: The influence of current and proposed legislation. Journal of Advanced Nursing. 1995;22(6):1210–7.	1995	Discussion paper	NA	Describes the legislation governing nursing regulation in Canada and discusses the implications of the Agreement on Internal Trade (1994) and the North American Free Trade Agreement (NAFTA) on regulation and the related mobility of nurses across provincial jurisdictions.	Regulatory Models, Governance Structures, and Reforms	National	N/A	Highlight barriers to pan-Canadian regulatory standards for nurses related to the federated Canadian political framework and caution political leaders in treating health care professional services as a commodity but instead focus on establishing a balance between economic and socio-political goals with advocating for public safety and competent and ethical care.	Canadian nursing regulation is the focus

65	Hall LM, Peterson J, Price S, Andrews G, Lalonde M, MacDonald-Rencz AH and S. I was never recruited: Challenges in cross-Canada nurse mobility. Nurs Leadersh. 2013;26(SP):29–40.	2013	Empirical study	Mixed methods (Descriptive cross-sectional survey)	Maps mobility patterns of Canadian nurses across provincial and territorial jurisdictions and identifies factors influencing nurses to leave their home province or territory to work in another. Discusses facilitators that can be employed to retain nurses in Canada.	Registration/ Licensure	National	N/A	Identified barriers to the inter-jurisdictional mobility of nurses include a lack of recruitment incentives, lengthy and inconsistent licensing processes, and lack of information about employment positions available across Canada which present opportunities for collaborative action to improve mobility and retention of nurses in Canada.	Canadian nursing regulation is included in the broader context
66	Hallaran A, McNabb A, Anderson J. Developing a principle-based approach to safe medication practices. J Nurse Regul. 2015;6(3):43–7.	2015	Discussion paper	N/A	Describes the development of the new principle-based medication practice standard by the College of Nurses of Ontario in the context of a rapidly changing healthcare environment including the process, collaborations, and methods used.	Nursing Roles/Standards	Provincial/ territorial	ON	The approach builds on the expectations of nurses to use critical thinking and problem-solving skills to achieve safe, ethical nursing practice. With this approach, the document sets standards that can be seen as benchmarks for practice in ever-changing practice environments and supports the regulator in achieving the mandate of public protection.	Canadian nursing regulation is the focus
67	Hamilton-Jones M. The complaints process in Ontario: Analyzing the experiences of nurses and complainants. J Nurse Regul. 2016;7(2):21–6.	2016	Empirical study	Mixed methods (Survey)	Describes the results of surveys assessing both complainants' and registrants' satisfaction with the College of Nurses of Ontario's (CNO) complaints process and their perceptions of the effectiveness of the process in remediating nurses and protecting the public.	Conduct/Complaints /Discipline	Provincial/ territorial	ON	Responses show that a majority believe that participation in the complaints process contributes to their professional development, improves their understanding of how practice standards relate to their practice, and leads to practice improvements. Members of the public whose complaints were addressed through the CNO's alternative dispute resolution process are more satisfied than those whose complaints were addressed through the investigation process and were also more likely to agree that the process protects the public.	Canadian nursing regulation is the focus
68	Harvey JL. Exploring what quality means to licensed practical nurses and implications for professional practice [Internet] [M.A.]. [British Columbia, CA]: Royal Roads University; 2015 [cited 2024 Apr 15]. Available from: https://www.proquest.com/docview/1738992111/abstract/5BCAB8EAA8984527PQ/1	2015	Thesis/ Dissertation	Mixed methods (Action research - interviews and survey)	Describes the perceptions of licensed practical nurses (LPNs) registered with the College of Licensed Practical Nurses of British Columbia (CLPNBC) regarding the meaning and implications of quality on professional nursing practice.	Nursing roles/standards	Provincial/ territorial	BC	Findings indicate LPNs placed considerable importance on the acquisition of competence, were sensitive to how their colleagues perceived their value as part of the nursing team and expressed confusion as to how CLPNBC should advocate for quality practice among registrants. Provides recommendations to CLPNBC to support quality professional practice among its registrants.	Canadian nursing regulation is the focus
69	Hawkins M, Rodney P. A precarious journey: Nurses from the Philippines seeking RN licensure and employment in Canada. CJNR. 2015;47(4):97–112.	2015	Empirical study	Qualitative (ethnography with Postcolonial feminism theoretical lens)	Examines the experiences of nurses educated in the Philippines as they seek Canadian RN licensure and employment, critically examining structures and processes that create barriers at the international, national, and local levels.	Registration/ Licensure	National	N/A	Found that nurse migration is a multifaceted phenomenon fraught with ethical and practical concerns about the nurses' well-being as well as the safety and competency of their practice, highlighting tensions within and between the contexts of shifting regulatory and immigration policies. A key finding relates to complex licensing requirements as a significant barrier to practicing nursing in Canada and recommendations for regulators include striving toward universal standards for licensing through collaboration with stakeholders.	Canadian nursing regulation is included in the broader context
70	Heale R, Rieck Buckley C. An international perspective of advanced practice nursing regulation. International Nursing Review. 2015;62(3):421–9.	2015	Empirical study	Mixed methods (Surveys)	Examines the status of advanced practice nursing regulation globally.	Nursing Roles/Standards	Global	N/A	Despite the support available through the International Council of Nurses and International Nurse Practitioner/Advanced Practice Nursing Network, there continues to be a wide variation of advanced practice nursing regulation and credentialing criteria.	Canadian nursing regulation is included in the broader context
71	Hicks B. Gender, politics, and regionalism: Factors in the evolution of registered psychiatric nursing in Manitoba, 1920-1960. Nursing History Review. 2011;19:103–26.	2011	Empirical study	Qualitative (Historical method)	Explores the historical evolution and professionalization of the Registered Psychiatric Nurses (RPN) profession in Manitoba from 1920-1960.	Regulatory Models, Governance Structures, and Reforms	Provincial/ territorial	MB	There were significant contextual, contingent, political, and gendered influences that impacted the evolution and professionalization of RPNs in Manitoba.	Canadian nursing regulation is the focus
72	Howlett MK, Tamlyn D. Advanced practice nursing: Parameters for successful integration. Healthc Manage Foru. 1999;12(3):12–8.	1999	Empirical study	Mixed methods (Survey)	Examines advanced practice nursing (APN) in Canada by reviewing five areas of strategic importance: (1) definitions and scope of practice; (2) education, credentialing and regulation; (3) new roles in healthcare; (4) costs and benefits in health reform; and (5) implementation and relationship issues.	Nursing Roles/Standards	National	N/A	The Canadian health services environment is best served by a multi-faceted APN role, defined by a nursing paradigm that invokes collaborative relationships with physicians, and education and credentialing that is based on national standardization.	Canadian nursing regulation is included in the broader context
73	Hurlock-Chorostecki C, Goodwin M van S and S. The acute care nurse practitioner in Ontario: A workforce study. Nurs Leadersh. 2008;21(4):100–16.	2008	Empirical study	Quantitative (Cross-sectional survey)	Presents information through self-reported surveys on nurse practitioners (NP) working in acute care settings who are not currently regulated in the extended class in Ontario.	Nursing Roles/Standards	Provincial/ territorial	ON	Continued barriers exist related to legislation and regulation of the NP role in Ontario, such as a lack of registration within the extended class. Results suggest wide acceptance of the role is concentrated around academic teaching hospitals, however, there is inadequate understanding and support for the multiple aspects of this role beyond clinical practice.	Canadian nursing regulation is included in the broader context
74	Ismail F, Clarke SP. Canadian nursing supervisors' perceptions of monitoring discipline orders: Opportunities for regulator-employer collaboration. J Nurse Regul. 2016;6(4):68–72.	2016	Empirical study	Quantitative (Cross-sectional survey)	Describes a needs assessment undertaken by the College of Nurses of Ontario (CNO) to understand employers' perspectives on collaborating with regulators to monitor nurses practicing with conditions and restrictions resulting from a discipline order by a regulator.	Conduct/Complaints /Discipline	Provincial/ territorial	ON	Programs should focus less on the general philosophy of remediation, discipline orders, and the idea of employer participation and focus instead on directly addressing practical issues and concerns, especially those related to attitudes toward hiring nurses with discipline orders and the feasibility of integrating nurses with practice restrictions into their staff.	Canadian nursing regulation is the focus
75	Jeans ME. In-country challenges to addressing the effects of emerging global nurse migration on health care delivery. Policy Polit Nurs Pract. 2006;7(3):58–61.	2006	Empirical study	Mixed methods (survey, interviews, focus groups)	Assesses current practices, policies, and challenges related to the licensure of international nurse applicants and their integration into the workforce in Canada.	Registration/ Licensure	National	NA	An integrated approach is necessary to address the gaps in regulation, screening, assessment, and registration of internationally educated nurses and related ethical, cultural, and safety issues affecting international nurse migrants. A series of recommendations are provided.	Canadian nursing regulation is included in the broader context
76	Johansen C, Cairns L. Provisional RN registration: Opening doors and shifting practices in nurse regulation. J Nurse Regul. 2012;3(3):51–4.	2012	Discussion paper	N/A	Describes the experience of introducing a new classification (provisional registration) of registered nurse (RN) registration in British Columbia, Canada.	Regulatory Models, Governance Structures, and Reforms	Provincial/ territorial	BC	The provisional registration class allowed CRNBC to challenge the status quo of the registration application process for IEN applicants and apply a different lens to how IENs are assessed.	Canadian nursing regulation is the focus

77	Johansen C, Chisholm B, Secong D, Sihat A, Amratlal A, McGraw S. Building competency-based practice into democratically elected boards. J Nurse Regul. 2020;10(4):4–12.	2020	Discussion paper	N/A	Investigates the meaning, evolution, and implementation of competency-based boards within the corporate and not-for-profit realms that informed changes to the British Columbia College of Nursing Professionals's (BCCNP) board.	Regulatory Models, Governance Structures, and Reforms	Provincial/ territorial	BC	Competency-based boards and committees represent current best practices in governance as they help regulatory bodies ensure accountability to the government and the mandate to act in the public interest.	Canadian nursing regulation is the focus
78	Johansen C, Knowles B, Jones D, Dickison P. Commentary on McGillis Hall, L., Lalonde, M., Kashin, J., Yoo, C., & Moran, J. (2017) Changing nurse licensing examinations: Media analysis and implication of the Canadian experience. International Nursing Review. 2018;65(2):159–60.	2018	Commentary	N/A	Critiques the methodology utilized in the article published by McGillis Hall et al. (2017), a media analysis of the change in registered nurse licensing requirements to the NCLEX-RN standardized examination.	Registration/ Licensure	National	N/A	Questions the credibility and reliability of the researchers' conclusions and suggests that such biased views regarding the implementation of the NCLEX-RN standardized entry-to-practice examination create confusion through misinformation.	Canadian nursing regulation is the focus
79	John R, Marchand V, Marques M. The Canadian Nursing Students' Association and its advocacy for a culturally competent entry-to-practice exam. Nurs Leadersh. 2019;32(4):92–6.	2019	Discussion paper	N/A	Discusses the Canadian Nursing Students' Association (CNSA) advocacy efforts in support of nursing students as they write the NCLEX-RN entry-to-practice examination.	Registration/ Licensure	National	N/A	Although some progress has been made, CNSA recognizes that work still needs to be done to address its concerns that the NCLEX-RN needs to better reflect Canadian healthcare system values including consideration of cultural competence for Canada's First Nations, Inuit, and Métis populations; properly reflect the focus of Canadian nursing programs; and represent Canada's two official languages.	Canadian nursing regulation is included in the broader context
80	Kaasalainen S, Martin-Misener R, Kilpatrick K, Harbman P, Bryant-Lukosius D, Donald F, et al. A historical overview of the development of advanced practice nursing roles in Canada. Nurs Leadersh. 2010;23(SP):35–60.	2010	Empirical study	Historical analysis (draws on previous scoping review and interviews for a decision support synthesis)	Provides a historical overview of the major influences on the development of advanced practice nursing (APN) roles, including the nurse practitioner and clinical nurse specialist, that exist in Canada today.	Nursing Roles/Standards	National	N/A	Advanced practice nursing in Canada has evolved in response to a number of influential factors that have both facilitated and hindered the development of these roles which have grown to meet gaps and emerging needs in the healthcare system. Legislation and regulation are discussed as key events and influences in the evolution of these roles.	Canadian nursing regulation is included in the broader context
81	Kilpatrick K, DiCenso A, Bryant-Lukosius D, Ritchie JA, Carter RMM and N. Clinical nurse specialists in Canada: Why are some not working in the role? Nurs Leadersh. 2014;27(1):62–75.	2014	Empirical study	Mixed-methods (Cross-sectional survey)	Summarizes the perspectives of Canadian clinical nurse specialists (CNS) and the factors associated with their non-employment in the CNS role.	Nursing Roles/Standards	National	N/A	Lack of role clarity, inability to find employment as a CNS, and the inability to implement all the dimensions of the CNS role were key factors in CNS' decision not to work in the role. These factors are potentially modifiable and amenable to decisions made by nursing leaders in organizations and regulatory bodies.	Canadian nursing regulation is included in the broader context
82	Kilpatrick K, Harbman P, Carter N, Martin-Misener R, Bryant-Lukosius D, Donald F, et al. The acute care nurse practitioner role in Canada. Nurs Leadersh. 2010;23(Special Issue):114–39.	2010	Empirical study	Multi-method (Decision support synthesis (scoping review and interviews)	Describes the current status of acute care nurse practitioner (ACNP) roles in Canada, including an overview of ACNP education, legislation and regulation, deployment and work setting, and key issues influencing the full integration of ACNP roles into the Canadian healthcare system.	Nursing Roles/Standards	National	N/A	The status of ACNP roles continues to evolve across Canada, influenced by barriers, including incomplete utilization of role components, limitations to the scope of practice, inconsistent team acceptance and funding issues and facilitators, including clear and tailored communication about the role, supportive leadership of healthcare managers, and stable and predictable funding.	Canadian nursing regulation is included in the broader context
83	Kolawole B. Ontario's internationally educated nurses and waste in human capital. International Nursing Review. 2009;56(2):184–90.	2009	Discussion paper	NA	Critically analyzes the waste in the human capital of Ontario's internationally educated nurses (IEN) resulting from unemployment or underemployment.	Registration/ Licensure	Provincial/ territorial	ON	IENs face significant barriers related to immigration and professional licensure requirements and processes that prevent their integration into the Ontario healthcare system, resulting in 'brain waste' which has severe financial, social, and economic consequences both in Ontario and globally.	Canadian nursing regulation is included in the broader context
84	Kunyk D, Deschenes S. Disciplinary decisions regarding professional nurses: Exploring regulatory decisions in a western Canadian province. J Nurse Regul. 2019;10(3):28–33.	2019	Empirical study	Quantitative (Retrospective study)	Describes the prevalence, violations, licensure restrictions, sanctions, and license conditions detailed in professional nursing disciplinary decisions in a Western Canadian province (Alberta) over a 10-year period.	Conduct/Complaints /Discipline	Provincial/ territorial	AB	A small proportion of nurses are disciplined in a given year (less than 0.5%) and more than 60% of all disciplinary investigations fall into the broad categories of negligence/professional incompetence and unprofessional conduct. One key finding of this study is that the vast majority of all investigations were referred to a disciplinary hearing rather than less punitive interventions at the disposal of the regulatory body.	Canadian nursing regulation is the focus
85	Kunyk D, Milner M, Overend A. Disciplining virtue: Investigating the discourses of opioid addiction in nursing. Nursing Inquiry. 2016;23(4):315–26.	2016	Empirical study	Qualitative (critical discourse analysis)	Critically analyzes underlying discourses of addiction that come to shape the disciplinary approaches of a nursing regulatory body that upheld popular assumptions of addiction as an autonomous, rational choice replete with individual-based consequences as read through the popular media coverage.	Conduct/Complaints /Discipline	Provincial/ territorial	Anonymized	Themes that emerged from the analysis include: (i) the framing of addiction as a personal choice; (ii) a focus on the moral character of the nurses specifically as it relates to their duty, professional conduct and trust; (iii) a failure to adequately contextualize addiction as disease arguments; and finally (iv), a lack of discussion of both personal and systemic contextual factors involved in the nurses' cases.	Canadian nursing regulation is the focus
86	Kwan J a., Wang M, Cummings G g., Lemermeyer G, Nordstrom P, Blumer L, et al. The evaluation of evidence-informed changes to an internationally educated nurse registration process. International Nursing Review. 2019;66(3):309–19.	2019	Empirical study	Mixed methods (pre–post-quasi-experimental design and a qualitative evaluation)	Explores the effectiveness of implemented policy and practice changes based on findings from a retrospective review of administrative data and seeks to evaluate whether using evidence-based policy increased the efficiency and transparency of the registration process for IENs.	Registration/ Licensure	Provincial/ territorial	AB	The policy and practice changes resulted in reduced time between steps in the process and stakeholders reported an increase in perceived efficiency, transparency, and use of evidence.	Canadian nursing regulation is the focus
87	Kwan J, Suyat L, Lemermeyer G, Giblin C. Improving Alberta's registration process for internationally educated nurses. J Nurse Regul. 2017;7(4):41–5.	2017	Discussion paper	N/A	Describes the development of policies and processes implemented by the College and Association of Registered Nurses of Alberta (CARNA) to bridge the gaps between IEN competencies and those required by CARNA.	Registration/ Licensure	Provincial/ territorial	AB	In response to identified gaps, policy and process changes included adjustments to the management of bridging education, an Assessment Criteria Checklist, communications regarding the application process, and new timelines for IEN applications.	Canadian nursing regulation is the focus

88	Lalonde M. The forgotten: The challenges faced by Francophone nursing candidates following the introduction of the NCLEX-RN in Canada. Nurs Leadersh. 2019;32(4):66–73.	2019	Discussion paper	N/A	Discusses the decreasing number of francophone graduates writing the NCLEX-RN in French due to a lack of French-language preparatory materials, creating inequities for francophone exam writers.	Registration/ Licensure	National	N/A	Canadian nursing regulators need to actively work with the francophone nursing community to determine their needs and create concrete strategies to meet those needs, including collaborating with the NCSBN to translate the online NCLEX-RN reviews.	Canadian nursing regulation is included in the broader context
89	Lankshear S, Martin D. Getting comfortable with "it depends": Embracing the impermanence of scope of practice. Nurs Leadersh. 2019;32(1):30–41.	2019	Discussion paper	N/A	Presents an overview of scope of practice and challenges nurses experience when navigating the complexities of legislation, regulation, education, and organizational factors that all contribute to the perceived ambiguity and role confusion within the nursing profession.	Nursing Roles/Standards	Provincial/ territorial	ON	The dynamic nature of scope of practice requires all nurses to think differently about their roles and embrace the professional autonomy enabled through legislation, regulation and education rather than trying to address role intersections, ambiguity or confusion by prescribing a definitive answer.	Canadian nursing regulation is the focus
90	Lemermeyer G, Sadesky G. The gamification of jurisprudence: Innovation in registered nurse regulation. J Nurse Regul. 2016;7(3):4–10.	2016	Empirical study	Quantitative (pre-post test design)	Explores an innovative module using the principles of online games—known as gamification— to educate registered nurses in jurisprudence and to assess their competence in this knowledge.	Continuing competence program	Provincial/ territorial	AB	Initial results from pilot testing indicate that the module delivers a valid assessment of jurisprudence knowledge and participant engagement with the content.	Canadian nursing regulation is the focus
91	Leslie K, Cecilia C, Freeman A, Bourgeault I. The regulatory amalgamation for nursing and midwifery in British Columbia. HRO-ORS [Internet]. 2021 [cited 2024 May 16];9(1). Available from: https://mulpress.mcmaster.ca/hro-ors/article/view/4510	2021	Discussion paper	N/A	Analyzes the amalgamation process combining the separate nurse and midwife regulators in British Columbia (BC).	Regulatory Models, Governance Structures, and Reforms	Provincial/ territorial	BC	The amalgamation was influenced by a call both in Canada and Internationally for more efficient and effective regulation. Choosing to amalgamate rather than waiting until mandated allowed the regulators to proactively shape the process.	Canadian nursing regulation is the focus
92	Leslie K, Dunk M, Staempfli S, Cook K. Mandatory reporting of colleagues to regulators: An overview of requirements for registered nurses in 12 Canadian jurisdictions. J Nurse Regul. 2021;12(3):68–77.	2021	Empirical study	Qualitative (documentary content analysis)	Compares the comprehensiveness and content of legislative requirements for registered nurses (RNs) to report concerns about colleagues to regulatory bodies across 12 Canadian jurisdictions.	Conduct/Complaints /Discipline	National	N/A	There is considerable variation in the legislative mandatory reporting frameworks across these Canadian jurisdictions, primarily in the scope of reportable conduct and the threshold that triggers the requirement to report.	Canadian nursing regulation is the focus
93	Leslie K, Myles S, Stahlke S, Schiller CJ, Shelley JJ, Cook K, et al. Regulating during crisis: A qualitative comparative case study of nursing regulatory responses to the COVID-19 pandemic. J Nurse Regul. 2023;14(1):30–41.	2023	Empirical study	Qualitative (comparative case study - interviews and documents)	Analyzes regulatory bodies' responses during the pandemic, examines how nursing regulators conceptualize the public interest during a public health crisis, and explores the influence of a public health crisis on the balancing of regulatory principles.	Regulatory Models, Governance Structures, and Reforms	Global	N/A	Five themes were constructed from the data: (1) risk-based responses to reduce regulatory burden; (2) agility and flexibility in regulatory pandemic responses; (3) working with stakeholders for a systems-based approach; (4) valuing consistency in regulatory approaches across jurisdictions; and (5) the pandemic as a catalyst for innovation. The meaning of "public interest" in the context of high workforce demand was a key consideration for regulators which represents a shift in thinking around broader public interest issues, beyond the conduct and competence of individual nurses, to include pressing societal issues.	Canadian nursing regulation is the focus
94	Leslie K, Nelson S, Deber R, Gilmour J. Policy tensions in regulatory reform: Changes to regulation of health professions in Australia, the United Kingdom, and Ontario, Canada. J Nurse Regul. 2018;8(4):32–42.	2018	Empirical study	Qualitative (comparative multiple case study analysis)	Compares recent reforms to health profession regulatory frameworks in Australia, the United Kingdom, and Ontario, Canada.	Regulatory Models, Governance Structures, and Reforms	Global	N/A	Common themes existed across the reforms: a shift in each jurisdiction towards a more overt primacy of the public interest over professional interests, greater independence of regulation from the professions, a push towards collaboration and consistency between professional regulators, and a focus on articulating principles to aid in assessing regulatory quality.	Canadian nursing regulation is the focus
95	Leurer MD. Lessons in media advocacy: A look back at Saskatchewan's nursing education debate. Policy Polit Nurs Pract. 2013;14(2):86–96.	2013	Empirical study	Qualitative (Media analysis)	Examines the media advocacy techniques utilized by nursing stakeholders in Saskatchewan, Canada in response to a new government policy that would have impacted educational requirements for licensure as a registered nurse (RN) in that province.	Regulatory Models, Governance Structures, and Reforms	Provincial/ territorial	SK	The success of nursing stakeholders in reversing the government's position highlights the effectiveness of media advocacy as a tool to disseminate messages from the nursing profession in order to impact policy.	Canadian nursing regulation is included in the broader context
96	Little L. Nurse migration: A Canadian case study. Health Services Research. 2007;42(3p2):1336–53.	2007	Discussion paper	NA	Synthesizes information about nurse migration in and out of Canada and analyzes its role as a policy lever to address the Canadian nursing shortage.	Registration/ Licensure	National	N/A	The current and projected shortage of nurses in Canada is a product of healthcare cost containment policies that failed to take into account long-term consequences for nurse workforce adequacy. National policies to increase domestic nurse production and retention are recommended in addition to international collaboration among developed countries to move toward greater national nurse workforce self-sufficiency.	Canadian nursing regulation is included in the broader context
97	Logie A, Geiger-Brown J. Do RNs in British Columbia work excessive hours? A registry data study. J Nurse Regul. 2017;7(4):52–60.	2017	Empirical study	Quantitative (cross-sectional analysis of secondary data)	Assesses the prevalence of excessive work hours among College of Registered Nurses of British Columbia (CRNBC) registrants as reported during their licensure renewal as well as the factors associated with excessive hours, including age, sex, Canadian or foreign education, area of specialization, and multiple jobs.	Conduct/Complaints /Discipline	Provincial/ territorial	BC	Found that 6.5% of nurses (2,090 of 32,142) worked more than 2,000 hours during the study year and that 1.4% (466 of 32,142) exceeded 2,500 hours. Males; older nurses; foreign-educated nurses; critical care, operating room, geriatric, administration, education, and research nurses; and nurses with multiple jobs worked excessive hours more frequently than their counterparts. Discusses an unexpected association of excessive work hours with professional conduct review and the potential for collaboration between employers and regulators.	Canadian nursing regulation is included in the broader context
98	Lum L, Dowedoff P, Englander K. Internationally educated nurses' reflections on nursing communication in Canada. International Nursing Review. 2016;63(3):344–51.	2016	Empirical study	Qualitative (grounded theory)	Explores internationally educated nurses' perceptions of the English language and nursing communication skill requirements in a Canadian bridging education program.	Registration/ Licensure	Provincial/ territorial	ON	Participants possessed varying degrees of comprehension and acceptance of Canadian educational and professional regulatory requirements. A certain degree of culture shock, which may be associated with frustration and disillusionment, is a typical and anticipated aspect of the immigration process which may be mitigated by clear communication from regulators about English language and nursing communication requirements during the pre-arrival period.	Canadian nursing regulation is included in the broader context
99	Maciura J. Trying to make sense of the use of social media by practitioners. Grey Areas: A Commentary on Legal Issues Affecting Professional Regulation [Internet]. 2020 Oct [cited 2024 May 7]; Available from:https://www.sml-law.com/wp-content/uploads/2020/10/Greyar250.pdf	2020	Grey literature	Legal commentary	Provides a commentary on the results of a regulatory discipline decision regarding a nurse's social media post and explores the complications of regulating professionals in this space.	Conduct/Complaints /Discipline	National	N/A	Outlines the proceedings and implications of a Canadian nurse who was disciplined by the regulator for their social media use.	Canadian nursing regulation is included in the broader context

100	Maciura J. Radical governance and restrained complaints reforms in British Columbia. Grey Areas: A Commentary on Legal Issues Affecting Professional Regulation [Internet]. 2020 Sep [cited 2024 May 7]; Available from: https://www.sml-law.com/wp-content/uploads/2020/09/Greyar249.pdf	2020	Grey literature	Legal commentary	Provides a commentary on a report that included recommendations to modernize the British Columbia health profession regulatory framework.	Regulatory Models, Governance Structures, and Reforms	Provincial/ territorial	BC	Provides commentary on recommendations to modernize regulation related to cultural safety and humility, governance, as well as complaints and discipline.	Canadian nursing regulation is included in the broader context
101	Maciura J, Wade A. How off-duty use of social media may lead to professional discipline. J Nurse Regul. 2019;9(4):31–3.	2019	Discussion paper	NA	Details a disciplinary decision of the Saskatchewan Registered Nurses Association (SRNA) in relation to "off-duty" social media use and discusses the responsibility of regulators to assess and investigate these complaints in accordance with their governing legislation, code of ethics, rules, standards, and regulations.	Conduct/Complaints /Discipline	Provincial/ territorial	SK	"Off-duty" conduct—in this case, conduct via social media—may be subject to professional discipline. Recommends regulatory bodies carefully assess and review these complaints in accordance with their governing legislation, codes of conduct, rules, and regulations to determine if their expectations of their members regarding off-duty conduct are transparent to all, including the members.	Canadian nursing regulation is the focus
102	Mackay G, Risk M. Building quality practice settings: An attributes model. Nurs Leadersh. 2001;14(3):19–27.	2001	Discussion paper	N/A	Describes the College of Nurses of Ontario's (CNO) Quality Practice Setting Attributes Model which provides the foundational framework for the College's Practice Setting Consultation Program (PSCP), a component of the Quality Assurance Program.	Continuing competence program	Provincial/ territorial	ON	The Quality Practice Setting Attributes Model provides a foundation for a unique quality improvement approach to creating quality practice environments.	Canadian nursing regulation is the focus
103	MacMillan K. Perspectives on the need for a nursing entry-to-practice examination in Canada. Nurs Leadersh. 2019;32(4):12–6.	2019	Discussion paper	N/A	Explores the objectives and purpose of nursing entry-to-practice (ETP) examinations within the Canadian historical context and presents jurisdictional comparisons.	Registration/ licensure	National	N/A	Suggests that entry-to-practice examinations are only one way of assessing competency for licensure and all options should be considered rather than making policy based on the historical context.	Canadian nursing regulation is included in the broader context
104	MacMillan K, Oulton J, Bard R, Nicklin W. Americanizing Canadian nursing: Nursing regulation drift. HRO-ORS [Internet]. 2017 [cited 2024 May 8];5(3). Available from: https://mulpress.mcmaster.ca/hro-ors/article/view/3154	2017	Discussion paper	N/A	Argues that the adoption of a US-based entry-to-practice exam may have significant consequences for Canadian content and the integrity of education programs, francophone parity in testing, and the future of primary health care and health system reform.	Registration/ Licensure	National	N/A	Canadian nursing regulators have made entry-to-practice examination decisions without key stakeholder consultation that have resulted in lower pass rates on licensing exams for Canadian graduates and may negatively impact nursing human resources, especially for francophone graduates.	Canadian nursing regulation is included in the broader context
105	Maier CB. The role of governance in implementing task-shifting from physicians to nurses in advanced roles in Europe, U.S., Canada, New Zealand and Australia. Health Policy. 2015;119(12):1627–35.	2015	Empirical study	Multi-methods (scoping review and survey)	Examines task-shifting practices with a particular focus on regulatory governance models for advanced practice nurses/nurse practitioners (APN/NPs), based on data from the 2015 International TaskShift2Nurses survey. Explores the implications of different governance models on implementation, patient safety, role clarity, and the availability of workforce statistics.	Regulatory Models, Governance Structures, and Reforms	Global	N/A	Large differences exist across countries in how APN/NPs are governed and regulated. Levels and locus of regulation ranged from no, decentralized to nationwide which had distinct implications on the practice and implementation, role clarity and data availability. Regulation was identified as a barrier if outdated and overtly restrictive or as an enabler to advanced practice, if up to date with competency levels, yet the evidence base is limited.	Canadian nursing regulation is included in the broader context
106	Maritime-wide Registered Psychiatric Nurse Feasibility Study Steering Committee, New Brunswick Regional Advisory Committee, Nova Scotia Regional Advisory Committee, Prince Edward Island Regional Advisory Committee. Registered Psychiatric Nurses in New Brunswick, Nova Scotia and Prince Edward Island: A Feasibility Study [Internet]. 2023 May [cited 2024 May 7]. Available from: https://rpnmaritimesstudy.ca/assets/rpn-maritime-feasibility-studyreport.pdf	2023	Grey literature	Research study report	Outlines the results of a literature review, informant interviews, focus groups, and survey results that aimed to explore the pathway to licensure and regulation of registered psychiatric nurses (RPN) in Nova Scotia, New Brunswick and PEI.	Regulatory Models, Governance Structures, and Reforms	Provincial/ territorial	Multiple (NS, NB, PEI	Results provide the groundwork for the regulation of RPNs in these provinces as well as in other jurisdictions.	Canadian nursing regulation is the focus
107	Martin-Misener R. Will nurse practitioners achieve full integration into the Canadian health-care system? CJNR. 2010;42(2):9–16.	2010	Discussion paper	N/A	Discusses the forces for and against full integration of nurse practitioners (NP) into primary and acute care including legislative/regulatory, education, and practice influences.	Nursing Roles/Standards	National	N/A	The full integration of NPs into primary and acute care would be facilitated by harmonized pan-Canadian legislation and regulation requirements, standardized education, and adequate numbers to increase visibility and measurable contribution.	Canadian nursing regulation is included in the broader context
108	Martin-Misener R, McNab J, Edwards ISS and L. Collaborative practice in health systems change: The Nova Scotia experience with the strengthening primary care initiative. Nurs Leadersh. 2004;17(2):33–45.	2004	Discussion paper	N/A	Describes the implementation and implications of the Strengthening Primary Care Initiative (SPCI) including legislative and regulatory influences on the introduction of nurse practitioners (NP) in the context of interdisciplinary collaboration.	Nursing Roles/Standards	Provincial/ territorial	NS	Collaboration among health disciplines and with community organizations in planning, implementing and evaluating change is essential for success and is influenced by the employment arrangements for the NP and the mechanism of remuneration for family physicians. Collaboration between the Registered Nurses Association of Nova Scotia and the College of Physicians and Surgeons of Nova Scotia supported the expansion of the NP role in primary care; however, legislative challenges persisted. Suggests that the effect of structures and processes embedded in the healthcare system had as significant of an effect on the collaboration between nurse practitioners and family physicians in the SPCI as their characteristics, attitudes and previous experiences.	Canadian nursing regulation is included in the broader context
109	May KA, Singh-Carlson S. The adoption of NCLEX-RN in Canada: A failure of leadership at the intersection of nursing regulation, education and practice. Nurs Leadersh. 2019;32(4):57–65.	2019	Commentary	Includes qualitative analysis of "conversations"	Critiques the level of stakeholder engagement leading up to, and assumptions behind, the decision made by Canadian nurse regulators to adopt the NCLEX-RN entry-to-practice exam.	Registration/ Licensure	National	N/A	Suggests that regulatory leaders failed to involve key leaders and stakeholders from nursing education and practice in the decision to adopt the NCLEX-RN entry-to-practice exam, resulting in negative consequences borne primarily by the education and practice sectors.	Canadian nursing regulation is the focus

110	McGillis Hall L, Jones C, Lalonde M, Strudwick G, McDonald B. Not very welcoming: A survey of internationally educated nurses employed in Canada. GSTF Journal of Nursing and Health Care (JNHC). 2015;2(2):1–6.	2015	Empirical study	Mixed methods (Cross-sectional survey)	Explores the experiences of internationally educated nurses (IENs) who came to Canada to seek nursing work.	Registration/ Licensure	National	N/A	Professional (e.g., salary & benefits, 60%) and personal (e.g., quality of life, 56%) reasons drove migration to Canada, but 76% reported no recruitment incentives, and most (56%) relied on friends and family for information about nursing in Canada. Significant barriers to practicing in Canada included the licensure exam (75%), and obtaining information about different types of practice in Canada (56%).	Canadian nursing regulation is included in the broader context
111	McGillis Hall L, Lalonde M, Kashin J. People are failing! Something needs to be done: Canadian students' experience with the NCLEX-RN. Nurse Education Today. 2016;46:43–9.	2016	Empirical study	Qualitative (thematic analysis of semi-structured interviews)	Explores the experiences of Canadian graduate student nurses who were the first to write the NCLEX examination for entry to practice in Canada to determine whether any implementation issues were identified and how these could be addressed.	Registration/ Licensure	National	N/A	The experiences of study participants with NCLEX implementation in Canada were negative. Thematic findings include: a) temporary test centre concerns, b) perceptions of American context and content on the exam, c) lack of French language resources and translation issues, d) the limited number of opportunities to write the exam, e) communication and engagement with regulators, f) financial costs incurred and g) reputational costs for the Canadian nursing profession.	Canadian nursing regulation is included in the broader context
112	McGillis Hall L, Lalonde M, Kashin J, Yoo C, Moran J. Changing nurse licensing examinations: media analysis and implications of the Canadian experience. International Nursing Review. 2018;65(1):13–23.	2018	Empirical study	Qualitative (content analysis)	Examines the perceptions of the implementation of the NCLEX-RN entry-to-practice exam in Canada through a content analysis of articles in the media.	Registration/ Licensure	National	N/A	Media reports highlight differences in perception of the examination between Canadian regulators and other stakeholders in the context of the examination experiences reported and test results. Issues around the applicability of the examination to Canadian nursing practice, curriculum alignment, language translation concerns and stakeholder engagement were identified.	Canadian nursing regulation is the focus
113	McGillis Hall L, Lalonde M, Visekruna S, Chartrand A, Reali V, Feather J. A comparative analysis of NCLEX pass rates: Nursing health human resources considerations. Journal of Nursing Management. 2019;27(6):1067–74.	2019	Empirical study	(Quantitative) comparative analytic study	Examines the performance differences among different writers of the NCLEX-RN entry-to-practice examination in Canada; compares Canadian and U.S. writer pass rate data; and identifies if changes in the Canadian nursing workforce can be related to the introduction of NCLEX-RN.	Registration/ Licensure	National	N/A	The change in licensing examination had a major impact on pass rates for new graduates entering the nursing profession and potentially the number of new nurses entering the profession in Canada immediately after graduation.	Canadian nursing regulation is included in the broader context
114	McGuire M, Murphy S. The internationally educated nurse. Can Nurse. 2005;101(1):25–9.	2005	Discussion paper	N/A	Describes the demographics of internationally educated nurses (IENs) in Canada and outlines barriers to practice as well as contributions made by IENs.	Registration/ Licensure	National	N/A	IENs in Canada are a demographically-diverse group that contribute to the nursing workforce with retention rates higher than that of domestically trained nurses. IENs face significant barriers related to licensing requirements including the entry-to-practice examination as well as challenges when starting to practice in the Canadian setting related to differences in nursing practice.	Canadian nursing regulation is included in the broader context
115	Melrose S, Kirby D. Mandatory practice hours. Can Nurse. 1996;92(3):51–2.	1996	Commentary	N/A	Critiques the regulatory mandatory practice hours requirement as prescriptive when considering this requirement from a feminist lens.	Continuing competence program	National	N/A	Presents several examples of the personal implications of the mandatory practice hours requirement and encourages the regulator to consider alternatives for competency assessment.	Canadian nursing regulation is the focus
116	Mercier C. The Quebec-France agreement on the mutual recognition of professional qualifications. J Nurse Regul. 2012;2(4):53–7.	2012	Discussion paper	N/A	Describes the mutual recognition of professional qualifications between nurses in Quebec and nurses in France including the political context, conditions of the agreement, adaptation period program, number of nurses affected, and the importance of maintaining professional nursing standards in the international context.	Registration/ Licensure	Global	N/A	The process represents a step in reducing barriers to international nurse migration and encourages continued work toward international regulation or the establishment of universal professional standards.	Canadian nursing regulation is the focus
117	Mildon B. Commentary: Regulation and the nursing profession: A Personal Reflection. Nurs Leadersh. 2018;31(3):34–41.	2018	Commentary	N/A	Offers personal reflections about nursing regulation and the profession, specifically, the evolution of the three pillars of nursing, the emerging changes in regulatory structures and professional associations, and the changing relationship between regulators and registrants.	Regulatory Models, Governance Structures, and Reforms	Provincial/ territorial	ON	Presents examples to critique regulatory and union processes as well as the lack of collaboration between the nursing regulators and the other nursing organizations and calls upon all three nursing pillars, together with policy experts and decision-makers, to collaboratively identify and address the barriers to protection of the public that have been created by various legislations, regulations and collective bargaining jurisprudence.	Canadian nursing regulation is the focus
118	Moghabghab R, Tong A, Hallaran A, Anderson J. The difference between competency and competence: A regulatory perspective. J Nurse Regul. 2018;9(2):54–9.	2018	Empirical study	Qualitative (Concept analysis)	Describes the process used to develop the definitions for competency and competence used by the College of Nurses of Ontario (CNO), as well as implications for regulators.	Nursing Roles/Standards	Provincial/ territorial	ON	Competency is defined as a component of knowledge, skill, and/or judgment, demonstrated by an individual, for safe, ethical, and effective nursing practice. Competence is defined as an individual's capability to consistently integrate the required knowledge, skill, and judgment for safe, ethical, and effective nursing practice.	Canadian nursing regulation is the focus
119	Morrison A, Benton DC. Analyzing nursing regulation worldwide. J Nurse Regul. 2010;1(1):44–7.	2010	Empirical study	Comparative analysis	Describes the risks associated with increased nurse migration and the research objectives developed by the International Council of Nurses' (ICN) Observatory on Licensure and Registration including: developing a global database of nurse regulators and contact details; creating an archive of nursing legislation related to the role, functions, and powers of regulators in each country; conducting a comparative analysis across the key regulatory dimensions of governance, discipline, and education; identifying best practices for each key dimension and a means for sharing this information; and providing a lexicon of key terms. This article summarizes an ICN study that was intended as an initial step toward achieving these objectives.	Regulatory Models, Governance Structures, and Reforms	Global	N/A	Demonstrates the complexity and variability of coverage of nursing regulation around the world and provides recommendations for future action to map legislative and regulatory trends and build and maintain a global database.	Canadian nursing regulation is included in the broader context
120	Mundie C, Donelle L. The environment as a patient: A content analysis of Canadian nursing organizations and regulatory bodies policies on environmental health. Can J Nurs Res. 2022;54(4):464–73.	2022	Empirical study	Qualitative (Policy content analysis)	Explores the policies of Canadian nursing regulatory bodies and associations on nursing practice specific to environmental health.	Nursing Roles/Standards	National	N/A	Identified limited environmental health awareness within nursing practice among competency documents and nursing position statements. Nurse regulatory bodies expect nurses to include sustainable actions within their nursing practice, but do not provide relevant information and tools to successfully implement these measures.	Canadian nursing regulation is included in the broader context

121	Murphy S. Actualizing the dream: Experiences of internationally-educated nurses. [Calgary, Alberta]: University of Calgary; 2008.	2008	Thesis/ Dissertation	Qualitative/ Grounded Theory	Explores the experiences of internationally-educated registered nurses (IENs) as they obtained licensure and employment in Toronto, Ontario.	Registration/ Licensure	Provincial/ territorial	ON	As part of their journey to practice as nurses in Canada, IENs were negated professionally and experienced a lack of support related to interactions with the College of Nurses of Ontario (CNO) and the licensure process.	Canadian nursing regulation is included in the broader context
122	Napolitano M, Roots A, Hoyle C, Johansen C. Developing the clinical licensure examination for nurse practitioners in British Columbia. J Nurse Regul. 2011;2(3):44–8.	2011	Discussion paper	N/A	Describes the College of Registered Nurses of British Columbia's (CRNBC) development and implementation of an objective structured clinical examination (OSCE) based on a framework developed in 2004 for the initial registration of nurse practitioners (NPs).	Registration/ Licensure	Provincial/ territorial	BC	A rigorous process was developed to create a fair, effective OSCE that ensures public safety with purposeful attention to reliability and validity. Through seven offerings, the OSCE and its implementation have been measured as successful. Further outcomes reported by NPs include a sense of pride and accomplishment after passing the OSCE and recognition and credibility from colleagues.	Canadian nursing regulation is the focus
123	National Council of State Boards of Nursing. A global profile of nursing regulation, education, and practice. J Nurse Regul. 2020;10(4):1–116.	2020	Empirical study	Mixed methods (Survey)	Reports data collected among nurse regulators worldwide to develop a global profile of nursing regulation.	Regulatory Models, Governance Structures, and Reforms	Global	N/A	Provides a global review of nursing regulation focused on regulation and governance, licensure and registration requirements to practice, nurse types and titles, education, practice and discipline.	Canadian nursing regulation is included in the broader context
124	Neiterman E, Bourgeault IL. Cultural competence of internationally educated nurses: Assessing problems and finding solutions. CJNR. 2013;45(4):88–107.	2013	Empirical study	Qualitative (Semi-structured interviews)	Examines how the cultural differences in nursing practice impact the process of professional integration for internationally educated nurses (IENs) in Canada.	Registration/ Licensure	Provincial/ territorial	Multiple (BC, MB, ON, QC)	Some IENs find it difficult to understand the registration requirements and accompanying assessment of their level of nursing qualification from their country of training, which often delays the integration process. Language proficiency remains a central challenge for many IENs, including nurses who are native speakers of one of Canada's official languages (English and French) but are unfamiliar with Canadian nursing terminology. The differences in the model of Canadian nursing practice compared to models abroad are a key barrier to IENs passing their exams and becoming successfully integrated into the Canadian healthcare system.	Canadian nursing regulation is included in the broader context
125	Nelson S. Global trends, local impact: The new Era of skilled worker migration and the implications for nursing mobility. Nurs Leadersh. 2013;26(SP):84–8.	2013	Discussion paper	N/A	Discusses the challenges of the federated Canadian healthcare professional regulatory system which limits the mobility of professionals nationally.	Regulatory Models, Governance Structures, and Reforms	National	N/A	Suggests that the barriers to national mobility in Canada and the introduction of the US-based NCLEX-RN entry-to-practice exam for RNs may result in the loss of Canadian-trained RNs to the US.	Canadian nursing regulation is included in the broader context
126	Nelson S, Purkis ME. Mandatory reflection: the Canadian reconstitution of the competent nurse. Nursing Inquiry. 2004;11(4):247–57.	2004	Discussion paper	N/A	Compares how continuing competence is undertaken in Canada, Australia, New Zealand and the United Kingdom and critiques the Canadian nurse regulatory authorities' requirement for reflective practice exercises as a major focus for professional accountability.	Continuing competence program	Global	N/A	The reflective component of the regulatory framework for Canadian nurses fails as a tool for auditing quality and assessing competency as it privileges the 'attribute' element of the nursing competency model and sidesteps the ever-problematic 'nursing knowledge' issue; it shifts the onus for professional development from industry to the individual. It gives up responsibility for skill assessment by the regulatory authorities to industry control, which leaves employers free to determine skill base and skill mix requirements for practice.	Canadian nursing regulation is the focus
127	Nova Scotia College of Nursing Formation Team. Building a new future together: A legislative consultation report for one nursing regulator in Nova Scotia [Internet]. 2017 [cited 2024 May 7]. Available from: /https://www.nscn.ca/sites/default/files/documents/reports/ONR-Consultation-Report-Fall2017.pdf	2017	Grey literature	Consultation report	Presents the findings of a consultation with various stakeholders outlining the strengths and challenges of current provincial regulatory structures and opportunities for effective nursing regulation in the future as multiple nurse regulatory bodies amalgamate in Nova Scotia.	Regulatory Models, Governance Structures, and Reforms	Provincial/ territorial	NS	Findings focus on a need for enhanced transparency, accountability, and consistency in regulatory processes and modernization of the governance structure.	Canadian nursing regulation is the focus
128	Nova Scotia College of Nursing Formation Team. Inspiring the next phase forward: A legislative consultation report for creating one nursing regulator in Nova Scotia [Internet]. 2018 [cited 2024 May 7]. Available from: /https://www.nscn.ca/sites/default/files/documents/reports/Creation-of-ONR-Consultation-Report-Winter-2018.pdf	2018	Grey literature	Consultation report	Presents the findings of a consultation with various stakeholders, outlining recommendations for the proposed regulatory legislation in Nova Scotia.	Regulatory Models, Governance Structures, and Reforms	Provincial/ territorial	NS	Findings focused on the topics of governance, mandate and accountability, professional conduct, registration and licensure, and scope of practice.	Canadian nursing regulation is the focus
129	Nova Scotia College of Nursing Formation Team. From concept to creation: A legislative consultation report for one nursing regulator in Nova Scotia [Internet]. 2019 [cited 2024 May 7]. Available from: /https://www.nscn.ca/sites/default/files/documents/reports/Jan-2019-Consultation-Report.pdf	2019	Grey literature	Consultation report	Presents the findings of a consultation with various stakeholders, outlining responses to proposed governance changes at the amalgamated nurse regulatory body in Nova Scotia.	Regulatory Models, Governance Structures, and Reforms	Provincial/ territorial	NS	Findings reported that the majority of stakeholders support the incorporation of proposed procedures for processing and addressing settlement agreements currently found in the RN and LPN regulations and the process to be used by the Reinstatement Committee with reviewing an application.	Canadian nursing regulation is the focus
130	Nova Scotia College of Nursing Formation Team. Governance building blocks: A by-law consultation report for one nursing regulator in Nova Scotia [Internet]. 2019 [cited 2024 May 7]. Available from: /https://cdn1.nscn.ca/sites/default/files/documents/reports/By-Laws_Consultation_Report.pdf	2019	Grey literature	Consultation report	Presents the findings of a consultation with various stakeholders, outlining responses to new bylaws proposed for the amalgted nurse regulatory body in Nova Scotia.	Regulatory Models, Governance Structures, and Reforms	Provincial/ territorial	NS	The majority of respondents supported the regulator's proposed bylaws.	Canadian nursing regulation is the focus

131	Ogilvie L, Leung B, Gushuliak T, McGuire M, Burgess-Pinto E. Licensure of internationally educated nurses seeking professional careers in the province of Alberta in Canada. Int Migration & Integration. 2007;8(2):223–41.	2007	Discussion paper	N/A	Describes nurse licensure practices in Canada using Alberta as an example. Initiatives to remove barriers and facilitate success, tensions associated with internationally educated nurses (IENs) licensure in the Canadian context, and directions for practices, policies, and research that may enhance IEN integration into professional nursing roles in Canada are described.	Registration/ Licensure	Provincial/ territorial	AB	Provides policy recommendations promoting facility in the English language, ease of verification of documents, equivalency in terms of educational levels both on entry to nursing education and completion of programs, and comparability of nursing education curricula and clinical practice settings which are likely to influence access to licensure positively.	Canadian nursing regulation is included in the broader context
132	Peacock J, Douglas A, Duplessis K, Hamilton C, Smith C, Harper S. Virtual care permit program in Canada. J Nurse Regul. 2024;14(4):24–9.	2024	Discussion paper	N/A	Describes the process by which the College of Registered Nurses of Alberta (CRNA) collaborated with the College of Registered Nurses of Saskatchewan (CRNS) to develop a memorandum of agreement (MOA) to facilitate and expedite the registration of registered nurses and nurse practitioners for the provision of virtual care across provincial borders.	Registration/ Licensure	Provincial/ territorial	multiple (AB, SK, NWT, NU)	Addresses key regulatory requirements included in the MOA (including registration, licensing, continuing competence, professional liability insurance, complaints and discipline, and information sharing) for the provision of inter-jurisdictional virtual care services. This pilot program enabled both regulators to adopt a common regulatory framework while ensuring that quality of care, accountability, and protection of the public were not compromised and lessons learned from the pilot were provided.	Canadian nursing regulation is the focus
133	Penney C, Bayne L, Johansen C. Developing a relational regulatory philosophy on a public protection mandate. J Nurse Regul. 2014;5(3):44–7.	2014	Discussion paper	N/A	Describes the process that the College of Registered Nurses of British Columbia (CRNBC) undertook to develop and implement a regulatory philosophy to help the organization set priorities, align programs and services with its regulatory mandate and communicate changes to nurse registrants and stakeholders.	Regulatory Models, Governance Structures, and Reforms	Provincial/ territorial	BC	The development of a philosophy has helped CRNBC reflect on its history, understand stakeholders' perspectives, and determine how to move forward as a strong, effective, and valued regulatory body.	Canadian nursing regulation is the focus
134	Pepin JI, Myrick F. The current state of Canada's health care system and the potential contribution of nursing education to strengthen it: An interview with the Chief Nursing Officer Dr. Leigh Chapman. Quality Advancement in Nursing Education - Avancées en formation infirmière [Internet]. 2023 [cited 2024 Apr 17];9(1). Available from: https://qane-afi.casn.ca/journal/vol9/iss1/8	2023	Commentary	Interview	Describes Chief Nursing Officer Dr. Leigh Chapman's opinions on the current challenges in the Canadian health care system, the implications for nursing educatio, and the potential contribution of nursing education to strengthen the system.	Regulatory Models, Governance Structures, and Reforms	National	N/A	Describes the challenges related to the fragmented regulation of nurses due to federated legislative jurisdictions and the individual regulation of each nursing designation across Canada which limits the unification of the profession in collaborating effectively with stakeholders. Highlights the need for educators to focus on streamlining education programs for internationally educated nurses to better prepare IENs to meet the health human resource needs across Canada. Priorities for action include: accelerating the integration of internationally educated nurses (IENs), advancing multi-jurisdictional registration, improving the portability of licensure, improving our data, as well as nursing retention and engagement.	Canadian nursing regulation is included in the broader context
135	Pesut B, Thorne S, Stager ML, Schiller CJ, Penney C, Hoffman C, et al. Medical Assistance in Dying: A review of Canadian nursing regulatory documents. Policy Polit Nurs Pract. 2019;20(3):113–30.	2019	Empirical study	Multi-methods (Web-based search and qualitative descriptive approach)	Analyzes the documents created by Canadian nursing regulatory bodies to support registered nurses(RNs) and nurse practitioners (NPs) in the practice of medical assistance in dying (MAiD).	Nursing Roles/Standards	National	N/A	Nursing regulators across Canada have done an admirable job of providing guidance to nurses about MAiD through the documents but much remains to address system-wide issues and promote good nursing practice in the public interest while supporting the needs of nurses amidst this complex moral and legal landscape.	Canadian nursing regulation is the focus
136	Petrovic K, Doyle E, Lane A, Corcoran L. The work of preparing Canadian nurses for a licensure exam originating from the USA: A nurse educator's journey into the institutional organization of the NCLEX-RN. Int J Nurs Educ Scholarsh. 2019;16(1).	2019	Empirical study	Qualitative (Institutional ethonography lens)	Presents the experience of a Canadian nurse educator working to facilitate students' transition from a Canadian-developed, owned and delivered exam to the National Council Licensure Examination for Registered Nurses (NCLEX-RN) which originates from the United States.	Registration/ Licensure	National	NA	Shares strategies to manage the change while continuing to listen to and heed the voices of students.	Canadian nursing regulation is included in the broader context
137	Phillips LA, Weis J. Education program standards of Canadian practical nurse programs. J Nurse Regul. 2018;9(1):38–46.	2018	Empirical study	Enviornmental scan	Presents the results of a jurisdictional scan of the Canadian Practical Nurse Regulators' (CCPNR) education program review standards and identifies opportunities and barriers to a national practical nurse education standard.	Nursing Education Approval and Accreditation	National	N/A	CCPNR guiding documents can, and should, be used as a foundation for practical nursing (PN) educational outcomes, curriculum, and program design. The development and use of a standardized PN program approval framework can strengthen inter-jurisdictional trust in academic programming. If adopted across jurisdictions, a program approval framework would contribute to enhanced PN educational programming, creating a stronger foundation for the profession and safer practice throughout Canada.	Canadian nursing regulation is the focus
138	Prentice D, Moore J, Crawford J, Lankshear S, Limoges J. Collaboration among Registered Nurses and Licensed Practical Nurses: A scoping review of practice guidelines. Nurs Res Pract. 2020:1–7.	2020	Empirical study	Qualitative (reports on regulatory guidelines previously identified in a scoping review)	Examines ten nursing practice guidelines from various Canadian nurse regulatory authorities that inform the registered nurse (RN) and registered/licensed practical nurse (R/LPN) collaborative practice in acute care.	Nursing Roles/Standards	National	N/A	The findings indicate that many of the guidelines were not evidence-informed. Although the guidelines discussed the structures needed to support intraprofessional collaboration, and most of the guidelines mention that quality patient care is the desired outcome of intraprofessional collaboration, outcome indicators for measuring successful collaborative practice were missing in many of the guidelines. Conflict resolution was only mentioned in a few of the guidelines despite being an important process component of collaborative practice. Future guidelines should be evidence-informed and provide outcome indicators to measure if collaborative practice is occurring in the practice setting.	Canadian nursing regulation is the focus
139	Professional Standards Authority. A review conducted for the College of Registered Nurses of British Columbia [Internet]. 2016 Apr [cited 2024 May 8]. Available from: /https://www.professionalstandards.org.uk/docs/default-source/publications/special-review-report/a-review-conducted-for-the-college-of-registered-nurses-of-british-columbia-(april-2015).pdf?sfvrsn=49db7120_14	2016	Grey literature	Regulatory review/audit	A comprehensive review of the College of Registered Nurses of British Columbia's (CRNBC) performance as a regulator against the Professional Standards Authority's "Standards of Good Regulation".	Regulatory Models, Governance Structures, and Reforms	Provincial/ territorial	BC	Provides recommendations that focus on guidance and standards documents, education program review and continuing competence, registration, and complaints processes at CRNBC.	Canadian nursing regulation is the focus

140	Professional Standards Authority. A review conducted for the Saskatchewan Registered Nurses Association [Internet]. 2019 May [cited 2024 May 8]. Available from: /https://www.professionalstandards.org.uk/docs/default-source/publications/international-reports/a-review-conducted-for-the-saskatchewan-registered-nurses-association-(may-2019).pdf?sfvrsn=d6a07420_7	2019	Grey literature	Regulatory review/audit	Review of the Saskatchewan Registered Nurses Association's (SRNA) complaints, investigations, and discipline processes against the Professional Standards Authority's "Standards of Good Regulation".	Conduct/Complaints /Discipline	Provincial/ territorial	SK	Provides several specific recommendations to align complaints processes with the Standards of Good Regulation.	Canadian nursing regulation is the focus
141	Puckrin KM. Building confidence in the self-regulation of nurses [Internet] [M.A.]. [British Columbia, CA]: Royal Roads University; 2006 [cited 2024 Apr 17]. Available from: https://www.proquest.com/docview/304910292/abstract/A5D9EDFAEDEE434APQ/1	2006	Thesis/ Dissertation	Qualitative (Action research)	Applies the principles of program evaluation to design a systematic assessment of the College of Nurses of Ontario's (CNO) Participative Resolution Program (PRP). Describes the development of a logic model of the program which links the PRP with the College's strategic plan, specifies what the program intends to accomplish, and how these outcomes will be achieved.	Conduct/Complaints/Discipline	Provincial/ territorial	ON	The recommendations emerging from this project suggest that a paradigm shift away from adversarial investigations towards the collaborative resolutions of complaints by the parties through PRP is desirable.	Canadian nursing regulation is the focus
142	Puddester R, Limoges J, Dewell S, Maddigan J, Carlsson L, Pike A. The Canadian landscape of genetics and genomics in nursing: A policy document analysis. Can J Nurs Res. 2023;55(4):494–509.	2023	Empirical study	Qualitative (document analysis)	Examines the guidance for genetics and genomics-informed nursing practice as provided by Canadian nursing organizations in official professional documents.	Roles/scopes of practice	National	All provinces	There is an overall lack of depth and breadth of Canadian nursing documents that include content related to genetics and genomics (GG). Opportunities exist to enhance the guidance available to Canadian nurses for the application of GG, through documents of nursing professional associations, nursing education accreditation organizations, and regulatory bodies.	Canadian nursing regulation is included in the broader context
143	Pulcini J, Jelic M, Gul R, Loke AY. An international survey on advanced practice nursing education, practice, and regulation. J Nurs Scholarsh. 2010;42(1):31–9.	2010	Empirical study	Mixed methods (Cross-sectional, descriptive, web-based survey)	Describes international trends on the developing role of nurse practioner-advanced practice nurse (NP-APN), including nomenclature, levels and types of education, practice setting, scope of practice, regulatory policies, and political environment.	Roles/scopes of practice	Global	N/A	NP-APN roles are expanding globally and the greatest support comes from domestic nursing organizations, individual nurses, government, while opposition primarily comes from domestic physician organizations and individual physicians.	Canadian nursing regulation is included in the broader context
144	Quinn MJ. An examination of various aspects of continuing education with an emphasis upon the motivational orientations of registered nurses in Ontario [Internet] [Ed.D.]. [Ontario, CA]: University of Toronto; 1995 [cited 2024 Apr 17]. Available from: https://www.proquest.com/docview/304209102/abstract/2F3C9800E4794463PQ/1	1997	Thesis/ Dissertation	Mixed methods (structured questionnaire and interviews)	Describes the current participation in continuing education of the registered nurses in Ontario including the continuing learning of registered nurses in Ontario; the types of educational programs they report, and the topics and activities that are of greatest interest; the kinds of providers they choose; the reasons why they participate (or do not) in continuing education; whether participation or nonparticipation in continuing education is related to the respondents' demographic characteristics; and their attitudes toward the Registered Nurses Association; and to assess attitudes toward the possible implementation of mandatory continuing education by the College of Nurses of Ontario (CNO).	Continuing competence program	Provincial/ territorial	ON	Provides analysis on the reported types of educational programs, the topics and activities that are of greatest interest, the kinds of providers respondents choose, and the reasons why they participate (or do not) in continuing education. The respondents' attitudes toward the Registered Nurses Association of Ontario (RNAO) and the issue of mandatory continuing education with the College of Nurses of Ontario (CNO) for re-licensure were also examined.	Canadian nursing regulation is included in the broader context
145	Registered Psychiatric Nurse Regulators of Canada. Mobility and assessment of Canadian and internationally educated Registered Psychiatric Nurses [Internet]. 2015 Apr [cited 2024 May 7]. Available from: /https://www.rpnc.ca/sites/default/files/resources/pdfs/RPNRC-prjct-rpt-FINAL-PMC.pdf	2015	Grey literature	Research study report	Describes the process and outcomes of a literature review, consultations with stakeholders, and a survey aimed at addressing the assessment and integration of internationally educated registered psychiatric nurses (RPNs) in Canada.	Registration/ Licensure	National	N/A	Recommendations focus on reducing barriers to mobility and licensure of internationally educated RPNs, increasing the availability of tools to support foreign qualification recognition, and greater coordination and collaboration between Canadian nursing regulators.	Canadian nursing regulation is the focus
146	Reid DA. A prospective policy analysis of the elimination of the exclusive scope of practice from the Nursing Profession Act [Internet]. [Alberta, Canada]: Univeristy of Alberta; 1999. Available from: https://www.collectionscanada.gc.ca/obj/s4/f2/dsk3/ftp04/nq39615.pdf	1999	Thesis/ Dissertation	Qualitative (case study/policy analysis)	Determines if eliminating the exclusive practice clause from the Nursing Profession Act in Alberta is a feasible policy solution to the perceived need to eliminate barriers to the provision of professional services, or if other solutions are more desirable or feasible.	Regulatory Models, Governance Structures, and Reforms	Provincial/ territorial	AB	The elimination of exclusive scopes of practice from health profession legislation in Alberta is a significant policy issue. The existing model of health profession legislation characterized by exclusive professional jurisdictions and scopes of practice is perceived by some nursing leaders as an achievement of professional status but is not in keeping with the contemporary need for more flexible scopes of practice which recognize that different types of competent practitioners may provide the same	Canadian nursing regulation is the focus
147	Rodgers SJ. The role of nursing theory in standards of practice: A Canadian perspective. Nurs Sci Q. 2000;13(3):260–2.	2000	Discussion paper	NA	Discussed the role of nursing theory in standards of practice provided by the College of Nurses of Ontario (CNO).	Nursing Roles/Standards	Provincial/ territorial	ON	Nursing theories have played a significant role in shaping and influencing the profession of nursing, and this influence becomes evident when comparing and contrasting the focus and content of standards of practice over the past 25 years.	Canadian nursing regulation is the focus
148	Roots A. The international nurse regulator collaborative mobility project: Transjurisdictional mobility—Is it possible? J Nurse Regul. 2023;13(4):21–32.	2023	Empirical study	Mixed methods (multi-phased, multiple-case study design)	Investigated the possibility of recognizing existing licensure or registration thereby reducing barriers and allowing for more streamlined mobility between International Nurse Regulator Collaborative (INRC) jurisdictions.	Registration/ Licensure	Global	N/A	A high level of consistency was found in the expectations, standards, and operational processes across jurisdictions. Challenges existed in relation to requirements for and assessments of educational qualifications despite entry-to-practice competencies and educational program approval requirements being largely the same. Jurisdictional regulators work under different legislative frameworks, which may potentially create different challenges and timelines for moving toward trans-jurisdictional mobility.	Canadian nursing regulation is the focus

149	Rozovsky LE. Should the nurse's role be defined in law? Dimensions in health service. 1976;53(3):9–11.	1976	Discussion paper	N/A	Discusses the nuances and implications of defining the role of the nurse in legislation and the resulting regulations related to registration, licensure, and protection of title.	Regulatory Models, Governance Structures, and Reforms	National	N/A	Compares various types of nursing profession legislation and the difficulty of legalizing a role that is difficult to define. Nursing is a profession that may benefit from legislation because they are required to rely upon their professional judgment even in the context of employed work where patient care is ordered by a physician.	Canadian nursing regulation is the focus
150	Salami B, Meherali S, Covell CL. Downward occupational mobility of baccalaureate-prepared, internationally educated nurses to licensed practical nurses. Int Nurs Rev. 2018;65(2):173–81.	2018	Empirical study	Qualitative (exploratory transnational feminsit qualitative design)	Explores the experience of baccalaureate-prepared, internationally educated nurses (IENs) who work or have worked as licensed practical nurses (LPNs) in Canada.	Registration/ Licensure	National	N/A	Results revealed four key themes related to the experiences of this group of nurses: they migrate to Canada with the hope of a better personal and professional life; they experience barriers to workforce integration as registered nurses (RN) and discover an easier path in the LPN registration process; they experience deskilling and ambivalent skill recognition; and they feel dissatisfied as an LPN in Canada. Implications for policymakers include the need to address the barriers to becoming RNs, including application processing times and lack of adequate access to educational programmes.	Canadian nursing regulation is included in the broader context
151	Salfi J, Carbol B. The applicability of the NCLEX-RN to the Canadian testing population: A review of regulatory body evidence. Int J Nurs Educ Scholarsh. 2017;14(1):109–30.	2017	Discussion paper	N/A	Examines whether the two National Council of State Boards of Nursing (NCSBN) studies, referenced by Canadian regulators as part of the justification for the adoption of the NCLEX- RN exam, provided sufficient evidence to conclude that the exam is appropriate for Canadian populations. Determines whether the two studies establish that the NCLEX-RN examination would provide a fair, valid, and psychometrically sound measurement of the minimal nursing competencies required for safe and effective practice for those seeking registration or licensure as a registered nurse (RN) in Canada.	Registration/ licensure	National	N/A	While some evidence was found of the use of best practice principles in survey and research design, both authors call into question the evidence provided by the NCSBN and deny the claims that the NCLEX-RN, as currently designed, is an appropriate assessment tool for Canadian entry-level nurses	Canadian nursing regulation is included in the broader context
152	Santa Mina EE, Bhatti A, Bradley P, Manafo E, Ormiston A, Patrick L, et al. University competency-based courses for internationally educated nurses (IENs) in Ontario: A pilot education pathway to Registered Nurse (RN) licensure. Quality Advancement in Nursing Education - Avancées en formation infirmière [Internet]. 2023 [cited 2024 Apr 19];9(1). Available from: https://qane-afi.casn.ca/journal/vol9/iss1/2	2023	Discussion paper	NA	Describes the Ontario Internationally Educated Nurses (IENs) Course Consortium's process to create foundational learning and competency-based courses to meet targeted entry-to-practice (ETP) competencies for registered nurse (RN) registration with the College of Nurses of Ontario (CNO). It describes the barriers that IENs face in meeting ETP requirements, the gap in the existing Ontario IEN bridging to BScN education to meet regulatory requirements, the innovative courses and approaches to address these needs, and the program of study outcomes.	Registration/ Licensure	Provincial/ territorial	ON	A concept-based and competency-based curriculum has been used to develop the program of study, the aim which is to increase the number of IENs who can meet the CNO requirements for RN practice in Ontario, including clinical practice hours, and to further expand this program's reach and capacity to meet the needs of IENs.	Canadian nursing regulation is included in the broader context
153	Scanlon A, Bryant-Lukosius D, Lehwaldt D, Wilkinson J, Honig J. International transferability of nurse practitioner credentials in five countries. The Journal for Nurse Practitioners. 2019;15(7):487–93.	2019	Discussion paper	NA	Describes the background and regulatory requirements for nurse practitioners (NPs) in 5 countries, Australia, Canada, Ireland, New Zealand, and the United States, and explores the process that internationally educated nurse practitioners (IENPs) must follow to obtain authority to practice in any of these countries. Recommendations for improving the regulation of IENPs to facilitate the mobility of the global NP workforce are provided.	Nursing Roles/Standards	Global	N/A	The variation of education, credentials and scope of practice of IENPs impedes, and in some instances prevents, international transferability, as country- and even state/province/territory-specific training is not always directly transferable. Nursing organizations and governments at the state and federal levels need to determine how best to address NP mobility. Recommends standardizing international requirements for appropriately qualified IENPs.	Canadian nursing regulation is included in the broader context
154	Schreiber R, Davidson H, MacDonald M, Crickmore J, Moss L, Pinelli J, et al. Advanced nursing practice: Opportunities and challenges in British Columbia [Internet]. Canadian Health Services Research Foundation; 2003. Available from: https://www.researchgate.net/publication/228557549_Advanced_Nursing_Practice_Opportunities_and_Challenges_in_British_Columbia	2003	Grey literature	Research study report	Explores the potential contribution of advanced registered nursing practice within the British Columbia health care context.	Nursing Roles/Standards	Provincial/ territorial	BC	Recommendations pertaining to regulatory legislation, practice models, and title protection.	Canadian nursing regulation is the focus
155	Seabrook EM. A search for role clarity: A critical discourse analysis of the RN and RPN entry-to-practice competencies that shape nursing curriculum in Ontario, Canada [Internet] [Ph.D.]. [Ontario, CA]: The University of Western Ontario; 2023 [cited 2024 Apr 19]. Available from: https://www.proquest.com/docview/2866357113/abstract/B28BA29384B14B09PQ/1	2023	Thesis/ Dissertation	Qualitative (Critical discourse analysis)	Explores the meaning and intent of the entry-to-practice competencies provided by the College of Nurses of Ontario (CNO), the differences in practice expectations for registered nurse (RN) versus registered practical nurses (RPN) graduates, and explores how can role clarity be improved through this process.	Roles/scopes of practice	Provincial/ territorial	ON	Some language use and sentence construction within the entry-to-practice documents confound even a seasoned educator as these words have different meanings depending on the context and common understanding of the meaning. A summary table of the findings illustrates the differences and similarities and serves as a guide to aid role clarity for educators, employers, nurses, nursing students and the public.	Canadian nursing regulation is the focus
156	Sellman D. From CRNE to NCLEX-RN: Musings on nursing and the idea of a national final examination. Nurs Philos. 2016;17(4):227–8.	2016	Commentary	NA	Discusses the adoption of the entry-to-practice (ETP) NCLEX-RN exam in Canada and critiques the exclusion of content related to the health care system, cultural issues, and the legislation of Canada and questions the purpose of an ETP examination given existing regulatory processes for approving nursing education programs.	Registration/ licensure	National	N/A	Critiques the adoption of the NCLEX-RN exam and questions the need for a standardized entry-to-practice examination to assess the competence of nurses when nurse regulators accredit education programs.	Canadian nursing regulation is the focus

157	Shaffer FA, Robinson MA, Dutka JT, Tuttas C. A new model for assessing entry-level rducation of internationally educated nurses: A retrospective perspective. J Nurse Regul. 2016;6(4):51–7.	2016	Discussion paper	N/A	Describes the formation of the National Nursing Assessment Service (NNAS), an entity that supports regulatory bodies in providing fair, valid, and consistent assessments of internationally educated nurses (IENs) who apply to be registered in Canadian jurisdictions.	Registration/ Licensure	National	N/A	The partnership between the NNAS and the Commission on Graduates of Foreign Nursing Schools (CGFNS) has provided a conceptual framework, a model, and a system for credential assessment for the nursing profession and beyond.	Canadian nursing regulation is the focus
158	Sheer B, Wong FKY. The development of advanced nursing practice globally. J Nurs Scholarsh. 2008;40(3):204–11.	2008	Empirical study	Qualitative (survey)	Examines the development of advanced nursing practice globally.	Nursing Roles/Standards	Global	N/A	Some nations begin with the role and then develop the title, scope, and regulation. Other nations begin with regulation and move on to educational programs and development of the role. Advanced nursing throughout the world is related to the perceived status of nursing and women, the need for healthcare services, existing health policies and resources, and the ratio of physicians to nurses. Although the evolution of APNs differs in each nation, similarities exist.	Canadian nursing regulation is included in the broader context
159	Simmonds AH. Leadership, education and awareness: A compassionate care nursing initiative. Nurs Leadersh. 2015;28(1):53–64.	2015	Empirical study	Qualitative (intervention study)	Describes a compassionate care initiative taken by the College of Registered Nurses of Nova Scotia (CRNNS) and the initial outcomes of this initiative.	Nursing Roles/Standards	Provincial/ territorial	NS	Educational initiatives such as those undertaken by CRNNS may not resolve all concerns related to compassionate care delivery, but they do have the potential to contribute to building the capacity of nursing leaders and front-line nursing staff to reflect on and develop strategies for enhancing the delivery of quality, safe and compassionate care.	Canadian nursing regulation is the focus
160	Singh-Carlson S, May KA. Adoption of NCLEX-RN for licensure in Canada: Faculty concerns and implications for nursing education. J Prof Nurs. 2020;36(2):77–82.	2020	Discussion paper	N/A	Critiques the Canadian nurse regulators' adoption of the US-based entry to practice NCLEX examination.	Registration/ Licensure	National	N/A	Suggests international adoption of a US-based examination for initial licensure merits wider discussion by nursing faculty in Canada and abroad and advocates for the establishment of a unique national licensing exam which better reflects the Canadian healthcare context.	Canadian nursing regulation is included in the broader context
161	Singh MD, Sochan A. Voices of internationally educated nurses: Policy recommendations for credentialing. Int Nurs Rev. 2010;57(1):56–63.	2010	Empirical study	Qualitative (biographical narrative research)	Explore how credentialing processes can be made more transparent, standardized, and harmonized between international professional regulatory bodies and their national immigration processing institutions based on the experiences of internationally educated nurses (IENs) in Ontario.	Registration/ Licensure	Provincial/ territorial	ON	Offers recommendations based on the personal experiences of IENs, all of which have policy implications to improve the transparency, standardization and harmonization of the credentialing processes both prior to, and upon arrival in their destination country. The national immigration agencies and nursing regulatory bodies could better coordinate their activities when processing potential IEN migrant applications.	Canadian nursing regulation is the focus
162	Sochan A, Singh MD. Acculturation and socialization: Voices of internationally educated nurses in Ontario. Int Nurs Rev. 2007;54(2):130–6.	2007	Empirical study	Qualitative (biographical narrative)	Describes the experiences of internationally educated nurses (IENs) in their efforts to gain entry to practice as registered nurses in Ontario, highlighting issues related to professional nursing credentialing.	Registration/ Licensure	Provincial/ territorial	ON	Findings include perceived experiences of personal, financial, and cultural injustices related to a bureaucratic credentialing process that is not readily understood by the IENs, that is, those for whom the process was established. Professional regulatory nursing bodies and nursing educators, as well as practising nurses, must be aware of the potentially confusing and unpleasant processes IENs go through as they qualify for the privilege of practising nursing in Ontario.	Canadian nursing regulation is included in the broader context
163	Stahlke Wall S. The impact of regulatory perspectives and practices on professional innovation in nursing. Nurs Inq. 2018;25(1):1–8.	2018	Empirical study	Qualitative (qualitative descriptive)	Explores the impact that regulatory processes have on innovation in nursing roles.	Regulatory Models, Governance Structures, and Reforms	Provincial/ territorial	Anonymized	The nurses in this study fully respected the need for regulatory monitoring and standards but were frustrated with their experiences with the regulatory body as they sought licensure for their non-traditional practices, recognition of their education, and approval of cross-jurisdictional career experience. They perceived that their regulator had a rigid and traditional perspective on nursing practice and they experienced frustrating and costly delays as they worked through the confusing process of gaining licensure.	Canadian nursing regulation is the focus
164	Stanhope-Goodman S, Hendrickson B, Nordstrom P. Completing substantially equivalent competency assessment: Barriers and facilitators for internationally educated nurses. J Nurse Regul. 2014;5(1):35–9.	2014	Empirical study	(Mixed methods) self-adminsitered web-based questionnaire	Discusses the Substantially Equivalent Competency (SEC) assessment for internationally educated nurses (IENs) with insufficient documentation for a determination of their eligibility for registered nurse licensure in Albert. Explores the barriers and facilitators that influenced the IENs throughout the assessment process.	Registration/ Licensure	Provincial/ territorial	AB	The results indicate that IENs who do not complete the assessment process experience difficulties obtaining travel documents and extended time delays. IENs who completed the assessment process encountered different barriers and facilitators, depending on their stage in the assessment process. The results of the study provide suggestions and recommendations that regulators and assessment service providers can consider when developing competency assessment processes.	Canadian nursing regulation is included in the broader context
165	Staples E. The tension between regulation and the pursuit of quality in Canadian nurse practitioner education programs. Policy Polit Nurs Pract. 2022;23(1):41–7.	2022	Discussion paper	N/A	Describes the current fragmented approach to accredit nurse practitioner (NP) education programs in Canada and initiates a discussion between regulators and educators related to proposed regulatory approaches and accreditation processes that balance public safety while promoting quality and excellence in NP education.	Nursing Education Approval and Accreditation	National	N/A	An integrated pan-Canadian regulatory approval and accreditation process may be the country’s best approach to ensure continuity and consistency in education, regulation, mobility of the NP workforce, and systematic planning to guide future NP role development and practice.	Canadian nursing regulation is the focus
166	Steering Committee on Modernization of Health Professional Regulation. Recommendations to modernize the provincial health profession regulatory framework [Internet]. Government of British Columbia; 2020 [cited 2024 May 7]. Available from: /https://www2.gov.bc.ca/assets/gov/health/practitioner-pro/professional-regulation/recommendations-to-modernize-regulatory-framework.pdf	2020	Grey literature	Regulatory review/audit	Reports on how the College of Dental Surgeons and British Columbia's (CDSBC) overall professional regulatory network, including nurses, can modernize to improve patient safety and public protection, improve efficiency and effectiveness of the regulatory framework, and increase public confidence through transparency and accountability.	Regulatory Models, Governance Structures, and Reforms	Provincial/ territorial	BC	Recommendations for regulatory modernization include: commitment to cultural safety and humility, improved governance, reduction of the number of colleges, and strengthening oversight of regulatory colleges.	Canadian nursing regulation is included in the broader context
167	Stewart G, Strachan A. Sustaining an occupation-specific language assessment for the Canadian healthcare field. TESL Canada. 2021;38(1):49–66.	2021	Discussion paper	N/A	Describe the development, implementation, renewal, and maintenance of the Canadian English Language Benchmark Assessment for Nurses (CELBAN) which has been accepted as evidence of language ability for licensure of internationally educated nurses (IENs) in Canada and discuss the complexities of sustaining an occupation-specific assessment over time.	Registration/ Licensure	National	N/A	As the future of occupation-specific language assessment unfolds, and as circumstances in the Canadian healthcare context continue to evolve over time, it is hoped that the CELBAN will have the resilience to go on serving the needs of IENs and nursing regulators for years to come.	Canadian nursing regulation is the focus

168	Tarlier DS, Browne AJ. Remote nursing certified practice: Viewing nursing and nurse practitioner practice through a social justice lens. Can J Nurs Res. 2011;43(2):38–61.	2011	Discussion paper	N/A	Discusses the implications of regulating a new category of registered nurse in British Columbia (BC), the Remote Nursing Certified Practice (RNCP). From a critical social justice lens, RNCP obscures the need for nurse practitioners (NP) in remote and Indigenous communities and perpetuates inequitable access to high-quality primary care experienced in these areas. Explores the historical and current role of the NP in BC and the advent of the RNCP role, comparing their scopes of practice.	Regulatory Models, Governance Structures, and Reforms	Provincial/ territorial	BC	The authors conclude by calling for nursing regulations that support equitable, high-quality primary care for all British Columbians.	Canadian nursing regulation is included in the broader context
169	Tarnowski GJ, Bateman T, Stanger L, Phillips LA. Update of licensed practical nurse competencies in Alberta. J Nurse Regul. 2017;8(2):17–22.	2017	Empirical study	Multi-methods (surveys and consultation)	Describes the College of Licensed Practice Nurses of Alberta's (CLPNA) development of the competency profile of Alberta's licensed practical nurses (LPNs) in 1998, the need for updates in 2005, and the most recent process to update the 2015 edition of the competency profile.	Nursing Roles/Standards	Provincial/ territorial	AB	The third edition of the Competency Profile for Licensed Practical Nurses of Alberta reflects the educational preparedness, role, and scope of practice of the Alberta LPN profession in 2015. The profile is intended to be a living document and will evolve with the progression of LPN practice. The process used to update the document may provide suggestions and provoke discussion in the regulatory community regarding how competency updates can be managed given the pace of change in the evolving health care environment.	Canadian nursing regulation is the focus
170	Thiessen NJ, Leslie K, Stephens JML. An examination of self-employed nursing regulation in three Canadian provinces. Policy Polit Nurs Pract. 2023;24(4):265–77.	2023	Empirical study	(Qualitative) Case study	Examines the regulation of self-employed nurses by comparing the regulatory policies and processes of nursing regulatory bodies in Ontario, Alberta, and Saskatchewan.	Nursing Roles/Standards	Provincial/ territorial	Multiple (ON, AB, SK)	The regulation of self-employed RNs and NPs varies significantly across the studied jurisdictions and that, in Alberta and Saskatchewan, self-employed nurses must complete additional requirements to be licensed to practice. In some cases, the information provided by the regulator regarding these additional processes is incomplete and unclear, potentially creating barriers for nurses in these roles and eroding public trust in the regulation of self-employed nursing practice. When nurses opt for self-employment, it is essential to ensure that the public has access to safe and high-quality nursing care, highlighting the need for evidence-informed regulation of independent nursing practice roles.	Canadian nursing regulation is the focus
171	Tilley E, Devion C, Coghlan AL, McCarthy K. A regulatory response to healthcare Serial killing. J Nurse Regul. 2019;10(1):4–14.	2019	Discussion paper	Literature review (*not a systematic review)	Discusses the College of Nurses of Ontario's (CNO) efforts to understand the healthcare serial killer phenomenon based on a criminal case (R v Wettlaufer, 2017) in Ontario and describes common factors associated with healthcare serial killers, their victims, their crimes, and possible detection based on the literature.	Regulatory Models, Governance Structures, and Reforms	Global	N/A	Provides recommendations for actions that regulators can take in collaboration with stakeholders such as employers and other agencies. In working with other stakeholders, CNO hopes it can help prevent a similar tragedy by shining a spotlight on potential risks and engaging all stakeholders in the system as partners in ensuring patient safety.	Canadian nursing regulation is the focus
172	Tilley E, Hamilton-Jones M, McNabb A. Nurse practitioners' safe prescribing of controlled substances and the impact on nursing education in Ontario. J Nurse Regul. 2019;9(4):42–7.	2019	Empirical study	Mixed methods (Surveys and literature review)	Describes the regulatory changes the College of Nurses of Ontario (CNO) implemented to support safe practice, focusing on the specific elements added to controlled substances education as well as discussing practice resources for nurse practitioners (NP). It also describes findings from a survey that sought to understand the impact of NPs prescribing controlled substances in Ontario and what NPs needed to support their practice.	Nursing Roles/Standards	Provincial/ territorial	ON	Describes the College's approach to supporting initial and ongoing competence for NPs to safely prescribe controlled substances, with a particular focus on education. It also highlights outcomes and feedback received approximately 4 months after implementation to inform future work.	Canadian nursing regulation is the focus
173	Tirana I, McCarthy K. The College of Nurses of Ontario's governance vision: Using evidence to transform regulatory governance in the public interest. J Nurse Regul. 2020;11(3):49–56.	2020	Discussion paper	N/A	Describes the College of Nurses of Ontario's (CNO) decision to undertake a governance review and the ensuing development of a vision for a progressive model of regulatory governance that puts the public first.	Regulatory Models, Governance Structures, and Reforms	Provincial/ territorial	ON	The CNO has begun implementation of their vision for modernizing its governance structure in the absence of legislative change. The CNO continues to engage the government to make changes to the paradigm of regulatory governance in Ontario while building support with other healthcare regulators for governance reform.	Canadian nursing regulation is the focus
174	Tisdale D, Symenuk PM. Human rights and nursing codes of ethics in Canada 1953–2017. Nurs Ethics. 2020;27(4):1077–88.	2020	Empirical study	Qualitative (Historical method)	Examines continuity and changes to human rights in the nursing codes of ethics between the years 1953 and 2017, which spans the very first code in Canada to the most recently adopted.	Nursing Roles/Standards	National	N/A	Findings suggest there has been very little change in how human rights have been included within the Canadian nursing codes of ethics which may change as regulators shift to exclusively public interest mandates, distancing themselves from the CNA and the associated widely-recognized nursing code of ethics. Describes the increasing influence of regulators in ensuring the protection of human rights is included in authoritative nursing standards.	Canadian nursing regulation is the focus
175	Tregunno D, Peters S, Campbell H, Gordon S. International nurse migration U-turn for safe workplace transition. Nurs Inq. 2009;16(3):182–90.	2009	Empirical study	Qualitative (Semi-structured interviews)	Examines the barriers and challenges internationally educated nurses (IENs) experience transitioning into the Ontario workforces after they achieve initial registration between 2003-2005.	Registration/ Licensure	Provincial/ territorial	ON	Found IENs may paradoxically be both expert clinicians and novices in culture and language fluency at the same time. Recommendations focus on cultural orientation through education and social acculturation through preceptorship and informal social networks.	Canadian nursing regulation is included in the broader context
176	Trimblett SM. Gamification in nursing jurisprudence [Internet] [M.A.]. [British Columbia, CA]: Royal Roads University; 2016 [cited 2024 Apr 19]. Available from: https://www.proquest.com/docview/1816978616/abstract/2C1D4C5D8D8D4B09PQ/1	2016	Thesis/ Dissertation	Mixed methods (interviews and surveys)	Evaluates the College of Licensed Practical Nurses of British Columbia's (CLPNBC) Jurisprudence Pilot Program that used gamified learning to teach LPNs about jurisprudence. Explores whether LPNs identified any influence to change their attitudes and behaviours regarding how jurisprudence informs their practice.	Registration/ Licensure	Provincial/territorial	BC	There are three different elements of gamification that can lead to engagement: immersion, intrinsic and extrinsic motivation and competition. LPNs, when asked if they were able to influence change in their workplace after completing JPP, seemed divided - LPNs felt that JPP had an influence on their attitude and behaviours in relation to regulation and standards of practice, but stated they were unable to influence change in their workplace. The gamified education was successful in teaching LPNs what jurisprudence means to their nursing practice.	Canadian nursing regulation is the focus

177	Tuden D, Secong D, Wainwright A. Nurse practitioner regulatory assessment: Transitioning from an onsite to a virtual format. Comput Inform Nurs. 2023;41(12):968–74.	2023	Discussion paper	N/A	Describes the process by which the British Columbia College of Nurses and Midwives (BCCNM) transitioned from an onsite to a virtual nurse practitioner (NP)regulatory assessment as part of the organization's Quality Assurance program.	Continuing competence program	Provincial/ territorial	BC	Discuss the organization's experience, including benefits, technical and administrative considerations, barriers, challenges, and lessons learned related to the transition from onsite to virtual NP regulatory assessments.	Canadian nursing regulation is the focus
178	Twohig PL. The second "Great Transformation": Renegotiating nursing practice in Ontario, 1945–70. Canadian Historical Review. 2018;99(2):169–95.	2018	Empirical study	Qualitative (Historical method)	Examines the introduction of registered nursing assistants in Ontario and the negotiation of nursing practice between various nursing groups. Describes how the system of registration created in Ontario effectively legitimated the participation of registered nurses (RNs) in the regulation, governance, education, and supervision of nursing assistants.	Regulatory Models, Governance Structures, and Reforms	Provincial/ territorial	ON	RNs effectively managed the issue of encroachment by registered nursing assistants, limiting their autonomy, through involvement in the regulation, governance, education, and supervision of nursing assistants.	Canadian nursing regulation is included in the broader context
179	Van Kleef J, Werquin P. PLAR in nursing: Implications of situated learning, communities of practice and consequential transition theories for recognition. Int Migration & Integration. 2013;14(4):651–69.	2013	Empirical study	Qualitative (case study)	Explores the meaning and effectiveness of prior learning assessment recognition (PLAR) as a means to accredit internationally educated nurses (IENs) in Ontario based on interviews with PLAR candidates. Reveals differences in nursing education and professional practice between PLAR candidates and the regulatory requirements in Ontario.	Registration/ Licensure	Provincial/ territorial	ON	IENs encounter significant barriers in receiving recognition for their prior learning related to a lack of effective collaboration between the educator providing PLAR services and the provincial nurse regulator.	Canadian nursing regulation is included in the broader context
180	Villeneuve, M. J. (2020). Medical Assistance in Dying: A Review of Canadian Regulatory Documents. Policy, Politics, & Nursing Practice, 21(2), 56–59. https://doi.org/10.1177/1527154420923733	2020	Commentary	N/A	Describes the events that led up to the legalization of medical assistance in dying (MAiD) in Canada in 2016 from the perspective of the Canadian Nurses Association (CNA) and congratulates the efforts of nurse regulators in responding quickly and collaboratively to ensure the safe and effective participation of registered nurses in the delivery of this service. Describes the complexities and barriers created by the federated structure which leads to duplication and a confusing lack of harmonization in the delivery of MAiD across provincial and territorial jurisdictions.	Nursing Roles/Standards	National	N/A	Opines that the intervention of nurses during the development of the MAiD legislation and nurse regulators' rapid and collaborative creation of standards, guidelines, and educational tools serves as an exemplar to other jurisdictions.	Canadian nursing regulation is the focus
181	Villeneuve MJ. Medical Assistance in Dying: A review of Canadian regulatory documents. Policy Polit Nurs Pract. 2020;21(2):56–9.	2019	Discussion paper	N/A	Highlights pivotal events in the evolution of Canadian nursing regulation and the Canadian Nurses Association's (CNA) role in developing the first national standard competency exam for nurses. Stresses the need for regulators, educators, professional nurses, and unions to work collaboratively to ensure the assessment of graduate nurses is effective and fair, and ensures the safety of the public.	Registration/ licensure	National	N/A	Describes the CNA's role in creating and delivering the Canadian Registered Nursing Exam (CRNE) for 45 years prior to the CCRNR's decision to move to the NCLEX-RN. Commits as an organization to work closely with all pillars of professional nursing to strengthen the profession now that the CNA's role has shifted away from all regulatory responsibilities.	Canadian nursing regulation is included in the broader context
182	Vincelette C, Audet LA, Fortin O, Dumont M, Robillard N. Cardiopulmonary resuscitation certification requirements for Canadian registered nurses: A pressing need for standardization. The Canadian Journal of Critical Care Nursing. 2019;30(3):8–12.	2019	empirical study	Mixed methods (Cross-sectional survey)	Explores the cardiopulmonary resuscitation (CPR) certification requirements of all Canadian provinces and territories for intensive care and general ward registered nurses (RNs), whether regulatory bodies require proof of certification, and identifies future opportunities for research and practice.	Continuing competence program	National	N/A	Most of the respondents (91%) reported that RNs are not required to provide proof of either BLS or ACLS certification to their board to renew licensure. Furthermore, RN regulatory bodies suggest it is the responsibility of RNs to maintain their CPR skills current and for hospitals to regulate CPR certification requirements.	Canadian nursing regulation is the focus
183	Wainwright A, Klein T, Daly C. Competency development to support safe nurse practitioner prescribing of controlled drugs and substances in British Columbia. Policy Polit Nurs Pract. 2016;17(3):125–35.	2016	Empirical study	Quantitative (competency development frmaework)	Describe the development and refinement of controlled drugs and substances prescribing competencies and the policy process for their implementation in educating and regulating nurse practitioners (NP) after legislation was passed in Canada to authorize the prescription of controlled drugs and substances.	Nursing Roles/Standards	Provincial/ territorial	BC	Full implementation of the competencies took longer than anticipated at the outset of the policy work due to a commitment to safe implementation, the complexity and interconnectivity of the work involving multiple stakeholders, and a growing commitment to create a national framework for NP practice.	Canadian nursing regulation is the focus
184	Walton-Roberts M. Intermediaries and transnational regimes of skill: Nursing skills and competencies in the context of international migration. Journal of Ethnic & Migration Studies. 2021;47(10):2323–40.	2021	Empirical study	Multimethods (draws on empirical data from a multi-sited research project - interview and focus groups)	Explores the practice of private migration intermediaries, who are utilized by internationally educated nurses to navigate the processes of immigration and licensure abroad, and the concept of a "regime of skills" which created and is maintained through interactional and transnational connections between various stakeholders including intermediaries, educators, and regulators. Demonstrates how migration intermediaries operate transnationally, in the sending and recieving countries, integrating themselves into the process of skill construction, codification, testing and regulation, maximizing their market share and providing services to an ever larger market of internationally-educated nurses.	Registration/ Licensure	Global	N/A	The state controls the policy pathways for migration purposes, and the professional regulators control the "regime of skill", or the opportunity for internationally educated nurses (IEN) in Canada, including the credential evaluation and testing processes. Migration intermediaries deeply integrate themselves into the process of skill construction, codification, testing and regulation transnationally and thus have market interest in both the sending and receiving countries. Other actors in this network react to the forces being imposed, but in some cases, they can co-construct and exploit the opportunities created by distinct regimes of skill.	Canadian nursing regulation is included in the broader context
185	Wearing J, Nickerson V. Establishing a regulatory framework for certified practices in British Columbia. J Nurse Regul. 2010;1(3):38–43.	2010	Discussion paper	N/A	Describes the development of a new regulatory approach, called Certified Practice, created by the College of Registered Nurses of British Columbia (CRNBC) in response to concerns identified with mechanisms of delegation and indirect orders.	Nursing Roles/Standards	Provincial/ territorial	BC	The Certified Practice approach allowed the CRNBC to appropriately regulate expanded registered nursing practice.	Canadian nursing regulation is the focus
186	Wearing J, Black J, Kline K. A model for nurse practitioner regulation: Principles underpinning a three-registration category approach. Nurs Leadersh. 2010;22(4):40–9.	2010	Discussion paper	N/A	Describes the processes by which the College of Registered Nurses of British Columbia (CRNBC) decided to register only three categories of NP: family/all ages, adult and pediatrics.	Regulatory Models, Governance Structures, and Reforms	Provincial/ territorial	BC	CRNBC was confident that the three-registration category approach to the regulation of nurse practitioners met the obligation to protect the public by ensuring that NPs have broad knowledge and skills to meet the common healthcare needs of the population with whom they work.	Canadian nursing regulation is the focus

187	Wheeler KJ, Miller M, Pulcini J, Gray D, Ladd E, Rayens MK. Advanced practice nursing roles, regulation, education, and practice: A global study. Ann Glob Health [Internet]. 2022 [cited 2024 May 8];88(1). Available from: https://annalsofglobalhealth.org/article/10.5334/aogh.3698/	2022	Empirical study	(Mixed methods) Survey	Compares the role of the Advance Practice Nurse (APN) across 26 countries, outlining APN scopes of practice, education requirements, regulation, and practice climates globally.	Nursing Roles/Standards	Global	N/A	There remain inconsistencies in APN titling, title protection, regulation, and credentialing, among other barriers to APN practice globally which inhibits the potential positive impact of this role around the world.	Canadian nursing regulation is included in the broader context
188	Williams DL. External audit: Final report - CRNM compliance with principles for quality assurance and continuing competence [Internet]. Dundee Consulting Group Ltd.; 2019 Nov [cited 2024 May 1]. Available from: https://www.crnm.mb.ca/	2019	Grey literature	Regulatory review/audit	Presents the results of an external audit of the College of Registered Nurses of Manitoba's (CRNM) quality assurance/continuing competence processes to measure the extent with which they align to the CRNM's regulatory principles.	Continuing competence program	Provincial/ territorial	MB	Recommendations for further compliance with the Executive Expectations are provided.	Canadian nursing regulation is the focus
189	Williams DL. External audit: Final report - CRNM compliance with registration processes principles [Internet]. Dundee Consulting Group Ltd.; 2020 Nov [cited 2024 May 1]. Available from: https://www.crnm.mb.ca/	2020	Grey literature	Regulatory review/audit	Presents the results of an external audit of the College of Registered Nurses of Manitoba's (CRNM) registration processes to measure the extent to which they align with the CRNM's regulatory principles.	Registration/ Licensure	Provincial/ territorial	MB	Recommendations for further compliance with the Executive Expectations are provided.	Canadian nursing regulation is the focus
190	Williams DL. External audit report - CRNM compliance with EE-12 Executive expectations: Professional conduct [Internet]. Dundee Consulting Group Ltd.; 2022 [cited 2024 May 1]. Available from: https://www.crnm.mb.ca/	2022	Grey literature	Regulatory review/audit	Presents the results of an external audit of the College of Registered Nurses of Manitoba's (CRNM) professional conduct processes to measure the extent to which they align with the CRNM's regulatory principles.	Conduct/Complaints /Discipline	Provincial/ territorial	MB	Recommendations for further compliance with the Executive Expectations are provided.	Canadian nursing regulation is the focus
191	Williams DL. External audit: Phase 2 monitoring report - CRNM compliance with registration processes principles [Internet]. Dundee Consulting Group Ltd.; 2022 Feb [cited 2024 May 1]. Available from: https://www.crnm.mb.ca/	2022	Grey literature	Regulatory review/audit	Presents the results of an external audit of the College of Registered Nurses of Manitoba's (CRNM) registration requirements and practice competence to measure the extent to which they align to the CRNM's regulatory principles.	Registration/ Licensure	Provincial/ territorial	MB	Recommendations for further compliance with the Executive Expectations are provided.	Canadian nursing regulation is the focus
192	Woodend K. Transition to NCLEX-RN: Reflections from a CASN past president. Nurs Leadersh. 2019;32(4):22–9.	2019	Editorial	N/A	Describes the editor's experience as president of Canadian Association of Schools of Nursing (CASN) while the Canadian Council of Registered Nurses Regulators (CCRNR) transitioned from the CRNE to the NCLEX-RN as the mandatory standardized competency exam for entry-level registered nurses in Canada.	Registration/ Licensure	National	N/A	Describes CASN's efforts to advocate for Canadian, particularly francophone, nursing students against the regulators' decision to move to the American-sourced, English NCLEX-RN exam and the associated lack of Canadian preparatory resources.	Canadian nursing regulation is included in the broader context
193	Worster A, Sardo A, Thrasher C, Fernandes C, Chemeris E. Understanding the role of nurse practitioners in Canada. Can J Rural Med. 2005;10(2):89–94.	2005	Discussion paper	N/A	Describes the role of the nurse practitioner (NP) in Canada inclusive of a historical overview, definition, education, and functions of the NP. Additionally, discusses legislative, regulatory, and medicolegal issues relating to the NP role in Canada and presents evidence related to the role's potential benefit to the health care system.	Nursing Roles/Standards	National	N/A	At the time of writing, a perceived lack of a concerted and collaborative effort by Canadian nurse regulators to create universal accreditation and licensure standards for NPs was a significant barrier to their effective inclusion in the national healthcare strategic plan.	Canadian nursing regulation is included in the broader context
194	Xu Y. A Comparison of regulatory standards for initial registration/licensure of internationally educated nurses in the United Kingdom, Australia, Canada, and the United States. J Nurse Regul. 2011;2(3):27–36.	2011	Discussion paper	N/A	Describes and compares regulatory standards for registration/licensure of internationally educated nurses (IENs) in the United Kingdom, Australia, Canada, and the United States.	Registration/ Licensure	Global	N/A	The four countries share many regulatory requirements for the registration/licensure of IENs. The most noticeable difference is whether international nurse applicants must go through a tailored transition program.	Canadian nursing regulation is included in the broader context
195	Zelisko D, Baumann A, Gamble B, Laporte A, Deber R. Ensuring accountability through health professional regulatory bodies: The case of conflict of interest. Healthc Policy. 2014;10(SP):110–20.	2014	Empirical study	Qualitative (descriptive document analysis)	Examines how four Ontario regulatory colleges (including physicians, nurses, physiotherapists, audiologist/speech-language pathologists) defined financial conflict of interest (COI) and the education, guidance, and enforcement, they provided for COI.	Nursing Roles/Standards	Provincial/ territorial	ON	These colleges uphold the mandates to define, identify and address financial COI by providing regulations or standards and guidelines to their membership, but differ in the amount of educational materials provided to their registrants and in the possible COI scenarios they presented.	Canadian nursing regulation is included in the broader context

**Abbreviations used in this table:**
NWT (Northwest Territories); NU (Nunavut); AB (Alberta); BC (British Columbia); SK (Saskatchewan); MB (Manitoba); ON (Ontario); QC (Quebec); NB (New Brunswick); NS (Nova Scotia);NL (Newfoundland); PEI (Prince Edward Island)
NP (Nurse practitioner); RN (registered nurse); RPN (Registered practical nurse); LPN (Licensed practical nurse); CNS (Clinical nurse specialist); IEN (Internationally educated nurse)
NA (Not applicable)
