# Supplementary material for: Nursing regulation in Canada: Insights from a scoping review
Source: PLoS One. 2025 May 16;20(5):e0323716. doi: 10.1371/journal.pone.0323716 (PMC12084052; doi:10.1371/journal.pone.0323716)
Supplement: S5 Appendix — (PDF) [file pone.0323716.s005.pdf]

S5 Appendix: Sources Excluded Following Full–Text Review

1. Adlersberg M. Is it within the scope of RN practice or is it delegation? Nurs BC. 2006;38(3):19.
Reason for exclusion: Wrong source

2. Alameddine M, Battershill C, Baumann A, Boon M, Born K, Cortinois A, et al. The demanding supply: licensing international doctors and nurses in Ontario. In: Deber RB, Mah CL, editors. Case studies in Canadian health policy and management [Internet]. University of Toronto Press; 2014. p. 231–60. Available from: https://doi.org/10.3138/9781442609969
Reason for exclusion: Wrong source

3. Alameddine M, Baumann A, Onate K, Deber R. Career transitions of inactive nurses: a registration database analysis (1993–2006). Int J Nurs Stud. 2011;48(2):184–92.
Reason for exclusion: Wrong concept

4. Alberta Association of Registered Nurses. "101" questions and answers about your AARN registration renewal. AARN News Lett. 1997;53(7):15–8.
Reason for exclusion: Wrong source

5. Canadian Nurses Association. 2015 snapshot of regulated nurses. Can Nurse. 2016 Sep;112(6):11. Available from: https://www.canadian–nurse.com/viewdocument/september–2016?CommunityKey=d320003f–c6d8–4209–a9e7–4fbff8be6289&tab=librarydocuments
Reason for exclusion: Wrong concept

6. Conditions of employment of nurses in Canada: regulation in Quebec. Int Labour Rev. 1944;50(4):529–30.
Reason for exclusion: Wrong concept

7. Newfoundland nurses investigate high rate of registration failures. Can Hosp. 1965;42:29–29.
Reason for exclusion: Wrong concept

8. Canadian Nurses Association. [Editorial on Canadian testing services for licensing of professional graduate nurses]. Can Nurse. 1967 May;63(5):27. Available from: https://www.canadian–nurse.com/viewdocument/may–1967?CommunityKey=d320003f–c6d8–4209–a9e7–4fbff8be6289&tab=librarydocuments
Reason for exclusion: Wrong concept

9. Alberta Association of Registered Nurses. A.A.R.N. beliefs re co–ordinating council. AARN News Lett. 1970;6(3):22–4.
Reason for exclusion: Wrong source

10. Registered Nurses' Association of British Columbia. RNABC position statement on continuing education for re–registration. RNABC News. 1974;6(6):15.
Reason for exclusion: Wrong source

11. Registered Nurses' Association of British Columbia. What happens if a nurse isn't safe to practice? RNABC News. 1976;8(1):4–6.
Reason for exclusion: Wrong source

12. Nursing in Canada: Canadian nursing statistics 1975. Abstracts of Hospital Management Studies. 1977;14(2):17625.
Reason for exclusion: Wrong concept

13. College of Nurses of Ontario. Comprehensive examination for nurse registration/licensure. RNAO News. 1978;34(1):6–7.
Reason for exclusion: Wrong source

14. Registered Nurses' Association of British Columbia. Two–year progress report: safety to practice reviewed. RNABC News. 1978;10(8):3–5.
Reason for exclusion: Wrong source

15. Clinical experience proposed for Ontario nurse graduates. AORN J. 1979 Jun;29(7):1364. doi:10.1016/S0001–2092(07)64273–2
Reason for exclusion: Wrong concept

16. Registered Nurses' Association of British Columbia. New registration exam policy: let's clear up the confusion. RNABC News. 1983;15(5):12–3.
Reason for exclusion: Wrong source

17. Registered Nurses' Association of British Columbia. Nurse licensure gains importance. RNABC News. 1984;16(2):14–5.
Reason for exclusion: Wrong source

18. Alberta Association of Registered Nurses. Nursing Profession Act, regulations, bylaws. AARN News Lett. 1984;40(3):suppl–42.
Reason for exclusion: Wrong source

19. Registered Nurses' Association of British Columbia. Support growing for legislation project. RNABC News. 1985;17(4):12–3.
Reason for exclusion: Wrong source

20. Registered Nurses' Association of British Columbia. Changing the Act: proposals for graduate nurses. RNABC News. 1986;18(1):31–2.
Reason for exclusion: Wrong source

21. Registered Nurses' Association of British Columbia. Changing the Act: will I qualify? RNABC News. 1986;18(6):26.
Reason for exclusion: Wrong source

22. Alberta Association of Registered Nurses. Questions and answers on nursing practice hours. AARN News Lett. 1994;50(11):5–6.
Reason for exclusion: Wrong source

23. Alberta Association of Registered Nurses. AARN response to licensed practical nurse regulations. AARN News Lett. 1997;53(6):14–25.
Reason for exclusion: Wrong source

24. Alberta Association of Registered Nurses. Registered nurses gather throughout Alberta to offer insight into continued competence monitoring. AARN News Lett. 1997;53(7):19–22.
Reason for exclusion: Wrong source

25. Alberta Association of Registered Nurses. Registered nurses working on ambulances: amended staff, vehicle, and equipment regulation. AARN News Lett. 1997;53(9):15.
Reason for exclusion: Wrong source

26. Ad Hoc Committee on Competence Assurance. Special report: competence assurance for registered nurses. ConceRN. 1997 Aug;26(4):12–6.
Reason for exclusion: Wrong source

27. Alberta Association of Registered Nurses. A continued competence framework for Alberta's registered nurses. AARN News Lett. 1998;54(7):12–3.
Reason for exclusion: Wrong source

28. Registered Nurses Association of British Columbia. Overview of the Nurses (Registered) Act, rules and RNABC constitution and bylaws. Nurs BC. 1998;30(3):suppl–4.
Reason for exclusion: Wrong source

29. Alberta Association of Registered Nurses. RN registration—answers to commonly asked questions to guide you through the registration renewal ritual. AARN News Lett. 1998;54(7):8–11.
Reason for exclusion: Wrong source

30. Health Professions Act—what are the gains for the nursing profession?. Alta RN. 1999;55(4):4.
Reason for exclusion: Wrong source

31. Registered Nurses Association of British Columbia. Nursing practice guideline. Nurs BC. 1999;31(1):suppl–4.
Reason for exclusion: Wrong source

32. Collaborative nursing practice in Alberta. Alta RN. 2003 Jun;59(11):suppl–9.
Reason for exclusion: Wrong source

33. Registered Nurses Association of British Columbia. Nurse practitioners: BC's newest providers of health care services. Nurs BC. 2004 Jun;36(3):5–7.
Reason for exclusion: Wrong source

34. AARN reproaches authors for flawed research and unfair criticism of AARN continuing competence program. Alta RN. 2005;61(5):8–9.
Reason for exclusion: Wrong source

35. Registered Nurses Association of British Columbia. RNABC submission on proposed regulation for registered nursing. Nurs BC. 2005;37(2):28–9.
Reason for exclusion: Wrong source

36. Canadian Nurses Association. Canadian nurse practitioner initiative: final report urges consistency in licensing nurse practitioners. Can Nurse. 2006;102(7):17.
Reason for exclusion: Wrong concept

37. Canadian Nurses Association. Highlights from the 2005 Canadian Nurses Association annual report. Can Nurse. 2006;102(5):24–33.
Reason for exclusion: Wrong concept

38. Review of decisions of professional conduct and appeals committees. Alta RN. 2006;62(4):7.
Reason for exclusion: Wrong source

39. Registered Nurses Association of British Columbia. Entry–level education. Nurs BC. 2007;39(5):15–6.
Reason for exclusion: Wrong source

40. Registered Nurses Association of British Columbia. Professional conduct review process. Nurs BC. 2007;39(2):11–5.
Reason for exclusion: Wrong source

41. Registered Nurses Association of British Columbia. Appropriate use of titles by nurses. Nurs BC. 2008;40(1):2–28.
Reason for exclusion: Wrong source

42. Registered Nurses Association of British Columbia. Continuing competence for nurse practitioners. Nurs BC. 2008;40(1):19.
Reason for exclusion: Wrong source

43. The challenge of getting internationally educated nurses to the front line. Alta RN. 2008;64(5):10–1.
Reason for exclusion: Wrong source

44. Nursing by the numbers. Alta RN. 2010;66(1):8–13.
Reason for exclusion: Wrong source

45. Regulated nurses in Canada: what do we know about them? Alta RN. 2011;67(5):10–1.
Reason for exclusion: Wrong source

46. Learning from experience project: improving the process of internationally educated nurses' applications for registration. Alta RN. 2012;68(3):6–7.
Reason for exclusion: Wrong source

47. Canadian Nurses Association. Report highlights: regulated nurses, 2013. Can Nurse. 2014;110(6):8.
Reason for exclusion: Wrong concept

48. New harmonized application process for internationally educated nurses. Can Nurse. 2015;111(8):11.
Reason for exclusion: Wrong concept

49. Atack L, Cruz EV, Maher J, Murphy S. Internationally educated nurses' experiences with an integrated bridge program. Journal of Continuing Education in Nursing. 2012;43(8):370–8.
Reason for exclusion: Wrong concept

50. Bailey J. President's message. Nursing in Focus. 2012;13(1):1–2.
Reason for exclusion: Wrong source

51. Baker C. Promoting quality in nursing education in Canada through a Canadian examination for baccalaureate nurses. Nurs Leadersh. 2019;32(4):81–91. doi:10.12927/cjnl.2020.26098
Reason for exclusion: Wrong concept

52. Balaski B, Klenk M, Chow S. RN(NP) update: delegation of medical functions. SRNA News Bull. 2004 Apr–May;6(2):7.
Reason for exclusion: Wrong source

53. Baldachhino G, Saunders P. Internationally educated health professionals in Nova Scotia and Prince Edward Island: why they come, why they stay and the challenges they face: a follow up study. Charlottetown (PE): IEHP Atlantic Connection; 2010 Oct. Available from: https://islandstudies.com/files/2016/12/IEHPI–Oct–2010–Final.pdf
Reason for exclusion: Wrong source

54. Baldwin S, Cheng L. Internationally educated nurses and the Canadian English language benchmark assessment for nurses: a qualitative test validation study of test–taker accounts. Can J Appl Linguist. 2020;23(2):96–117. doi:10.37213/cjal.2020.30435
Reason for exclusion: Wrong concept

55. Balneaves LG, Alraja A, Ziemianski D, McCuaig F, Ware M. A national needs assessment of Canadian nurse practitioners regarding cannabis for therapeutic purposes. Cannabis Cannabinoid Res. 2018;3(1):66–73. doi:10.1089/can.2018.0002
Reason for exclusion: Wrong concept

56. Barry J, Sweatman L, Little L, Davies J. International nurse applicants. Can Nurse. 2003 Sep;99(8):34–5. Available from: https://www.canadian–nurse.com/viewdocument/september–2003?CommunityKey=d320003f–c6d8–4209–a9e7–4fbff8be6289&tab=librarydocuments
Reason for exclusion: Wrong concept

57. Baumann A, Blythe J, McIntosh K, Rhéaume A. Internationally educated nurses in Ontario: maximizing the brain gain. Hamilton (ON): Nursing Health Services Research Unit, McMaster University; 2006. Available from: https://coilink.org/20.500.12592/sjhs49
Reason for exclusion: Wrong source

58. Beechinor LAV, Fitzpatrick JJ. Demands of immigration among nurses from Canada and the Philippines. Int J Nurs Pract. 2008;14:178–87.
Reason for exclusion: Wrong source

59. Béland G. The CNA connection: the testing service: how it works! Can Nurse. 1982;78(4):6–10. Available from: https://www.canadian–nurse.com/viewdocument/april–1982?CommunityKey=d320003f–c6d8–4209–a9e7–4fbff8be6289&tab=librarydocuments
Reason for exclusion: Wrong population

60. Belita LL, Ford PA. Reducing barriers and achieving success in registration examination among internationally educated nurses: a participatory action research project. Can J Nurs Res. 2021;53(4):353–65. doi:10.1177/0844562120985124
Reason for exclusion: Wrong concept

61. Benton DC, Brenton AS, Johnson P, Stansfield K, Benson PS. A thematic analysis of existing sunrise provisions: challenges, findings, and best practices. J Nurs Regul. 2021;12(3):S1–52. doi:10.1016/S2155–8256(21)00125–3

Reason for exclusion: Wrong concept

62. Benton DC, Alexander M, Fotsch R. Lessons learned and insights gained: a regulatory analysis of the impacts, challenges, and responses to COVID–19. Online J Issues Nurs. 2020;25(3). doi:10.3912/OJIN.Vol25No03PPT51
Reason for exclusion: Wrong population

63. Benton DC, González–Jurado MA, Beneit–Montesinos JV. A typology of professional nurse regulatory models and their administration. J Nurs Regul. 2013;4(2):22–9. doi:10.1016/S2155–8256(15)30153–8
Reason for exclusion: Wrong concept

64. Beswetherick MA. What does "RN" after your name really mean? Can Nurse. 1972;68(10):27–8. Available from: https://www.canadian–nurse.com/viewdocument/october–1972?CommunityKey=d320003f–c6d8–4209–a9e7–4fbff8be6289&tab=librarydocuments
Reason for exclusion: Wrong concept

65. Bieski T. Foreign–educated nurses: an overview of migration and credentialing issues. Nurs Econ. 2007;25(1):20–23.
Reason for exclusion: Wrong source

66. Blais N. Cross–Canada registration. Can Nurse. 1976 Jan;72(1):22–5. Available from: https://www.canadian–nurse.com/viewdocument/january–1976?CommunityKey=d320003f–c6d8–4209–a9e7–4fbff8be6289&tab=librarydocuments
Reason for exclusion: Wrong concept

67. Blythe J, Baumann A. Internationally educated nurses: profiling workforce diversity. Int Nurs Rev. 2009;56(2):191–7. doi:10.1111/j.1466–7657.2008.00699.x
Reason for exclusion: Wrong concept

68. Blythe J, Baumann A, Rheaume A, McIntosh K. Nurse migration to Canada: pathways and pitfalls of workforce integration. J Transcult Nurs. 2009;20:202–10. doi:10.1177/1043659608330349
Reason for exclusion: Duplicate

69. Bourgeault IL. Delivering the 'new' Canadian midwifery: the impact on midwifery of integration into the Ontario health care system. Sociol Health Illn. 2000;22(2):172–96. doi:10.1111/1467–9566.00198
Reason for exclusion: Wrong concept

70. Bourgeault IL, Neiterman E, Lebrun J. Midwives on the move: comparing the requirements for practice and integration contexts for internationally educated midwives in

Canada with the U.S., U.K. and Australia. Midwifery. 2011;27(3):368–75. doi:10.1016/j.midw.2011.03.010
Reason for exclusion: Wrong population

71. Braham J, Ward S. Competencies. The foundation for future nursing exams. Can Nurse. 1993 Feb;89(2):39–41. Available from: https://www.canadian–nurse.com/viewdocument/february–1989?CommunityKey=d320003f–c6d8–4209–a9e7–4fbff8be6289&tab=librarydocuments
Reason for exclusion: Wrong concept

72. Briant NJ. What every reasonable and prudent nurse should know. Can Nurse. 1977 Jun;73(6):13. Available from: https://www.canadian–nurse.com/viewdocument/june–1977?CommunityKey=d320003f–c6d8–4209–a9e7–4fbff8be6289&tab=librarydocuments
Reason for exclusion: Wrong concept

73. Brideau N. Can we talk? the nursing profession and self–regulation. Info Nursing. 2001;32(3):5.
Reason for exclusion: Wrong source

74. Bristow L, Campbell J, Crook J, Francis M, Horrocks J, Muise M. Position paper: expanded role of the nurse. Nurs Pap. 1974 Apr;6(2):30–1. Available from https://cjnr.archive.mcgill.ca/article/view/508
Reason for exclusion: Wrong concept

75. Brown DM. Preparation for practice: an evaluation of a pre–registration bachelor of nursing program. 1997.
Reason for exclusion: Wrong concept

76. Brown SA, Grimes DE. A meta–analysis of nurse practitioner and nurse midwives in primary care. Nursing Research. 2005;44, 332–339.
Reason for exclusion: Wrong concept

77. Brown SJ. A framework for advanced practice nursing. J Prof Nurs. 1998;14(3):157–64.
Reason for exclusion: Wrong population

78. Brunke L. On reflection: reshaping nursing regulation. Nurs BC. 2003;35(1):38.
Reason for exclusion: Wrong source

79. Brunke L. A commitment to public protection. Nurs BC. 2004;36(3):38–7.
Reason for exclusion: Wrong source

80. Brunke L. Regulating registered nurses in the public interest. Nurs BC. 2005;37(3):24–7.
Reason for exclusion: Wrong source

81. Brunke L. Providing leadership in a new regulatory world. Nurs BC. 2007;39(3):26–8.
Reason for exclusion: Wrong source

82. Brunke L. Registration in the public interest. Nurs BC. 2007;39(2):38.
Reason for exclusion: Wrong source

83. Brunke L. A legal duty. Nurs BC. 2008;40(2):38.
Reason for exclusion: Wrong source

84. Brunskill D. Licence to practise: is this really an important issue? ConceRN. 1992;21(1):18–9.
Reason for exclusion: Wrong source

85. Brunskill D. London conference on professional regulation impacts nursing. ConceRN. 1995;24(6):18–9.
Reason for exclusion: Wrong source

86. Bryant–Lukosius D, DiCenso A. A framework for the introduction and evaluation of advanced practice nursing roles. J Adv Nurs. 2004;48(5):530–540. doi:10.1111/j.1365-2648.2004.03235.x
Reason for exclusion: Wrong concept

87. Bryant–Lukosius D, DiCenso A, Browne G, Pinelli J. Advanced practice nursing roles: Development, implementation and evaluation. J Adv Nurs. 2004;48(5):519—529. doi:10.1111/j.1365-2648.2004.03234.x
Reason for exclusion: Wrong concept

88. Bullough B. Professionalization of nurse practitioners. In: Fitzpatrick JJ, Stevenson JS, editors. Annual review of nursing research. 1995;13:239–65.
Reason for exclusion: Wrong source

89. Busby N. OHN certification. Can Nurse. 1992;88(9):8–15.
Reason for exclusion: Wrong concept

90. Campbell T, Penz K, Vandenberg H, Campbell M. A closer look at the introduction of the NCLEX–RN in Canada. Can J Nurs Leadersh. 2019;32(4):46–56. doi:10.12927/cjnl.2020.26102
Reason for exclusion: Wrong concept

91. Campbell T, Penz K, Vandenberg H, Campbell M. A closer look at the introduction of the NCLEX–RN in Canada. Can J Nurs Leadersh. 2019;32(4):46–56. doi:10.12927/cjnl.2020.26102

Reason for exclusion: Duplicate

92. Canadian Council of Registered Nurse Regulators. Competencies in the context of entry–level registered nurse practice – a report of the 2011–12 jurisdictional competency process: entry–level registered nurses [Internet]. 2012 [cited 2024 May 7]. Available from: https://www.ccrnr.ca/assets/jcp_rn_competencies_2012_edition.pdf
Reason for exclusion: Wrong source

93. Canadian Institute of Health Information. Regulated nurses: Canadian trends, 2007 to 2011. Ottawa. 2013. [cited June 20, 2014]. Available from: http://secure.cihi.ca/cihiweb/products/nursing_report_2007–2011_en.pdf.
Reason for exclusion: Wrong concept

94. Canadian Institute for Health Information. Regulated nurses in Canada: what do we know about them? Alta RN. 2011;67(5):10–1.
Reason for exclusion: Wrong concept

95. Cannon CA, Paulanka BJ, Beam S. A statewide assessment of preferences of registered nurses desiring academic credit–bearing continuing education. J Prof Nurs, 1994 Jul–Aug;10(4): 229–35. doi:10.1016/8755–7223(94)90024–8
Reason for exclusion: Wrong population

96. Carney M. Regulation of advanced nurse practice: its existence and regulatory dimensions from an international perspective. J Nurs Manag. 2016;24(1):105–14. doi:10.1111/jonm.12278
Reason for exclusion: Wrong source

97. Carter BE. e–business for nurse regulation. In: Proceedings of the 8th International Congress in Nursing Informatics; 2003 Jun 20–25; Rio de Janeiro, Brazil. 2003. p. 366–9.
Reason for exclusion: Wrong source

98. Cartmel V. Assessing an applicant's competencies to practice. Nurs BC. 2009;41(1):4.
Reason for exclusion: Wrong source

99. Carty E, Tait J. The midwifery debate: should non–nurses be allowed to practice midwifery? RNABC News. 1987;19(5):15–7.
Reason for exclusion: Wrong source

100. Chappell K, ElChamaa R, Jeong D, Kendall–Gallagher D, Salt E, Reeves S, et al. Conceptualization and operationalization of certification in the US and Canadian nursing literature. J Nurs Admin. 2018 May;48(5):238–46. doi:10.1097/NNA.0000000000000608
Reason for exclusion: Wrong source

101. Clarke SP, Patrician PA. Entry into practice in Ontario. Am J Nurs. 2001;101(2):73–6. doi:10.1097/00000446–200102000–00055
Reason for exclusion: Wrong concept

102. Coburn D. The development of Canadian nursing: professionalization and proletarianization. Int J Soc Determ Health Health Serv. 1988;18(3):437–56. doi:10.2190/1BDV–P7FN–9NWF–VKVR
Reason for exclusion: Wrong concept

103. College of Nurses of Ontario. Governance reform [Internet]. Toronto: College of Nurses of Ontario; n.d. [cited 2024 May 1]. Available from: https://www.cno.org/globalassets/trending–topics/cno–governance–reform.pdf
Reason for exclusion: Wrong source

104. College of Nurses of Ontario. Summary report – survey: jurisdictional governance review [Internet]. Toronto: College of Nurses of Ontario; 2016 Jan [cited 2024 May 1]. Available from: https://cno.org/Assets/CNO/Documents/What–is–CNO/jurisdictional–survey––summary–report.pdf
Reason for exclusion: Wrong source

105. College of Nurses of Ontario. Governance literature review [Internet]. Toronto: College of Nurses of Ontario; 2016 Nov [cited 2024 May 1] p. 132–42. Available from: https://cno.org/Assets/CNO/Documents/What–is–CNO/governance–literature–review––updated–november–2016.pdf
Reason for exclusion: Wrong source

106. College of Nurses of Ontario, Governance Task Force. Trends in regulatory governance [Internet]. Toronto: College of Nurses of Ontario; 2016 [cited 2024 May 1]. Available from: https://cno.org/Assets/CNO/Documents/What–is–CNO/trends–is–regulatory–governance.pdf
Reason for exclusion: Wrong source

107. Commission on the Future of Health Care in Canada. Building on values: the future of health care in Canada – final report. Ottawa: Government of Canada. 2002 Nov 1. Available from: https://publications.gc.ca/collections/Collection/CP32–85–2002E.pdf
Reason for exclusion: Wrong concept

108. Covell CL, Primeau M–D, Kilpatrick K, St–Pierre I. Internationally educated nurses in Canada: predictors of workforce integration. Hum Resour Health. 2017;15(1):26. doi:10.1186/s12960–017–0201–8
Reason for exclusion: Wrong concept

109. Covell CL, Neiterman E, Bourgeault IL. A scoping review of the literature on internationally educated nurses in Canada: mapping a research agenda. Can J Nurs Res. 2014;46(3):26–45. doi:10.1177/084456211404600303
Reason for exclusion: Wrong source

110. Curnew DR, Lukewich J. Nursing within primary care settings in Atlantic Canada: a scoping review. SAGE OPEN. 2018;8(2). doi:10.1177/2158244018774379
Reason for exclusion: Wrong source

111. Cutshall P. Cross border nursing. Nurs BC. 1993;25(2):9–10.
Reason for exclusion: Wrong source

112. Cutshall P. Megatrends in professional regulation: towards the year 2000. Nurs BC. 1994;26(5):16–8.
Reason for exclusion: Wrong source

113. Cutshall P. Regulating nursing: a new chapter begins. Nurs BC. 1998;30(3):38–7.
Reason for exclusion: Wrong source

114. Daly WM, Carnwell R. Nursing roles and levels of practice: A framework for differentiating between elementary, specialist and advancing nursing practice. J Clin Nurs. 2003;12:158–67.
Reason for exclusion: Wrong population

115. DiCenso A. Roles, research, and resilience: the evolution of advanced practice nursing. Can Nurse. 2008;104(9):37–40.
Reason for exclusion: Wrong concept

116. Duffield C, Gardner G, Chang AM, Catling–Paull C. Advanced nursing practice: a global perspective. Collegian. 2009;16(2):55–62. doi:10.1016/j.colegn.2009.02.001
Reason for exclusion: Wrong source

117. Duke N. Exploring advanced nursing practice: past, present and future. Br J Nurs. 2012;21(17):10261030.
Reason for exclusion: Wrong population

118. Durcan R. Grey areas: a commentary on legal issues affecting professional regulation – measuring regulatory performance part 1 – recurring features. [Internet]. 2022 Dec [cited 2024 May 7]; Available from: https://www.sml–law.com/wp–content/uploads/2022/11/Greyar273.pdf
Reason for exclusion: Wrong concept

119. Durcan R. Measuring Regulatory Performance Part 2 – Less Common Features. Grey Areas: A Commentary on Legal Issues Affecting Professional Regulation [Internet]. 2023 Jan [cited 2024 May 7]; Available from: https://www.sml–law.com/wp–content/uploads/2023/01/Greyar274.pdf
Reason for exclusion: Wrong concept

120. Dussault G, Fournier M–A, Zanchetta MS, Kérouac S, Denis J–L, Bojanowski L, et al. The nursing labour market in Canada: review of the literature [Internet]. Montreal: Groupe de recherche interdisciplinaire en santé, Université de Montréal; 2001 Jan. Available from: http://www.santecom.qc.ca/BibliothequeVirtuelle/GRIS/2921954486.pdf
Reason for exclusion: Wrong source

121. Edmunds, M. Standardizing our status. Nurse Pract. 2002;27:66.
Reason for exclusion: Wrong source

122. Eisler K. Significant Milestone for our profession – computer–adaptive RN entry exam. SRNA News Bull. 2012;14(2):4–5.
Reason for exclusion: Wrong source

123. Ellerbe S, Regen D. Responding to health care reform by addressing the institute of medicine report on the future of nursing. Nurs Admin Q. 2012 Jul–Sep;36(3):210–6. doi:10.1097/NAQ.0b013e318258bfa7
Reason for exclusion: Wrong concept

124. Ellis J. Using your title. Nurs BC. 1999;31(1):11–2.
Reason for exclusion: Wrong source

125. Ewart ME. Licensing Canadian nurses: the perspective from New York. Issues (National Council of State Boards of Nursing). 1985;6(1):7–8.
Reason for exclusion: Wrong concept

126. Fairman JA, Rowe JW, Hassmiller S, Shalala DE. Broadening the scope of nursing practice. N Engl J Med. 2011;364 (3):193–6.
Reason for exclusion: Wrong population

127. Fealy GM, et al. Models of initial training and pathways to registration: a selective review of policy in professional regulation. J Nurs Manag. 2009;17(6):730–8.
Reason for exclusion: Wrong population

128. Fitz–Gerald B. National Council Licensure Examination for registered nurses (NCLEX–RN). SRNA News Bull. 2014;16(4):9.
Reason for exclusion: Wrong source

129. Flaherty MJ. An examination of a national testing service. Can Nurse. 1968;64(6):48–53.
Reason for exclusion: Wrong concept

130. Fournier C, Blanchet Garneau A, Pepin J. Understanding the expanded nursing role in indigenous communities: A qualitative study. J Nurs Manag. 2021;29(8):2489–98. doi:10.1111/jonm.13349
Reason for exclusion: Wrong concept

131. Gardner G, Chang A, Duffield C. Making nursing work: breaking through the role confusion of advanced practice nursing. J Adv Nurs. 2007;57(4):382–91.
Reason for exclusion: Wrong concept

132. Gardner G, Gardner A, Middleton S, Della P, Kain V, Doubrovsky A. The work of nurse practitioners. J Adv Nurs. 2010;66(10):2160–9.
Reason for exclusion: Wrong concept

133. Giblin C, Lemermeyer G, Cummings G, Wang M, Kwan JA. Learning from experience: improving the process of internationally educated nurses' application for registration – a study protocol. J Adv Nurs. 2016;72(3):650–7. doi:10.1111/jan.12859
Reason for exclusion: Wrong source

134. Gill FJ, Leslie GD, Grech C, Latour JM. A review of critical care nursing staffing, education and practice standards. Aust Crit Care. 2012;25(4):224–37. doi:10.1016/j.aucc.2011.12.056
Reason for exclusion: Wrong concept

135. Gillespie L. Beyond imagining: A nursing perspective on the future health–care system. BCMJ. 2000;42(5):244–5.
Reason for exclusion: Wrong concept

136. Gilman DJ, Fairman, J. Antitrust and the future of nursing: federal competition policy and scope of practice. Health Matrix. 2014;24:143–208.
Reason for exclusion: Wrong population

137. Glasgow VM. Race and employment equity in nursing leadership: perceptions of racialized and non–racialized registered nurses [Internet]. Ann Arbor (MI): ProQuest Information & Learning; 2019.
Reason for exclusion: Wrong concept

138. Goudreau KA, Baldwin K, Clark A, Fulton J, Lyon B, Murray T, et al. A vision of the future for clinical nurse specialists [Internet]. Harrisburg (PA): National Association of Clinical Nurse Specialists; 2007 Jul. Available from: https://nacns.org/wp–content/uploads/2016/11/AVisionCNS.pdf

Reason for exclusion: Wrong population

139. Grant AE. Facsimile transmissions. Can Nurse. 1996;92(4):47.
Reason for exclusion: Wrong concept

140. Gregory DM. Acute care reform and its implications for health system and provider outcomes. 2007.
Reason for exclusion: Wrong concept

141. Guerrette–Daigle L, Landry V, Harrison S, Durocher–Hendriks S, Marquis FL, Auffrey LM, Maillet R, Labrie ND, McGraw M, Wallace I. The NCLEX–RN as an entry–to–practice exam in New Brunswick: the rocky road story of Francophone candidates to the nursing profession. Nurs Leadersh. 2019;32,74–80. doi:10.12927/ cjnl.2020.26099
Reason for exclusion: Duplicate

142. Guillemin EJ. Implementation of CNA standards for nursing administration [dissertation]. Edmonton (CA): University of Alberta; 1990. Available from: https://doi.org/10.7939/R3TB0Z423
Reason for exclusion: Wrong concept

143. Gunn V, Muntaner C, Ng E, Villeneuve M, Gea–Sanchez M, Chung H. The influence of welfare state factors on nursing professionalization and nursing human resources: a time–series cross–sectional analysis, 2000–2015. J Adv Nurs. 2019;75(11):2797–810. doi:10.1111/jan.14155
Reason for exclusion: Wrong concept

144. Hadley M. Nursing practice in Canada: the influence of current and proposed legislation. J Adv Nurs. 1995;22:1210–7.
Reason for exclusion: Duplicate

145. Haines J. Concern for public safety. Can Nurse. 1997;93(2):3.
Reason for exclusion: Wrong concept

146. Haines J. The nurse practitioner: A discussion paper. Ottawa: Canadian Nurses Association. 1993.
Reason for exclusion: Wrong source

147. Hamilton L. Serving the public interest is a balancing act. Can Nurse. 2008;104(6):48.
Reason for exclusion: Wrong concept

148. Hanrahan G., Sweeney J., Williams D. Service and professional regulators –moving from understanding to structured interaction for enhanced patient care. International Journal for Quality in Health Care. 2017;29(Supplement 1):28. doi:10.1093/intqhc/mzx125.42

Reason for exclusion: Wrong concept

149. Hanson CM, Hamric AB. Reflections on the continuing evolution of advanced practice nursing. Nurs Outlook. 2003;51(5):203–11.
Reason for exclusion: Wrong population

150. Hartness C. Interdisciplinary co–operation in health care. 2007.
Reason for exclusion: Wrong concept

151. Heale R, Rieck Buckley C. An international perspective of advanced practice nursing regulation. Int Nurs Rev, 2015;62(3):421–9.
Reason for exclusion: Duplicate

152. Heitlinger A. Nurses and nursing: A comparative perspective. J Interprof Care. 1999;13(2):165–74. doi:10.3109/13561829909025548
Reason for exclusion: Wrong concept

153. Hickey JV, Unruh LY, Newhouse RP, et al. Credentialing: the need for a national research agenda. Nurs Outlook. 2014; 62(2):119–27.
Reason for exclusion: Wrong population

154. Human Resources and Skills Development Canada. Summative evaluation of the foreign credential recognition program: final report [Internet]. Gatineau (QC): Evaluation Directorate, Strategic Policy and Research Branch; 2010 Apr. Available from: https://publications.gc.ca/site/eng/370434/publication.html
Reason for exclusion: Wrong concept

155. Hurlock–Chorostecki C, van Soeren M, Goodwin S. The acute care nurse practitioner in Ontario: a workforce study. Can J Nurs Leadersh. 2008;21(4):100–16.
Reason for exclusion: Duplicate

156. Hutton D. Executive director's message. Alta RN. 2001;2–3.
Reason for exclusion: Wrong source

157. Jauregui AB, Xu Y. Transition into practice: experiences of Filipino physician–turned nurse practitioners. J Transcult Nurs, 2010;21:257–64. doi:10.1177/1043659609358787
Reason for exclusion: Wrong population

158. Joel LA. Your license to practice: variations on a theme. Am J Nurs. 1995;95(11):7.
Reason for exclusion: Wrong concept

159. Johansen C, Knowles B, Jones D, Dickison P. Commentary on McGillis Hall L, Lalonde M, Kashin J, Yoo C, Moran J (2017) Changing nurse licensing examinations: media analysis

and implication of the Canadian experience. Int Nurs Rev. 2018 Mar;65(1):12–4. doi:10.1111/inr.12445.
Reason for exclusion: Wrong concept

160. Johnson G. Regulating midwifery. Parallels in Texas and British Columbia. Midwifery Today Int Midwife. 2000;(56):29–31.
Reason for exclusion: Wrong population

161. Karim R. Nursing in Canada: registered nurses, 1992. Health Rep. 1993;5(2):229–32.
Reason for exclusion: Wrong source

162. Kellett P, Fitton C. Supporting transvisibility and gender diversity in nursing practice and education: embracing cultural safety. Nurs Inq. 2017;24(1). doi:10.1111/nin.12146
Reason for exclusion: Wrong concept

163. Kendall MD. The potential role of the licensed practical nurse as an alternate caregiver in the acute care setting of Children's and Women's Health Centre of British Columbia. 2003.
Reason for exclusion: Wrong concept

164. Kerr JC. Point of view: the entry into practice issue – the shouting starts. Can Nurse. 1982;78(2):42–3.
Reason for exclusion: Wrong concept

165. Khaliq AA, Broyles RW, Mwachofi AK. Global nurse migration: its impact on developing countries and prospects for the future. Nurs Leadersh. 2009;22(1), 24–50.
Reason for exclusion: Wrong concept

166. Kleinpell RM. Acute care nurse practitioner practice: results of a 5 year longitudinal study. Am J Crit Care. 2005;14:211–9.
Reason for exclusion: Wrong concept

167. Kolawole B. Ontario's internationally educated nurses and waste in human capital. Int Nurs Rev. 2009;56(2):84–190.
Reason for exclusion: Duplicate

168. Lalonde M. The forgotten: the challenges faced by Francophone nursing candidates following the introduction of the NCLEX–RN in Canada. Nurs Leadersh. 2019;32:66–73. doi:10.12927/ cjnl.2020.26100.
Reason for exclusion: Duplicate

169. Lalonde M, Chartrand J, Vanderspank–Wright B, McGillis Hall L, Gosselin–Bélanger A, Fullum JA. Les effets potentiels de la version française du NCLEX–RN® sur le choix de la

langue de formation d'étudiantes infirmières francophones en situation minoritaire. QANE–AFI. 2020;6(1):Article 7. doi:10.17483/2368–6669.1181
Reason for exclusion: Wrong language

170. Lalonde M, Prairie G, Vanderspank–Wright B, Chartrand J, Mcgillis Hall L, Lamont M. NCLEX–RN© preparation resources available online in French: an integrative review. Int Nurs Review. 2022;69(2):211–20. doi:10.1111/inr.12705
Reason for exclusion: Wrong source

171. Lamb C, Evans M, Babenko–Mould Y, Wong C, Kirkwood K. Nurses' use of conscientious objection and the implications for conscience. J Adv Nurs. 2019;75(3):594–602. doi:10.1111/jan.13869
Reason for exclusion: Wrong concept

172. Lesa R, Dixon A. Physical assessment: implications for nurse educators and nursing practice. Int Nurs Review. 2007;54(2):166–72. doi:10.1111/j.1466–7657.2007.00536.x
Reason for exclusion: Wrong source

173. Lloyd Jones M. A brief history of the registration of nurses. British Journal of Healthcare Assistants. 2012;6(1):41–4.
Reason for exclusion: Wrong population

174. Lopes–Junior LC. Advanced practice nursing and the expansion of the role of nurses in primary health care in the Americas. SAGE Open Nurs. 2021;7. doi:10.1177/23779608211019491
Reason for exclusion: Wrong concept

175. Lukewich J, Poitras ME, Mathews M. Unseen, unheard, undervalued: advancing research on registered nurses in primary care. Practice Nursing. 2021;32(4):158–62. doi:10.12968/pnur.2021.32.4.158
Reason for exclusion: Wrong concept

176. Lum L, Dowedoff P, Englander K. Internationally educated nurses' reflections on nursing communication in Canada. Int Nurs Review. 2016;63(3):344–51. doi:10.1111/inr.12300
Reason for exclusion: Duplicate

177. Lyons B. The CNS regulatory quagmire: We need clarity about advanced nursing practice. Clin Nurs Spec. 2004;18(1):9–13.
Reason for exclusion: Wrong population

178. MacDonald JA, Herbert R, Thibeault C. Advanced practice nursing: unification through a common identity. J Prof Nurs. 2006;22(3):172–9. doi:10.1016/j.profnurs.2006.03.009

Reason for exclusion: Wrong concept

179. MacDonald M, Schreiber R, Davis L. Exploring new roles for advanced nursing practice: Examen des nouveaux rôles en pratique infirmière avancée: document de discussion [Internet]. Canadian Nurses Association; 2005 [cited 2024 May 8].
Reason for exclusion: Wrong source

180. MacLeod MLP, Stewart NJ, Kosteniuk JG, Penz KL, Olynick J, Karunanayake CP, et al. Rural and remote registered nurses' perceptions of working beyond their legislated scope of practice. Nurs Leadersh. 2019 Mar;32(1):20–9. doi:10.12927/cjnl.2019.25851
Reason for exclusion: Wrong concept

181. Martin–Misener R, Bryant–Lukosius D, Bullard C, Campbell D, Carter N, Donald F, et al. Optimizing the role of nurses in primary care in Canada: final report. Ottawa (ON): Canadian Nurses Association; 2014 Aug. Available from https://cna–aiic.ca/~/media/cna/page–content/pdf–en/optimizing–the–role–of–nursesin–primary–care–in–canada.pdf
Reason for exclusion: Wrong concept

182. Matiti R, Taylor D. The cultural lived experience of internationally recruited nurses: A phenomenological study. Diversity in Health and Social Care. 2005;2:7–16.
Reason for exclusion: Wrong population

183. Matheson S, Svendsen A. Certification in cardiovascular nursing. Can J Cardiovasc Nurs. 2003;13(3):4–6.
Reason for exclusion: Wrong concept

184. McAuliffe MS, Henry B. Survey of nurse anesthesia practice, education, and regulation in 96 countries. J Am Assoc Nurs Anesth. 1998;66:273–86.
Reason for exclusion: Wrong population

185. McGillis Hall L, Lalonde M, Kashin J. People are failing! something needs to be done: Canadian students' experience with the NCLEXRN. Nurse Educ Today. 2016;46:43–9. doi:10.1016/j.nedt.2016.08.022.
Reason for exclusion: Duplicate

186. McGillis Hall L, Gates M, Peterson J, Jones C, Pink GH. Waiting and watching: nurse migration trends before a change to the National Council Licensure Examination as entry to practice for Canada's nurses. Nurs Outlook. 2014;62(1):53–8. DOI:10.1016/j.outlook.2013.11.003
Reason for exclusion: Wrong concept

187. McGillis Hall L, Gates M, Peterson J, Jones CB, Pink GH. Waiting and watching: Nurse migration trends before a change to the National Council Licensure Examination as entry to practice for Canada's nurses. Nurs Outlook. 2014;62(1):53–8. DOI:10.1016/j.outlook.2013.11.003
Reason for exclusion: Duplicate

188. McGuire M., Murphy S. The internationally educated nurse. Can Nurse. 2005;101(1):25–9.
Reason for exclusion: Duplicate

189. McHugh MD, Hawkins RE, Mazmanian PE, Romano PS, Smith HL, Spetz J. Challenges and opportunities in nurse credentialing research design [Internet]. Washington (DC): Institute of Medicine; 2014 Aug 19. Available from: https://nam.edu/wp–content/uploads/2015/06/CredentialingResearchDesign.pdf
Reason for exclusion: Wrong concept

190. McIntosh T, Torgerson R, Klassen N. The ethical recruitment of internationally educated health professionals: lessons from abroad and options for Canada. Ottawa: Canadian Policy Research Networks; 2007 Jan. Available from: https://oaresource.library.carleton.ca/cprn/46781_en.pdf
Reason for exclusion: Wrong concept

191. McIntyre M, McDonald C. Realities of Canadian nursing: professional, practice, and power issues. 4th ed. Philadelphia (PA): Wolters Kluwer Health; 2014.
Reason for exclusion: Wrong source

192. Mildon B. Voices Raised: The NCLEX–RN Experience in Canada. Nurs Leadersh (Toronto, Ont). 2019;32(4):1–6. doi:10.12927/cjnl.2020.26107
Reason for exclusion: Wrong source

193. Moyce S, Lash R, Siantz M. Migration experiences of foreign educated nurses: a systematic review of the literature. J Transcult Nurs. 2016;27(2):181–8. doi:10.1177/1043659615569538
Reason for exclusion: Wrong source

194. Tomblin Murphy G, Sampalli T, Bourque Bearskin L, Cashen N, Cummings G, Elliott Rose A, et al. Investing in Canada's nursing workforce post–pandemic: a call to action. FACETS. 2022 Aug;7:1051–120. doi:10.1139/facets–2022–0002
Reason for exclusion: Wrong concept

195. Neiterman E, Bourgeault IL. Cultural competence of internationally educated nurses: assessing problems and finding solutions. Can J Nurs Res. 2013 Dec;45(4):88–107. doi:10.1177/084456211304500408

Reason for exclusion: Duplicate

196. Newton S, Pillay J, Higginbottom G. The migration and transitioning experiences of internationally educated nurses: a global perspective. J Nurs Manag. 2012 May;20(4):534–50. doi:10.1111/j.1365–2834.2011.01222.x
Reason for exclusion: Wrong source

197. O'Flynn–Magee K, Hall W, Segaric C, Peart J. Guest editorial: the impact of COVID–19 on clinical practice hours in pre–licensure registered nurse programs. Teach Learn Nurs. 2021;16(1):3–4. doi:10.1016/j.teln.2020.07.007
Reason for exclusion: Wrong concept

198. Paterson BL, Duffett–Leger L, Cruttenden K. Contextual factors influencing the evolution of nurses' roles in a primary health care clinic. Public Health Nurs. 2009 Sep–Oct;26(5):421–9. doi:10.1111/j.1525–1446.2009.00800.x
Reason for exclusion: Wrong concept

199. Pauly B, Schreiber R, MacDonald M, Davidson H, Crickmore J, Moss L, et al. Dancing to our own tune: understandings of advanced nursing practice in British Columbia. Nurs Leadersh. 2004 May;17(2):47–59. doi:10.12927/cjnl.2004.16346
Reason for exclusion: Wrong concept

200. Pesut B, Greig M. Resources for educating, training, and mentoring nurses and unregulated nursing care providers in palliative care: a review and expert consultation. J Palliat Med. 2018 Jan;21(S1):S50–6. doi:10.1089/jpm.2017.0395
Reason for exclusion: Wrong source

201. Pesut B, Thorne S, Chambaere K, Hall M, Schiller CJ. The evolving complexities of MAID care in Canada from a nursing perspective. Glob Qual Nurs Res. 2024;11. doi:10.1177/23333936241228233
Reason for exclusion: Wrong concept

202. Pesut B, Thorne S, Greig M, Fulton A, Janke R, Vis–Dunbar M. Ethical, policy, and practice implications of nurses' experiences with assisted death: a synthesis. Adv Nurs Sci. 2019;42(3):216–30. doi:10.1097/ANS.0000000000000276
Reason for exclusion: Wrong source

203. Schreiber R, MacDonald M, Davidson H, Crickmore J, Moss L, Pinelli J, Regan S, Pauly B, Hammond C. Advanced nursing practice: opportunities and challenges in British Columbia. Victoria (BC): University of Victoria; 2003 Apr.
Reason for exclusion: Duplicate

204. Prescott C, Shahram SZ, Ogilvie G, Hassam N, Franks AS, Pauly B. Applying a health equity tool to assess a public health nursing guideline for practice in sexually transmitted infection assessment in British Columbia. Can J Public Health. 2020;111:610–6. doi:10.17269/s41997–019–00285–2
Reason for exclusion: Wrong concept

205. Pulcini J, Jelic M, Gul R. Loke AY. An international survey on advanced practice nursing education, practice, and regulation. J Nurs Scholarsh. 2010;42(1):31–9.
Reason for exclusion: Duplicate

206. Rajabally MH. Point of view: the entry to practice issue. Can Nurse. 1982;78(2):40–2.
Reason for exclusion: Wrong concept

207. Rankin JM. How nurses practice health care reform: an institutional ethnography [dissertation]. Victoria (BC): University of Victoria; 2004. Available from: http://hdl.handle.net/1828/345
Reason for exclusion: Wrong concept

208. Redekopp MA. Clinical nurse specialist role confusion: the need for identity. Clin Nurs Spec. 1997;11(2):87–91.
Reason for exclusion: Wrong concept

209. Richardson S. Articulation and baccalaureate entry to practice: the Canadian context. Nurs Pap. 1986;18(3):47–58.
Reason for exclusion: Wrong concept

210. Ronquillo C, Boschma G, Wong ST, Quiney L. Beyond greener pastures: exploring contexts surrounding Filipino nurse migration in Canada through oral history. Nurs Inq. 2011;18(3):262–75.
Reason for exclusion: Wrong concept

211. Ronquillo C, Boschma G, Wong ST, Quiney L. Beyond greener pastures: exploring contexts surrounding Filipino nurse migration in Canada through oral history. Nurs Inq. 2011;18:262–75. doi:10.1111/j.1440–1800.2011.00545.x
Reason for exclusion: Duplicate

212. Ronquillo C. Leaving the Philippines: oral histories of nurses' transition to Canadian nursing practice. Can J Nurs Res. 2012;44:96–115.
Reason for exclusion: Wrong concept

213. Rooks JR, Ernst EKM. The future of midwifery. Int J Childbirth Educ. 1999;14(4):16–21.
Reason for exclusion: Wrong population

214. Rose B, All A, Gresham D. Role preservation of the clinical nurse specialist and the nurse practitioner. The Internet Journal of Advanced Nursing Practice. 2003;5(2):1–11.
Reason for exclusion: Wrong population

215. Rose SB, All AC, Gresham D. Role preservation of the clinical nurse specialist and the nurse practitioner. The Internet Journal of Advanced Nursing Practice. 2003;5(2):1–11.
Reason for exclusion: Duplicate

216. Ross CA. Public protection as a ruling concept in the management of nurses' substance use. In: Luken PC, Vaughan S, editors. The Palgrave handbook of institutional ethnography. Cham: Palgrave Macmillan; 2021. p. 423–46. Available from: https://doi.org/10.1007/978–3–030–54222–1_22
Reason for exclusion: Wrong source

217. Rounds LR, Zych JJ, Mallary LL. The consensus model for regulation of APRNs: implications for nurse practitioners. J Am Acad Nurse Pract. 2013;25(4):180–5.
Reason for exclusion: Wrong population

218. Russell KA. Caring for patients using medical marijuana. J Nurs Regul. 2019 Oct;10(3):47–61. doi:10.1016/S2155–8256(19)30148–6
Reason for exclusion: Wrong concept

219. Safriet BJ. Health care dollars and regulatory sense: the role of advanced practice nursing. Yale J Regul. 1992;9:419–84.
Reason for exclusion: Wrong population

220. Salami B, Nelson S. The downward occupational mobility of internationally educated nurses to domestic workers. Nurs Inq. 2014;21(2):153161. doi:10.1111/inr.12125
Reason for exclusion: Wrong concept

221. Salami B, Nelson S, Hawthorne L, Muntaner C, McGillis Hall L. Motivations of nurses who migrate to Canada as domestic workers. Int Nurs Review. 2014;61(4):479–86.
Reason for exclusion: Wrong concept

222. Salma J, Hegadoren K, Ogilvie L. Career advancement and educational opportunities: experiences and perceptions of internationally educated nurses. Nurs Leadersh, 2012;25(3):56–67.
Reason for exclusion: Wrong concept

223. Sangster–Gormley E, Martin–Misener R, Downe–Wamboldt B, DiCenso A. Factors affecting nurse practitioner role implementation in Canadian practice settings: an integrative review. J Adv Nurs. 2011;67:1178–90. doi:10.1111/j.1365–2648.2010.05571.x

Reason for exclusion: Wrong source

224. Schiller CJ, Pesut B, Roussel J, Greig M. But it's legal, isn't it? Law and ethics in nursing practice related to medical assistance in dying. Nurs Philos. 2019;20(4):e12277. doi:10.1111/nup.12277
Reason for exclusion: Wrong concept

225. Schober M. The global emergence of advanced practice nurses in providing home and community care health services. Home Health Care Manag Pract. 2007;20(1):34–40.
Reason for exclusion: Wrong concept

226. Schober M, Affara F. International Council of Nurses: advanced nursing practice. Oxford: Blackwell Publishing; 2006.
Reason for exclusion: Wrong source

227. Shaffer F, To Dutka J. Global mobility for internationally educated nurses: challenges and regulatory implications. J Nurs Regul. 2013;4(3):11–6.
Reason for exclusion: Wrong population

228. Sibbald B. Delegating away patient safety. Can Nurse. 1997;93(2):22–6.
Reason for exclusion: Wrong concept

229. Singh MD, Sochan A. Voices of internationally educated nurses: policy recommendations for credentialing. Int Nurs Review 2010;57(1):56–63.
Reason for exclusion: Duplicate

230. Smith MC. The core of advanced practice nursing. Nurs Sci Q. 1995;8:2–3. doi:10.1177/089431849500800102
Reason for exclusion: Wrong concept

231. Sochan A, Singh MD. Acculturation and socialization "voices" of internationally educated nurses in Ontario. International Journal of Nurses. 2007;45:130–6.
Reason for exclusion: Duplicate

232. Solomon J. Physical assessment skills in undergraduate curricula. Nurs Outlook. 1990;38(4):194–5.
Reason for exclusion: Wrong concept

233. Spector M, LeBlanc B, Danson N, Ismail F. A case that shaped how regulators govern in Ontario. J Nurs Regul. 2015;6(2):43–6.
Reason for exclusion: Wrong population

234. St–Denis V. The NCLEX–RN and the future of French–language health–care services. Can Nurse. 2017 Sep–Oct;113(5):28–31. Available from: https://www.canadian–nurse.com/viewdocument/septemberoctober–2017?CommunityKey=d320003f–c6d8–4209–a9e7–4fbff8be6289&tab=librarydocuments
Reason for exclusion: Wrong source

235. Stanley JM, Werner KE, Apple K. Positioning advanced practice registered nurses for health care reform: consensus on APRN Regulation. J Prof Nurs. 2009;25(6):340–8.
Reason for exclusion: Wrong population

236. Stasa H, Cashin A, Buckley T, Donoghue J. Advancing advanced practice – clarifying the conceptual confusion. Nurse Educ Today. 2014;34(3):356–61. doi:10.1016/j.nedt.2013.07.012
Reason for exclusion: Wrong concept

237. Stievano A, Caruso R, Pittella F, Shaffer FA, Rocco G, Fairman J. Shaping nursing profession regulation through history – a systematic review. Int Nurs Review. 2019;66(1):17–29. doi:10.1111/inr.12449
Reason for exclusion: Wrong source

238. Sweatman L. Reaching milestones in regulation. Can Nurse. 2007;103(4):40.
Reason for exclusion: Wrong concept

239. Sweatman L. Models in professional regulation: choices for Atlantic Canada? J Med Regul . 2023;109(1):22–8. doi:10.30770/2572–1852–109.1.22
Reason for exclusion: Wrong population

240. Thompson, A. The relationship of the legalization of midwifery and safe motherhood. Midwifery. 2003;19(2):77–8.
Reason for exclusion: Wrong population

241. Tomblin–Murphy G, Birch S, Alder R, MacKenzie A, Lethbridge L, Little L, Cook A. Tested solutions for eliminating the Canadian registered nurses shortage. Ottawa (ON): Canadian Nurses Association; 2009.
Reason for exclusion: Wrong concept

242. Turrittin J, Hagey R, Gurgue S, Collin E, Mitchell M. The experiences of professional nurses who have migrated to Canada: cosmopolitan citizenship or democratic racism? Int J Nurs Stud. 2002;39(6):655–67.
Reason for exclusion: Wrong concept

243. Velji K. Under examination. Can Nurse. 2015;111(8):4.
Reason for exclusion: Wrong concept

244. Villegas WJ, Allen PE. Barriers to advanced practice registered nurse scope of practice: issue analysis. J Contin Educ Nurs. 2012;43(9):403–9.
Reason for exclusion: Wrong population

245. Villeneuve M, Betker C, Guest T. Canadian Nurses Association: reflections on the NCLEX–RN through the lens of nursing history and evolution. Can J Nurs Leadersh. 2019;32(4):17–21. doi:10.12927/cjnl.2020.26105
Reason for exclusion: Duplicate

246. Villeneuve M, MacDonald JA. Toward 2020: visions for nursing. Can Nurse. 2006;102(5):22.
Reason for exclusion: Wrong concept

247. Westfall R. The state of midwifery in British Columbia, Canada. Midwifery Today Int Midwife. 2002;(62):51–5.
Reason for exclusion: Wrong population

248. Worster A, Sarco A, Thrasher C, Fernandes C, Chemeris E. Understanding the role of the nurse practitioner in Canada. Can J Rural Med. 2005;10(2):89–94.
Reason for exclusion: Duplicate
